# Emergence of fluorescent aggregates through hierarchical self-assembly

**DOI:** 10.1039/d5sc04688b

**Published:** 2025-10-16

**Authors:** Maëva Coste, Sébastien Ulrich

**Affiliations:** a Institut des Biomolécules Max Mousseron (IBMM), CNRS, Université de Montpellier, ENSCM Montpellier France sebastien.ulrich@cnrs.fr

## Abstract

Controlling the growth of functional supramolecular nano-structures in aqueous media is a current challenge both for developing soft materials and for understanding the emergence of complex macromolecules by self-organization. We investigated here the growth of systems combining a non-fluorescent water-soluble tetraphenylethene tetraaldehyde with complementary hydrazide partners, and found that fluorescent aggregates, identified through a combinatorial screening assay, emerge through a hierarchical self-assembly involving dynamic covalent self-assembly followed by supramolecular aggregation. The process is controlled, on one hand, by external (concentration, pH) and internal (nature of side-chain) factors which dictates the outcome of the self-assembly, while, on the other hand, the supramolecular self-assembly exerts, through a feed-back loop, component selection and auto-catalytic growth which was observed using a β-sheet-forming pentapeptide.

## Abstract 2

The combination of a non-fluorescent tetraphenylethene core with hydrazide partners leads to the hierarchical growth of fluorescent supramolecular nanostructures through dynamic covalent self-assembly followed by supramolecular polymerization.

## Graphical abstract

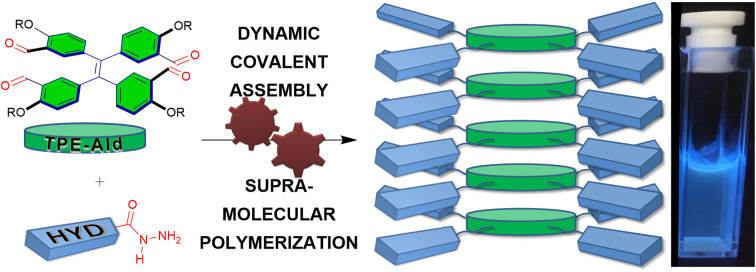

## Introduction

Supramolecular polymers, which result from the association of multiple monomers through non-covalent interactions such as hydrogen bond and π–π stacking, are interesting candidates as smart soft materials, but also as a case study for understanding the emergence of complex macromolecules by self-organization.^1^

Planar aromatics, such as the benzene triscarboxamides (BTAs) which self-assemble into columnar supramolecular polymers,^2^ have been at the fore-front of the field, but recent efforts have pushed the approach toward the exploration of non-planar core compounds.^3–8^ Indeed, the introduction of new cores is an entry toward functional supramolecular polymers, such as fluorescent supramolecular polymers.^9–14^ However, the nature of the side-groups has been found to play a critical role in the self-assembly, directing the dimensionality of the objects formed and also governing the mechanism of supramolecular polymerization.^15,16^

Tetraphenylethenes (TPEs)^17–19^ are non-planar polyaromatics that display an unusual aggregation-induced fluorescence emission (AIE effect).^20–23^ Some examples have reported their insertion into supramolecular polymers, mainly through hydrogen-bonding and metal-ion coordination.^24,25^ However, due to the propeller conformation of TPEs that prevent π–π stacking interactions,^26^ their organization within supramolecular aggregates, often mediated by weak C–H⋯π interactions, remains difficult to predict. Nevertheless, the columnar arrangement of multiple TPE derivatives, separated by long-range interactions (*ca.* 5–9 Å),^27–29^ has been reported in the solid state,^27,30^ in liquid crystals,^31,32^ as well as in organic solution.^33–35^ Due to the existence of competing and accessible self-assembly pathways, these aggregates are usually sensitive to environmental conditions – such as the nature of the medium,^27^ the application of mechanical forces that lead to mechanochromic fluorescence^31,35^ – and of course to molecular design. Potentially, side-groups can synergistically establish secondary interactions like in the pioneering example of BTAs where π–π stacking interactions and hydrogen bonds work together to trigger a cooperative supramolecular polymerization.^36^ TPEs bearing amide-linked aliphatic substituents have been shown to display AIE in organic solvents.^37^ Peptides are attractive side-groups and their introduction within supramolecular polymers is a promising strategy to stabilize supramolecular polymers and to impart water solubility which remains an important challenge.^38^ Hitherto, only a few studies investigated the self-assembly of TPEs conjugated with single amino acids.^28,35,39–42^

Dynamic covalent chemistry (DCvC) has recently gained momentum in peptide-based supramolecular polymers to reversibly connect cores with side-groups,^38,43^ revealing unusual properties such as chemically-controlled supramolecular polymerization^44–46^ and hydrogel formation,^47,48^ and self-replicating nanofibers formed by auto-catalysis.^49–52^ The latter is an inspiring contribution from the Otto group who uses disulphide bond formation – a process that usually takes days for the self-assembly to emerge – to form core-side-groups oligomers from which supramolecular polymer emerge. Acylhydrazone formation is another popular reversible covalent reaction involving the self-assembly of complementary building blocks. Yet, despite early notice of the propensity of acylhydrazones to engage in hydrogen-bond interactions,^53^ which are frequent in supramolecular polymers, their implementation in supramolecular polymerization processes has received little attention.^54–57^

**Fig. 1:**
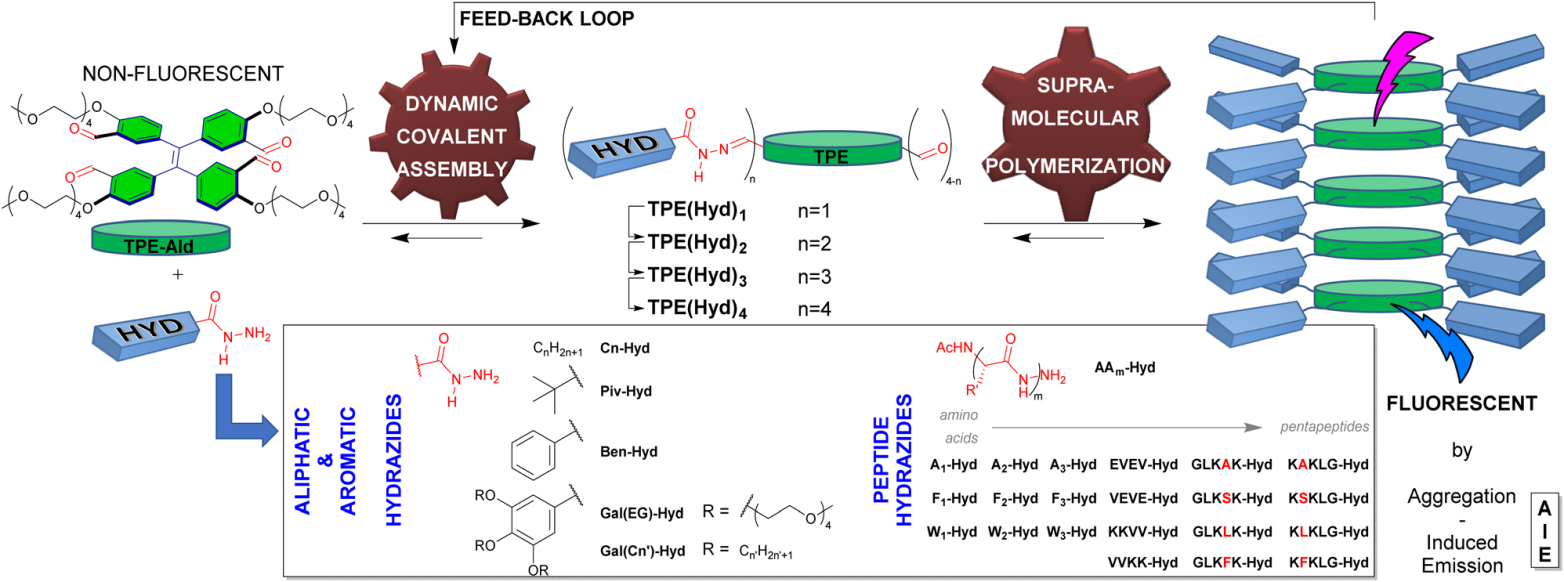
Conceptual representation of the study depicting the building blocks TPE-Ald and HYD which assemble through dynamic covalent self-assembly into TPE(Hyd)*_n_*, followed by supramolecular polymerization, yielding fluorescent aggregates. Results and discussion.

Herein, we report a case of *in situ* hierarchical self-assembly which combines a first step of dynamic covalent assembly with a subsequent supramolecular polymerization. When combining a water-soluble TPE tetra-aldehyde with complementary hydrazide partners, we show the emergence of fluorescence aggregates which formation is controlled by reaction conditions (stoichiometry, concentration, pH), thus affording a chemical control over the growth of fluorescent supramolecular polymers (Fig. 1). We also show that these supramolecular nanostructures can, in a feedback loop, exert a selective pressure on the dynamic covalent self-assembly step, leading to selection of the best constituent and to the auto-catalytic growth best observed at neutral pH using a tetrapeptide hydrazide partner.

## Results and discusssion

### Design

Our system rests on the use of a TPE-aldehyde core which is combined with complementary hydrazide side groups as triggers of the self-assembly. The TPE core shall be unable to undergo supramolecular polymerization on its own in a good solvent, and the multiple reactions with appropriate hydrazide partners shall then trigger, through new non-covalent interactions, the process of supramolecular polymerization (Fig. 1). Our rational is based on the fact that acylhydrazones can partake in amide-like hydrogen bonds,^53^ thus triggering supramolecular polymerization, while the additional existence of non-covalent interactions between side-chains would endow a partner-selectivity favouring the self-assembly only in the presence of the best hydrazide. In order to perform the self-assembly in aqueous media, we designed TPE-Ald featuring four aldehyde groups and four neutral hydrophilic tetra-ethylene glycol monomethylether chains.

### Synthesis

TPE-Ald was synthesized in two steps from the commercially-available tetrakis(4-hydroxyphenyl)ethylene through a Duff tetra-formylation followed by a Williamson tetra-ether formation (see SI). A complementary library of 36 aliphatic (C*n*-Hyd, Piv-Hyd), aromatic (Ben-Hyd, Gal(EG)-Hyd, Gal(C*n*′)-Hyd) and peptide hydrazides (AA*_n_*-Hyd) was selected, using commercially-available hydrazides or C-hydrazide peptides bearing sequences promoting the formation of β-sheets: FF,^58^ VEVE,^46^ KKLL,^59^ and GLSXK^50–52^ with X = A, S, L, F. These C-hydrazide peptides were synthesized through a Fmoc-based solid-phase peptide synthesis using a modified Fmoc-hydrazine resin, as previously reported.^60–62^ Their N-termini were acetylated to discard the possibility of imine formation at this position. In addition, and since the position of the aromatic at the C or N terminus may affect the outcome of the self-assembly,^63,64^ each reverse peptide sequence was also synthesized and tested.

### Screening

**Fig. 2:**
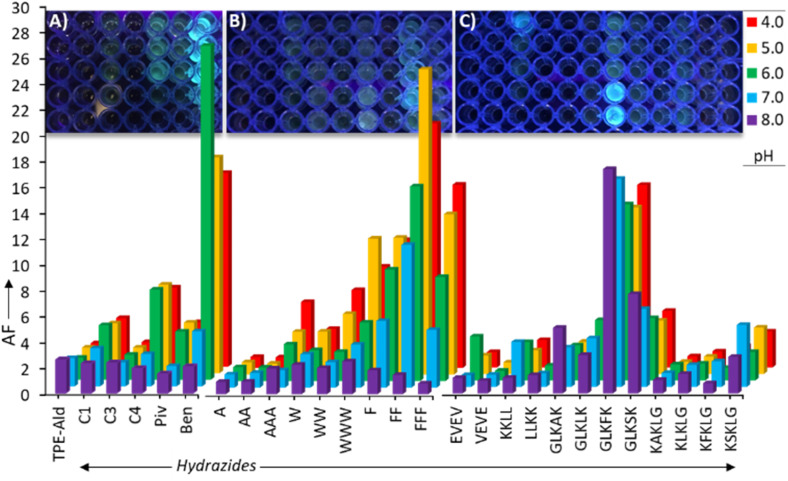
Combinatorial screening in 96-well plates detecting fluorescence enhancement (amplification factor AF) when combining TPE-Ald (0.3 mM) with different hydrazides ((A): aliphatics; (B) hydrophobic peptides; (C) amphiphilic tetra/penta-peptides) at different pH. *λ*_exc_ = 330 nm, *λ*_em_ = 510 nm. Incubation time: 24 hours. The photographs of the plates were taken under UV-light irradiation.

With the aim of detecting which side-group promotes the formation of supramolecular polymers, we set up a combinatorial screening assay in 96-well plates using fluorescence spectroscopy as a read-out to detect combinations of TPE-Ald–hydrazide that promote aggregation-induction emission at different pH (4–8) in aqueous medium. The concentration was set at 0.3 mM in TPE-Ald, which was then mixed at room temperature with 4.0 equivalents of each hydrazide since tetraconjugates with C1-Hyd as a model were found to form within a couple of hours under these conditions (Fig. S45). The fluorescence data are expressed in terms of amplification factor AF, defined as AF = *F*^rxt^_em_/*F*^TPE-Ald^_em_, where *F*^rxt^_em_ represent the intensity of fluorescence emission measured at different time points after TPE-Ald and hydrazide had been mixed, and *F*^TPE-Ald^_em_ represents the initial fluorescence emission intensity of TPE-Ald measured prior to the addition of the hydrazide. An AF greater than 1 reveals an aggregation-induction emission during the course of the reaction, while an AF lower than 1 suggests aggregation-caused quenching. While caution must be taken when comparing the raw fluorescence emission at different pH as quantum yields can be affected, the results reveal marked differences depending on the nature of the hydrazides (Fig. 2). While the shortest aliphatic hydrazides C1- to C3-Hyd lead to weak AF comparable to that of TPE-Ald, the reaction with valeric acid hydrazide C4-Hyd showed an eight to twelve-fold increase in AF at pH = 5–7 (Fig. 2A). Longer aliphatic chains (C*n*-Hyd, *n* = 5–7) did not show enhanced AF and led to solubility issues (Fig. S46). Interestingly the isomeric pivaloic acid hydrazide Piv-Hyd showed much weaker AF < 3, suggesting that steric effects play a role in the observed AIE effects and limits supramolecular aggregation.^65^ On the other hand, benzhydrazide (Ben-Hyd) shows a much more pronounced AF = 23, along with a bell-shape dependency on pH – AF peaking at pH = 6. This observation of AIE promoted by Ben-Hyd suggests the existence of stabilizing π–π stacking interactions or the assistance of the hydrophobic effect. Unfortunately, using gallic hydrazides functionalized with aliphatic (Gal(C*n*′)-Hyd) or ethyleneglycol (Gal(EG)-Hyd) chains did not significantly boost this AF (AF_max_ ≈ 26, Fig. S46) and all samples appeared rapidly turbid. We then turned our attention to the C-hydrazide peptides that feature short sequences rich in aromatic amino acids or prone to form β-sheets. While no AIE was detected with A_1_-Hyd and A_2_-Hyd, a moderate AF = 7 was seen at acidic pH using A_3_-Hyd. Substituting the aliphatic for aromatic amino acids F*_n_*-Hyd and W*_n_*-Hyd significantly enhanced AF, reaching AF = 25 with F_2_-Hyd at pH = 5.0. Within the series of the longer tetra- and penta-peptides, only two peptides, GLKFK-Hyd and GLKSK-Hyd really stood out, displaying AF_max_ = 17 and 7. The superiority of the former is in line with previous reports from the Otto group,^66,67^ who followed a different design where the aromatic core was tethered at the N-terminus of the peptide, and confirm the potential role of π–π stacking interactions in our system. Surprisingly, these two peptide hits turned out to trigger AIE over the whole pH range (4–8). Finally, all the reverse sequences showed no significant AIE. Overall, the screening successfully revealed some trends (role of aliphatic chains, importance of aromatics, effect of pH), which should arise from secondary interactions during supramolecular polymers growth (π–π stacking, β-sheets, hydrophobic shielding). Indeed, dynamic covalent conjugates of TPE-Ald were formed in all cases but only a few of them exhibited AIE effect in solution. This observation rules out the rigidification explanation for the appearance of the enhanced fluorescence emission, which should have been a general feature throughout the screening, and supports an aggregation-induced phenomenon.^68,69^ The most striking case is the comparison, within the pentapeptide series, of the GLKFK and KFKLG isomer peptides: only the former and not the latter give rise to a significant fluorescence emission enhancement (AF_max_ = 17). In the end, this screening led to the identification of three hits, Ben-Hyd, F_2_-Hyd, and GLKFK-Hyd.

### Time evolution of fluorescence and constitution

**Fig. 3:**
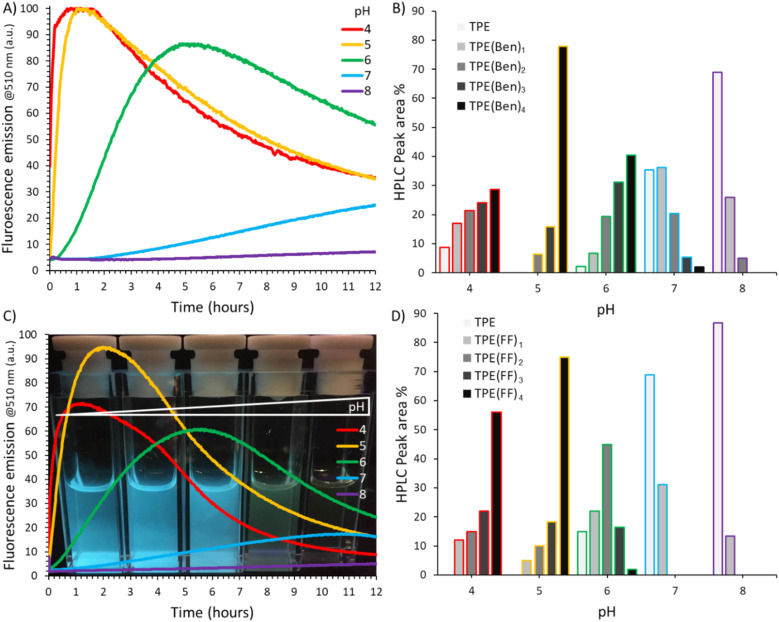
Self-assembly of TPE-Ald (0.3 mM) with BenHyd (top) and F_2_ (bottom): (A) and (C) time evolution of fluorescence emission at different pH with a corresponding photograph taken under UV-light irradiation (*λ*_exc_ = 330 nm); (B) and (D) composition of the systems, determined by LC-MS as peak area %, after 2 hours reaction.

Looking at the evolution of fluorescence emission during the reaction between TPE-Ald (0.03 mM) and BenHyd revealed a rapid turn-on of fluorescence emission within minutes to hours, thus much faster than the disulphide-based systems which usually take days (Fig. 3A).^49–52^ The results confirm the expected pH-dependency of acylhydrazone formation – the fastest (lag-time < 10 min) and most important (*ca.* 20 fold) fluorescence emission enhancements being observed at acidic pH 4–5 while slower (lag-time > 90 min) and much weaker fluorescence emission enhancements (<4-fold) were seen at pH 7–8 (Fig. 3A).^70^ An intermediate situation occurred at pH 6 where a lag time of *ca.* 20 min was observed before the onset of fluorescence emission.

The constitution of the system was then determined by LC-MS analyses, which revealed more pronounced conversion at pH 4-6 – the tetraconjugate TPE(Ben)_4_ being the main product – compared to pH 7–8 where unreacted TPE-Ald remains the main component (Fig. 3B). A very similar trend was seen using F_2_-Hyd (Fig. 3C and D), thus demonstrating the existence of a correlation between the constitution of the system and specifically the extend of acylhydrazone conjugates being formed, and the observed fluorescent emission enhancements that emerge during the self-assembly. The bell-shape pH-dependency can therefore be best explained by the well-known fact that acylhydrazone formation is fastest at mild acidic pH, granting a control over the covalent self-assembly which, in turn, dictates the emergence of the AIE effect. A decrease in the fluorescence emission was observed at long reaction times which is explained by partial precipitation since the HPLC peak area of the tetraconjugate TPE(Ben)_4_ follows the same trend (Fig. S47–49).

**Fig. 4:**
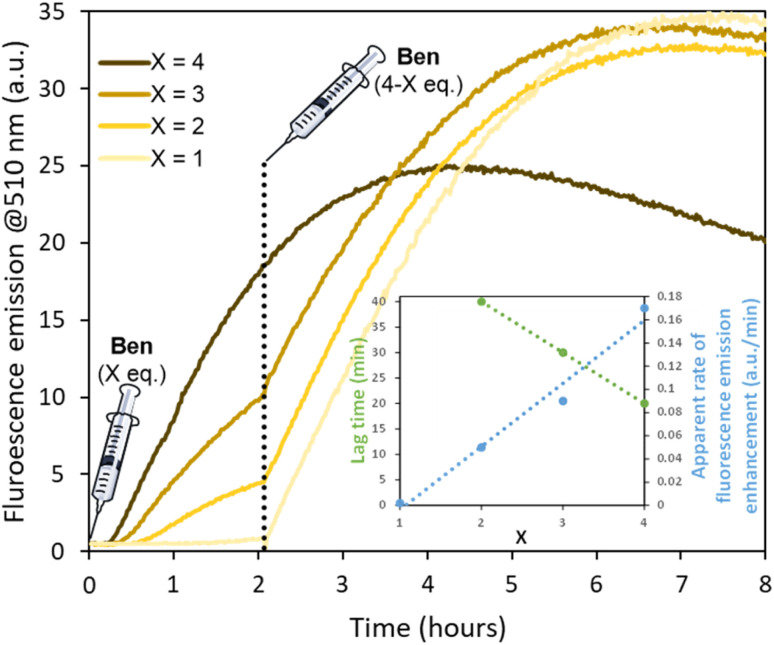
Evolution of fluorescence emission (*λ*_exc_ = 330 nm) during sub-stoichiometric self-assembly of TPE-Ald (0.03 mM) with BenHyd at pH 5 (sodium acetate 100 mM). The stoichiometry was completed to four by a second addition of BenHyd after 2 hours of reaction. The inset represents the lag time and apparent rate of fluorescence emission enhancement in the initial phase for various *X* = 1–4 with a linear fit to guide the eye.

Further support of the intertwining of dynamic covalent self-assembly and supramolecular polymerization was provided by studying sub-stoichiometric conditions. Lowering the concentration of TPE-Ald ten-fold to 0.03 mM reveals a lag time before the onset of fluorescence emission (Fig. 4). Interestingly, this lag time increases as the stoichiometry of BenHyd decreases, reaching a critical situation where no fluorescence turn-on was detected using 1 equiv. of BenHyd (Fig. 4, inset). This is in line with our proposal that it is the first dynamic covalent assembly step of the hierarchical process which determines the subsequent growth of fluorescent aggregates. The rate of fluorescence enhancement appeared also dependent on the stoichiometry, decreasing when lowering stoichiometry (Fig. 4, inset). The subsequent addition, after 2 hours, of BenHyd to make up to a final 4 equiv. stoichiometry immediately resumes, without lag-time, the evolution of the fluorescence emission at its maximum rate (0.13–0.18 a.u./min, Fig. 4). This is most likely the result of a seeding effect where a little amount of aggregates formed in the first stage accelerates the second stage (*vide infra*). A similar result was obtained using F_2_-Hyd (Fig. S50), and demonstrate the chemical control over the fluorescent output through the hierarchical combination of dynamic covalent self-assembly and supramolecular polymerization. The magnitude of the fluorescence emission also depends on the history of the sample, whether the hydrazide partners are added at once or stepwise, which can be due to a partial trapping of aggregates in non-productive forms when 4 equiv. are added straightaway (Fig. 4).

### Emergence of fluorescence by component selection

**Fig. 5:**
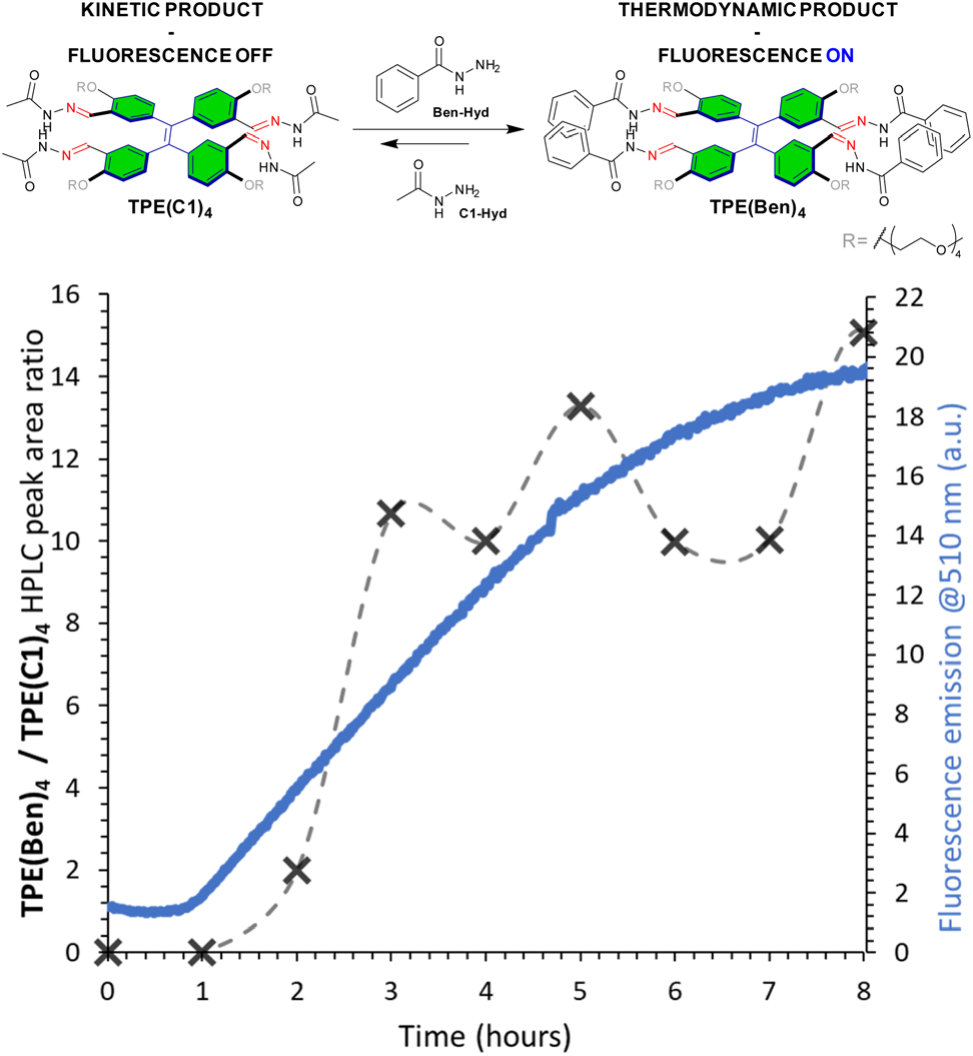
Competition experiment (0.03 mM TPE-Ald, pH 5, sodium acetate 100 mM) between C1-Hyd and BenHyd, monitored by fluorescence spectroscopy (*λ*_exc_ = 330 nm) and LC-MS analysis.

Since the existence of stabilizing secondary interactions translates in the composition of dynamic combinatorial libraries, amplifying the most stable constituent at the expense of the least stable,^71^ we carried out a competition experiment between C1-Hyd and BenHyd, and monitored both the fluorescence emission and the HPLC peak area ratio of tetraconjugates TPE(Ben)_4_/TPE(C1)_4_. At the beginning, a weak fluorescence and a preferential formation of TPE(C1)*_n_* conjugates were observed, most likely due to the more pronounced nucleophilic character of aliphatic (C1-Hyd) *vs.* aromatic (BenHyd) hydrazides (Fig. 5). The situation changed after a lag time of 45 min, when both the TPE(Ben)_4_/TPE(C1)_4_ ratio and the fluorescence emission increased simultaneously (Fig. 5). This observation reveals that the fluorescent tetraconjugate TPE(Ben)_4_ is amplified by component reshuffling and adaptation through reversible covalent self-assembly, leading to the preferential selection of BenHyd. The stepwise component addition further proves the thermodynamic nature of this selection process: no change in fluorescence emission is observed after addition of C1-Hyd onto a solution of TPE(Ben)_4_ while a strong turn-on is observed upon addition of BenHyd onto a solution of TPE(C1)_4_ (Fig. S51). Overall, this result reveals the additional stability provided by the aromatic side group that imposes a component selection – through either stabilization of the monomers or of the supramolecular aggregates – which correlates with the fluorescence emission output of the system.

### The unique case of the pentapeptide hydrazide

**Fig. 6:**
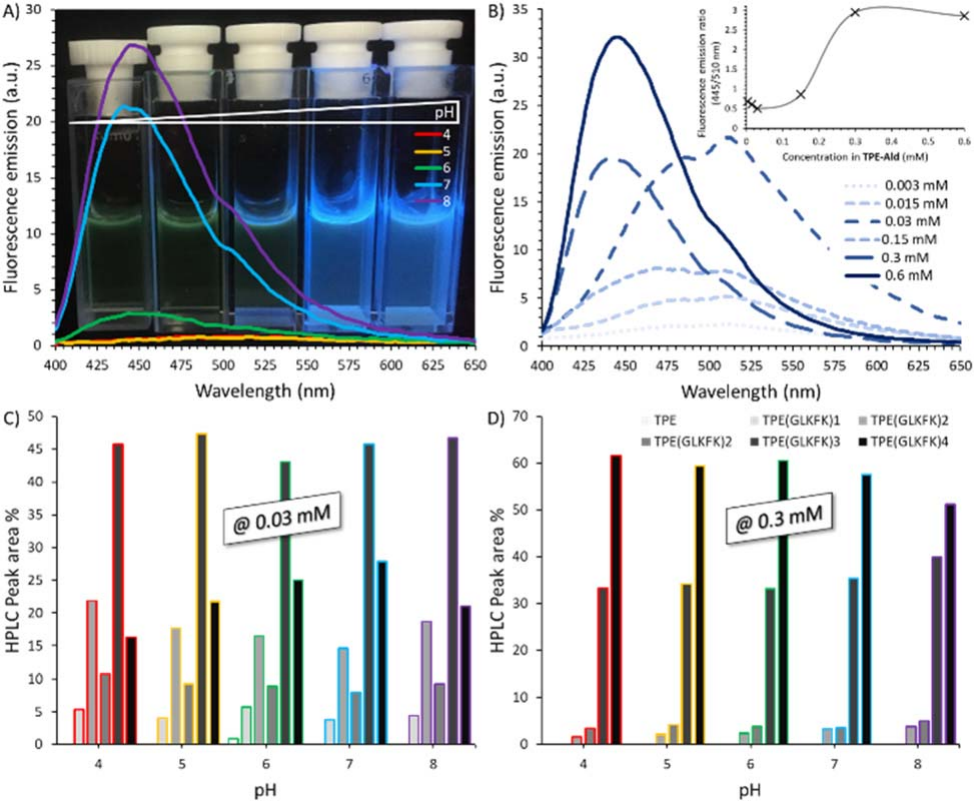
Self-assembly of TPE-Ald with GLKFK-Hyd: (A) fluorescence emission spectra (*λ*_exc_ = 330 nm), after 24 hours reaction (0.3 mM TPE-Ald), at different pH with a corresponding photograph taken under UV-light irradiation; (B) fluorescence emission spectra (*λ*_exc_ = 330 nm), after 24 hours reaction at pH 7, at different concentrations (insets shows fluorescence emission ratio at 445/510 nm as a function of concentration in TPE-Ald); (C) and (D) composition of the systems after 24 hours of reaction at different pH, determined by LC-MS as peak area %, with reactions being carried out at 0.03 and 0.3 mM in TPE-Ald.

During the self-assembly of TPE-Ald with both BenHyd or F_2_-Hyd, we observed a significant blue-shift in the fluorescence emission spectra, the maximum shifting from 510 to 470 nm (Fig. S52 and 53). Such blue-green fluorescence originates from a distortion of conjugation and is typical of columnar TPE aggregates.^27,29,35,72–75^ The self-assembly with the penta-peptide hit GLKFK-Hyd revealed more a pronounced effect. Now, the blue-green fluorescence, peaking now at 445 nm (Fig. S54 and 55), was detected over the whole pH range and was most visible at pH > 6 (Fig. 6A). This blue-shift was found to be strongly concentration-dependent and occurs beyond a threshold concentration of 0.2 mM, thereby showing that it results from an intermolecular self-assembly process (Fig. 6B). The LC-MS analyses of the constitution revealed an excellent conversion (>95%) over the whole pH range, giving a uniform composition made mainly of tris-conjugate TPE(GLKFK)_3_ and tetra-conjugate TPE(GLKFK)_4_ at, respectively, 0.03 and 0.3 mM (Fig. 6C and D).

**Fig. 7:**
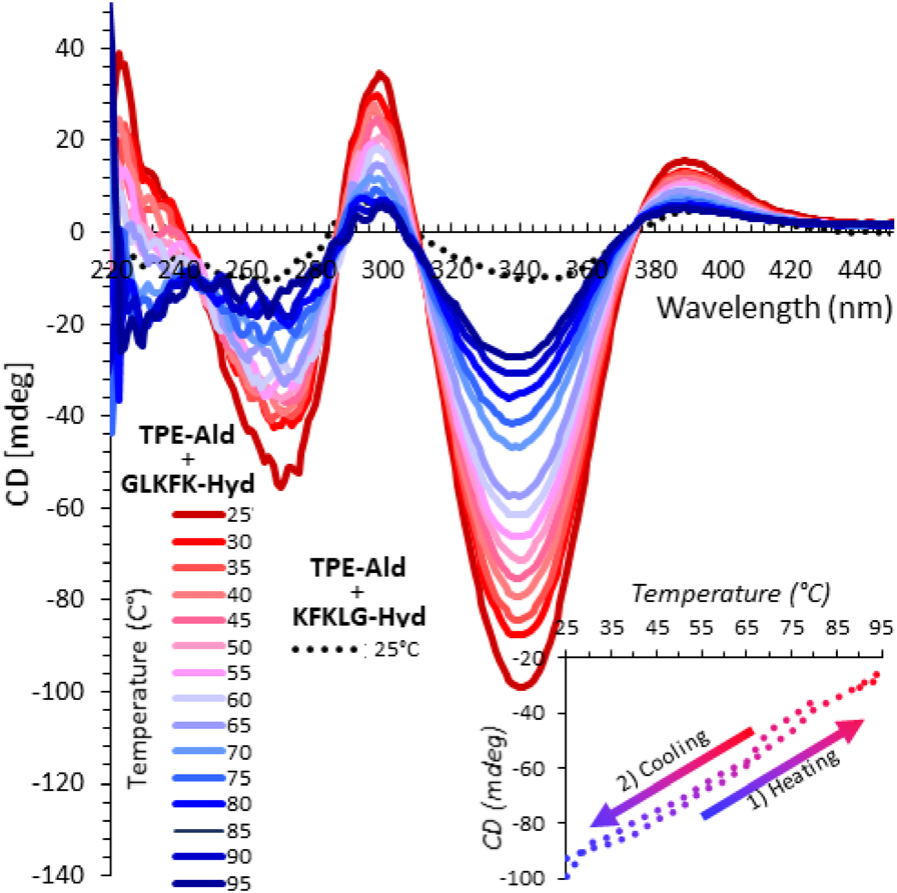
CD spectra at variable temperature of the coupling of TPE-Ald (0.3 mM) with GLKFK-Hyd (full lines) and KFKLG-Hyd (dotted line), carried out at pH 7 (sodium phosphate 100 mM). The reversibility of the process upon a cooling–heating cycle is shown as inset (ellipticity at 340 nm plotted at a function of temperature).

As a further evidence of supramolecular assembly, CD spectroscopy showed an intense signal using GLKFK-Hyd at both pH 5 and 7, whereas a one-order-of-magnitude weaker signal was seen with the reverse peptide KFKLG-Hyd (Fig. 7 and S56). This signal is thermally-reversible which proves again that non-covalent interactions govern the self-assembly process at the origin of the AIE effect and that no Mallory-type photo-cyclization occurs in our conditions using short irradiation times.

**Fig. 8:**
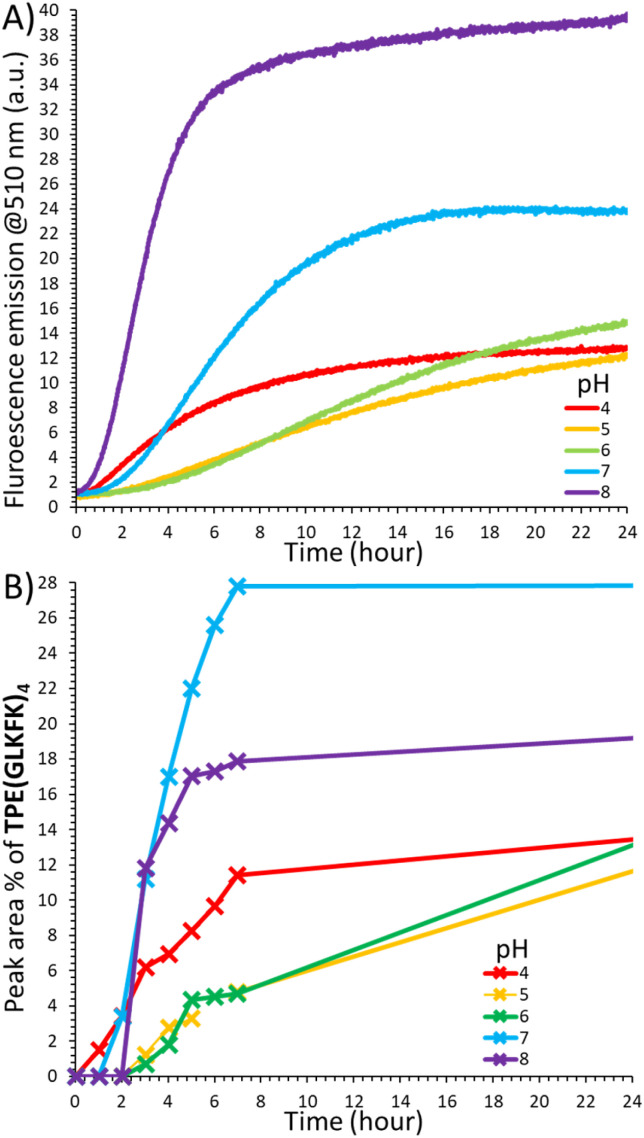
Time evolution of the coupling of TPE-Ald (0.03 mM) with GLKFK-Hyd at different pH: (A) fluorescence emission (*λ*_exc_ = 330 nm), (B) relative proportion of TPE(GLKFK)_4_.

Trying to understand the reason for the acylhydrazone reaction to proceed very well at neutral and basic pH in the presence of the GLKFK-Hyd partner, we monitored, by HPLC, the formation of the tetra-conjugate TPE(GLKFK)_4_ and the evolution of the fluorescence emission (Fig. 8). Conjugation rates were fastest at pH 7–8 with an abrupt onset after a lag-time of 2 hours, which matches the onset of fluorescence emission. The sigmoidal evolution of the fluorescence emission at pH 7–8 is indicative of an auto-catalytic process where the product facilitates its own formation through secondary nucleation.^51,76,77^ This behaviour is in stark contrast with the model self-assembly of benzaldehyde with GLKFK-Hyd, which showed gradual formation of the product facilitated at acidic pH (Fig. S57), and with the self-assembly of TPE-Ald with the reverse peptide KFKLG-Hyd which showed changes more than an order of magnitude weaker (Fig. S58). As a consequence, we excluded a special reactivity of non-assembled GLKFK-Hyd to account for the effective conjugation at pH 7–8 – one may have hypothesized that the neighbouring lysine acts as internal organo-catalyst.^78,79^ Instead, we propose that the aggregate of TPE(GLKFK)_4_ accelerates the rate-limiting acylhydrazone formation, of TPE-Ald and GLKFK-Hyd, leading, through a feed-back loop (Fig. 1), to the phenomenon of auto-catalysis.^80^ This could be due to a local concentration effect where the supramolecular assemblies bind aldehyde and hydrazide reactants^81^ or due to a catalytic effect of the amines in the assembly, or a combination of both. Most likely, this phenomenon is favoured at neutral pH because the peptide is less protonated and therefore more prone to engage in substrate binding and organocatalysis, and the acceleration of the covalent coupling most detectable because of a very slow background reaction at pH 7–8 where the initial formation of TPE(GLKFK)_4_ is the slowest. Support for this hypothesis comes from the independent and parallel work of Otto that shows enzyme-like catalysis of acylhydrazone formation at near-neutral pH promoted by a different type of supramolecular self-replicator featuring the same GLKFK peptide sequence.^82^ Also, in that system catalysis of acylhydrazone formation is an emergent property of the assembly.

### Nanoscale characterization

**Fig. 9:**
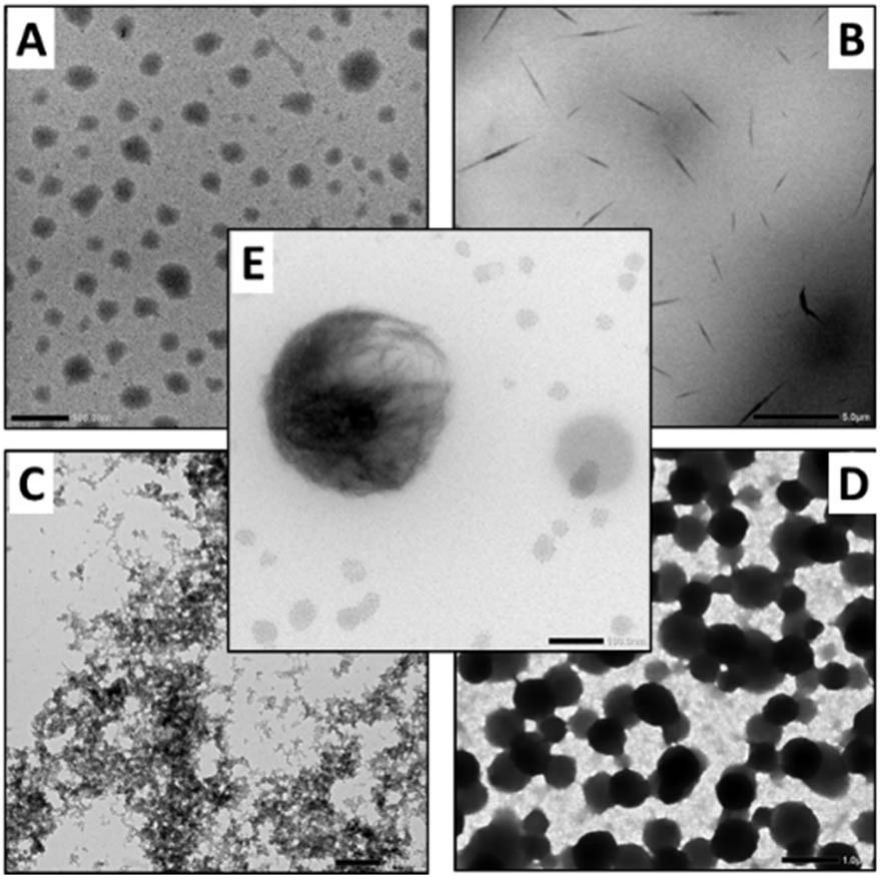
TEM images of the self-assembly of TPE-Ald with (A) C1-Hyd (0.3 mM), scale bar 500 nm; (B) C6-Hyd (0.03 mM), scale bar 5 μm; (C) BenHyd (0.3 mM), scale bar 200 nm; (D) GLKFK-Hyd (0.03 mM, pH = 5, sodium acetate 100 mM), scale bar 1 μm; (E) F_2_-Hyd (0.3 mM, pH = 5, sodium acetate 100 mM), scale bar 100 nm.

TEM analyses showed nanoparticles formation with C1-Hyd (diameter ≈ 200 nm, Fig. 9A), high aspect ratio fibrils with C6-Hyd (1–5 μm length, 100–200 nm width, Fig. 9B), intertwined bead-on-string fibrils with BenHyd (Fig. 9C), and dense spherical nanoparticles with GLKFK-Hyd (diameter ≈ 500–800 nm, Fig. 9D). While the concentration-independent, light intensity, and regular circularity of the nanoparticles observed with C1-Hyd suggest an assembly induced during sample preparation and the slow solvent evaporation, the other cases reflect nano-assemblies formed in solution with sizes greatly exceeding supramolecular dimers or oligomers. The formation of a ball-of-wool like nanoassembly with F_2_-Hyd indicate that 1D fibrillar supramolecular polymers do form, followed by a further 2D and 3D growth process (Fig. 9E). In the end, it appears clear that the nature of the hydrazide has a profound impact on the nanoscale architecture, affecting persistence length and dimensionality as reported by others on different systems.^28^ Although more investigations will be needed to understand and master this bottom-up self-assembly process,^83^ it confirms that the dynamic covalent assembly dictates not only the aggregation pathway but also the nature of the hierarchical assembly and the dimensionality of the nanostructures formed.

## Conclusion

We reported here the *in situ* hierarchical self-assembly of fluorescent supramolecular aggregates in aqueous medium. The implementation of a combinatorial screening assay enabled the rapid identification of conditions and partners favouring the formation of fluorescent assemblies. The dynamic covalent self-assembly of water-soluble TPE-Ald, with complementary hydrazide partners, followed by the subsequent supramolecular polymerization, were found to be deeply intertwined: the control over the molecular assembly by stoichiometry, concentration, pH, and choice of the hydrazide partner dictates the emergence of the fluorescent supramolecular polymers, while the formation of the latter imposes selection preferences of the most suited building block and lead to its auto-catalytic amplification at neutral pH where acylhydrazone formation is otherwise usually very slow. This work provides an example of a complex chemical system^84–86^ which displays a fine constitution–function relationship, and matches the growing interest for controlling the emergence of functional supramolecular polymers^87^ and nano-structures^88,89^ through chemical inputs. Such system hierarchically self-assembling at physiological pH has the potential to be applied in living organisms, as recently exemplified using non-fluorogenic supramolecular polymers also based on the chemistry of acylhydrazones.^90^

## Author contributions

MC: investigation, resources and visualization; SU: supervision/validation and conceptualization/methodology/funding acquisition/project administration. Both authors contributed to the writing, reviewing, and editing.

## Conflicts of interest

There are no conflicts to declare.

## Supplementary Material

SC-OLF-D5SC04688B-s001

## Data Availability

All our author-generated datasets are directly embedded in the manuscript and data are provided in the supporting information file (SI). Supplementary information: synthesis and characterization of molecular building blocks, model conjugations and complementary data on the screening and monitoring of the self-assembly. See DOI: https://doi.org/10.1039/d5sc04688b.
