# Supplementary material for: Emergence of fluorescent aggregates through hierarchical self-assembly
Source: Chem Sci. 2025 Oct 16;16(47):22438–46. doi: 10.1039/d5sc04688b (PMC12550600; doi:10.1039/d5sc04688b)
Supplement: SC-016-D5SC04688B-s001 [file SC-016-D5SC04688B-s001.pdf]

Supplementary Information (SI) for Chemical Science.
This journal is © The Royal Society of Chemistry 2025

SUPPORTING INFORMATION

1. Materials and methods 2
2. General procedures 3
3. Synthesis of TPE-Ald 4
4. Synthesis of hydrazides 6
5. Conjugation reaction with C1-Hyd 30
6. Screening assays of aliphatic and gallic hydrazide derivatives 31
7. Self-assembly of Ben-Hyd and TPE-Ald 31
8. Time evolution of the self-assembly of Ben-Hyd and TPE-Ald 32
9. Time evolution of the self-assembly of F_2-Hyd and TPE-Ald 33
10. Competition experiment between C1-Hyd and BenHyd 33
11. Spectral evolution in the self-assembly of Ben-Hyd and TPE-Ald 34
12. Spectral evolution in the self-assembly of F_2-Hyd and TPE-Ald 34
13. Self-assembly of GLKFK-Hyd and TPE-Ald 35
14. CD spectroscopy 36
15. Self-assembly of GLKFK-Hyd and Benzaldehyde 36
16. Self-assembly of KFKLG-Hyd and TPE-Ald 37
17. References 38

1. Materials and methods

All reagents and solvents were obtained from commercial sources and were used without further purifications.

Nuclear magnetic resonance spectroscopy (NMR). ^{1}H NMR spectra were recorded at 400 MHz (Bruker Avance 400 instruments) in deuterated solvents. Peaks were referenced in ppm with respect to the residual solvent peak. Data are reported as follows: chemical shift (δ in ppm), multiplicity (s for singlet, d for doublet, t for triplet, m for multiplet, br for broad signal), coupling constant (*J* in Hertz), and integration. Spectra were recorded at 25°C.

High-performance liquid chromatography (HPLC). Analytical reverse-phase HPLC (RP-HPLC) analyses were performed on a Waters HPLC 2695 (EC Nucleosil 300-5 C18, (125 x 3 mm) column, Macherey-Nagel) equipped with a Waters 996 DAD detector with the following linear gradients of solvent B (acetonitrile 100%) into solvent A (TFA 95% and acetonitrile 5%): Method: 0 to 95% of solvent B in 5 min; flow: 1 mL/min. Retention times (t_R) are given in minutes. Preparative HPLC was performed on a VWR International LaPrep pump P110, a VWR LaPrep P314 Dual l absorbance detector and EZChrom software (15 C_{18} reversed-phase column Waters x-bridge, RP-18, 25 ´ 250 mm, 5 µm), flow 40 min/mL, using a binary gradient elution. HPLC eluents: (Solution A: 99.9% Water, 0.1% TFA; Solution B: 99.9% Acetonitrile, 0.1% TFA).

Liquid chromatography-mass spectrometry (LC/MS). Analyses were performed on a Shimadzu LCMS2020 (Phenomex Kenetex C18, 2.6 µm ^{x} 7.5 cm, 100Å) equipped with a SPD-M20A detector with the following linear gradient of solvent B (99.9% acetonitrile, 0.1% HCOOH) and solvent A (99.9% water and 0.1% HCOOH): 5 to 95% of solvent B in 5 min; flow 1 ml/min. Retention times (t_R) are given in minutes.

Mass spectrometry (MS). Electrospray ionization (ESI-MS) analyses were carried out at the Laboratoire de Mesures Physiques, IBMM, Université de Montpellier using Micromass Q-Tof instruments (positive mode).

Flash column chromatography. Flash column chromatography was carried out on a puriflash 430 instrument (Interchim) using 30 µm spherical silica or 40-60 µm irregular silica cartridges.

Fluorescence spectroscopy. Fluorescence analyses were carried out on a Safas Xenius spectrofluorometer. The spectra were recorded at 20°C between 450 and 650 nm with an excitation wavelength at 330 nm and a bandwidth of 1 nm. Kinetics were recorded at 25°C at 470 nm and 510 nm.

Circular dichroism spectroscopy (CD). Circular dichroism measurements were recorded using a JASCO J-815 CD Spectrophotometer at IBMM, Université de Montpellier.

Transmission Electronic Microscopy (TEM). TEM assays were performed on a JEOL 1200 EXII 120 kV instrument at the plateforme de Microscopie électronique et analytique (MEA),

Université Montpellier. The compounds were fixed on a full grid of carbon (300 mesh). The samples were then placed under electron flux. The resolution of this technique allows the observation of systems measuring more than 1 nm. TEM analyses were realized at 0.003, 0.03 and 0.3 mM. Compounds were mixed in buffer, agitated and 5 µL of the reaction mixture were immediately put on the full grid of carbon. The grids were analysed after 12 hours.

2. General procedures

Procedures for the screening assays and in situ dynamic covalent assembly.
For all experiments, the buffers used were: 100 mM sodium acetate for pH 4, 5, and 6; and 100 mM sodium phosphate for pH 7 and 8.
Preparation of samples. [10 mM] stock solution of **TPE-Ald** in water. [40 mM] stock solutions of hydrazides were prepared in: a) DMSO for **C1-Hyd** to **C7-Hyd, Piv-Hyd**, **Ben-Hyd**, **Gal(Cn')-Hyd**, **F_n-Hyd** and **W_n-Hyd**; b) Water for **Gal(EG)-Hyd, A_n-Hyd** and all the ionic peptide hydrazides.

Screening of aliphatic and gallic hydrazides. Screenings were carried out using white 96-well plates with a final volume of 200 µL at 0.3 mM concentration of **TPE-Ald**. 6 µL of **TPE-Ald** stock solution [10 mM] were placed and 188 µL of buffer with 5% of DMSO were then added. For each line, a different pH was used, from pH 4 to pH 8. Fluorescence emission was assessed at λ_{exc} = 330 nm, λ_{em} = 510 nm. Next, 6 µL of hydrazide stock solution [40 mM] were added and the plate was read overtime from 0 to 24 hours.

Screening of C-hydrazide peptides. Screenings were carried out using white 96-well plates with a final volume of 200 µL at 0.3 mM concentration of **TPE-Ald**. 6 µL of **TPE-Ald** stock solution [10 mM] were placed and 188 µL of buffer were then added. For each line, a different pH was used, from pH 4 to pH 8. Fluorescence emission was assessed at λ_{exc} = 330 nm, λ_{em} = 510 nm. Next, 6 µL of hydrazide stock solution [10 mM] were added and the plate was read overtime from 0 to 24 hours.

3. Synthesis of TPE-Ald

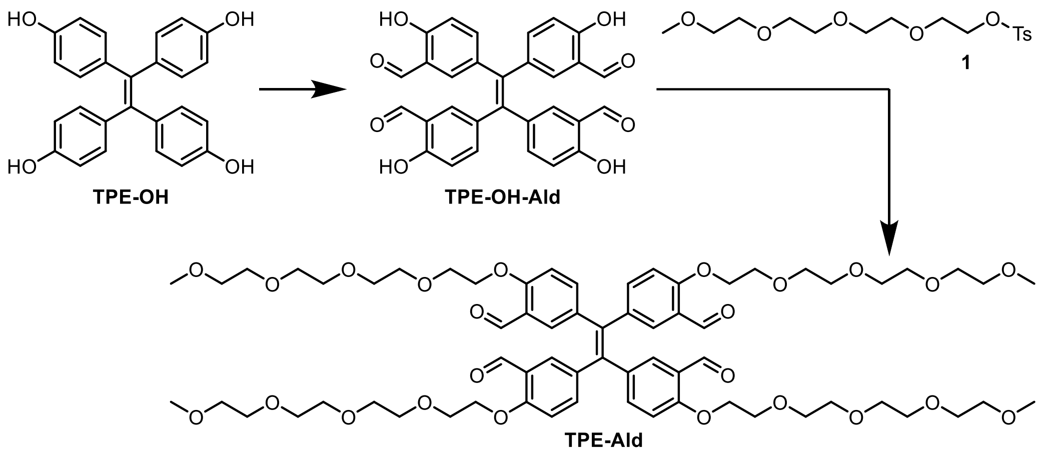

Scheme S1: Synthetic route for the preparation of **TPE-Ald**. Ts: Tosyl.

TPE-OH,[1] **TPE-OH-Ald**,[2] and compound **1**[3] were synthesized following previously reported protocols.

TPE-Ald. Compound **1** (4.8 eq.) and $CsCO_3$ (8 eq.) were dissolved in dry DMF to a final concentration of 80 mM]. KI (1 eq.) was added. After 10 min, the compound **TPE-OH-ALD** (28 mg, 1 eq.) was added and the reaction mixture was stirred under argon at reflux, overnight. The reaction mixture was then concentrated and dried in *vacuo*. 70 mg (50%) of the desired **TPE-Ald** were obtained after reverse-phase HPLC purification (5% B for 5 min, then → 65% B in 30 min). ^{1}H NMR (400 MHz, CD_3CN): δ = 10.33 (s, 4H, H1), 7.41 – 7.40 (dd, 4H, J = 1.2, 2.4, H_6), 7.13 (dd, 4H, J = 1.2, 2.4 H_3), 6.71 (d, J = 8.7, 4H, H_7), 4.16 (t, J = 4.2, 8H, H_9), 3.87 (t, J = 4.2, 8H, H_{10}), 3.69 – 3.68 (m, 8H, H_{11}-H_{16}), 3.66 – 3.61 (m, 32H, H_{11}-H_{16}), 3.54 – 3.51 (m, 8H, H_{11}-H_{16}), 3.36 – 3.35 (m, 12H, H_{17}); LC-MS: t_R 4.13 min; MS calcd for m/z $[C_{66}H_{92}O_{24}+H]^+$ 1269.61, found 1269.40, $[C_{66}H_{92}O_{24}+NH_4]^+$ 1286.63, found 1286.50, $[C_{66}H_{92}O_{24}+Na]^+$ 1291.58, found 1291.40, $[C_{66}H_{92}O_{24}+2H]^{2+}$ 635.30, found 635.55; $[C_{66}H_{92}O_{24}+H+NH_4]^{2+}$ 643.82, found 644.05, $[C_{66}H_{92}O_{24}+2H]^{2+}$ 635.30, found 635.55; HR-ESI-MS calcd for m/z $[C_{66}H_{92}O_{24}+H]^+$1269.6057, found 1269.6050, $[C_{66}H_{92}O_{24}+2H]^{2+}$ 635.3062, found 635.3050.

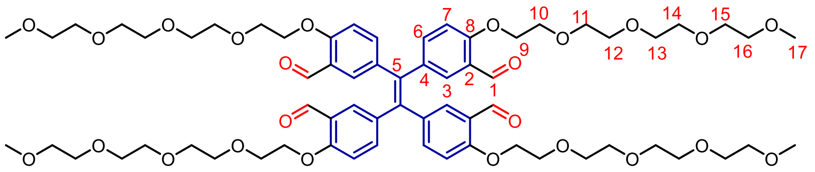

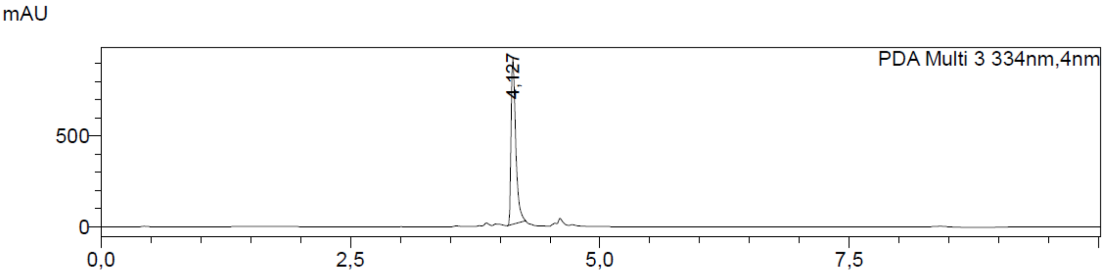

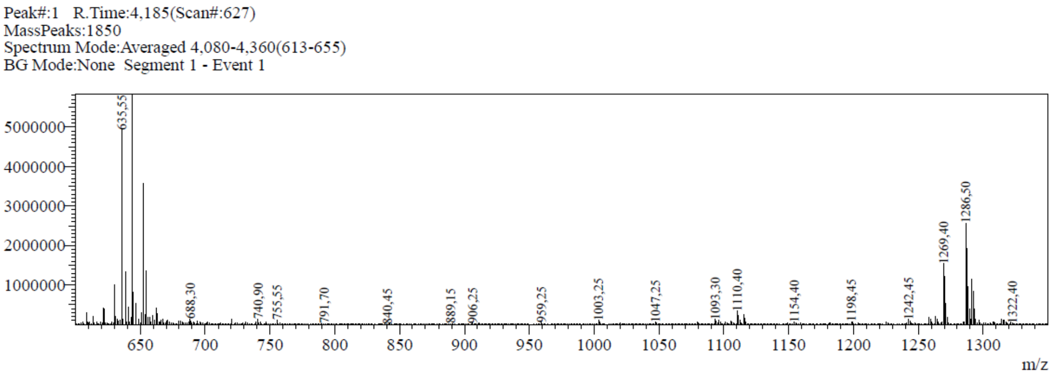

Figure S2: LC/MS analysis: HPLC chromatogram (top), and ESI-MS spectrum (bottom).

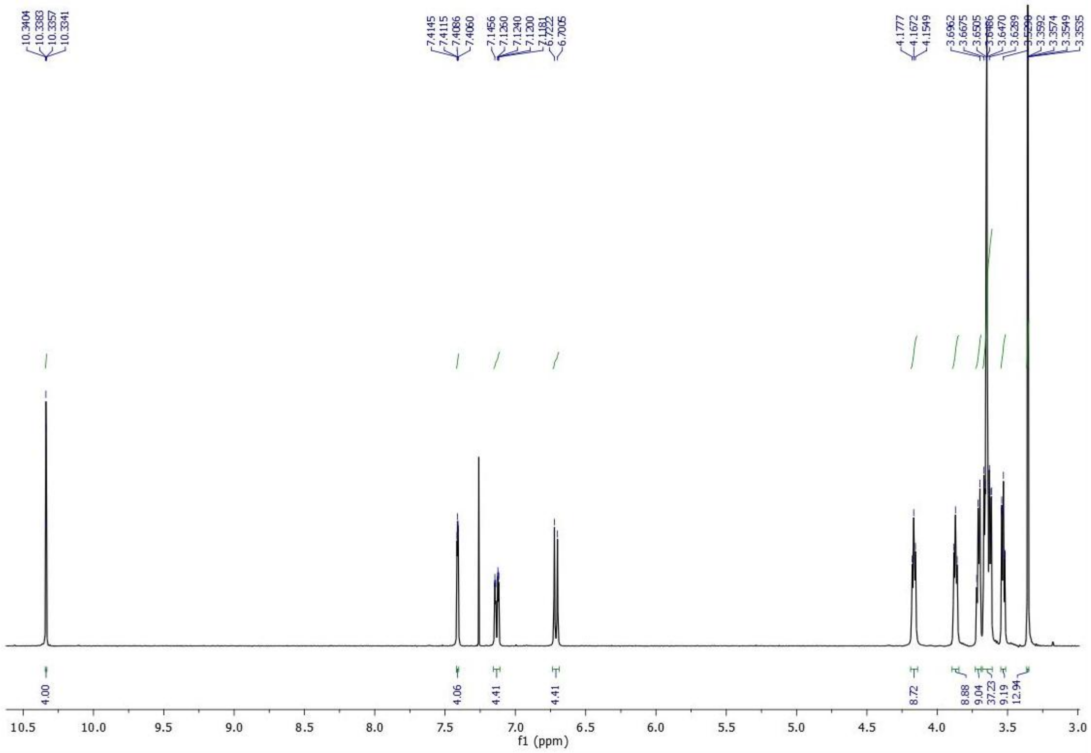

Figure S2: ^{1}H NMR (400 MHz, CD_3CN) spectrum of **TPE-Ald**.

4. Synthesis of hydrazides

Gallic acid derivatives **Gal(EG)**, **Gal(Cn')**, and gallic hydrazide derivatives **Gal(EG)-Hyd**, **Gal(Cn')-Hyd** (C4, C5, C6, C8, C14) were synthesized following reported procedures.[4-10]

The C-hydrazide peptides were synthesized through solid-phase peptide synthesis (SPPS) using a Fmoc strategy. SPPS was carried out manually on modified Fmoc-hydrazine resin, as previously reported.[11-13] The final peptides were all titrated by ^{1}H NMR using *tert*-butanol as internal reference in order to determine the exact concentration.

A_1-Hyd. The modified amino acid was synthesized using Fmoc-*L*-Ala-OH. The crude product was obtained after precipitation in Et_2O and freeze drying. The compound **A_1-Hyd** was isolated

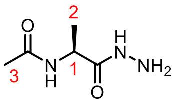

by preparative HPLC purification (0% B for 15 min, then → 20% B in 15 min). Yield: 35 %. ^{1}H NMR (400 MHz, D_2O): δ = 4.20 (q, *J* = 7.2, 1H, H_1), 2.09 (s, 3H, H_3), 1.60 (d, *J* = 7.2, H_2); LC-MS: t_R 0.42 min; ESI-MS calcd for m/z [2($C_5H_{11}N_3O_2$)+H]$^+$ 291.09, found 291.00.

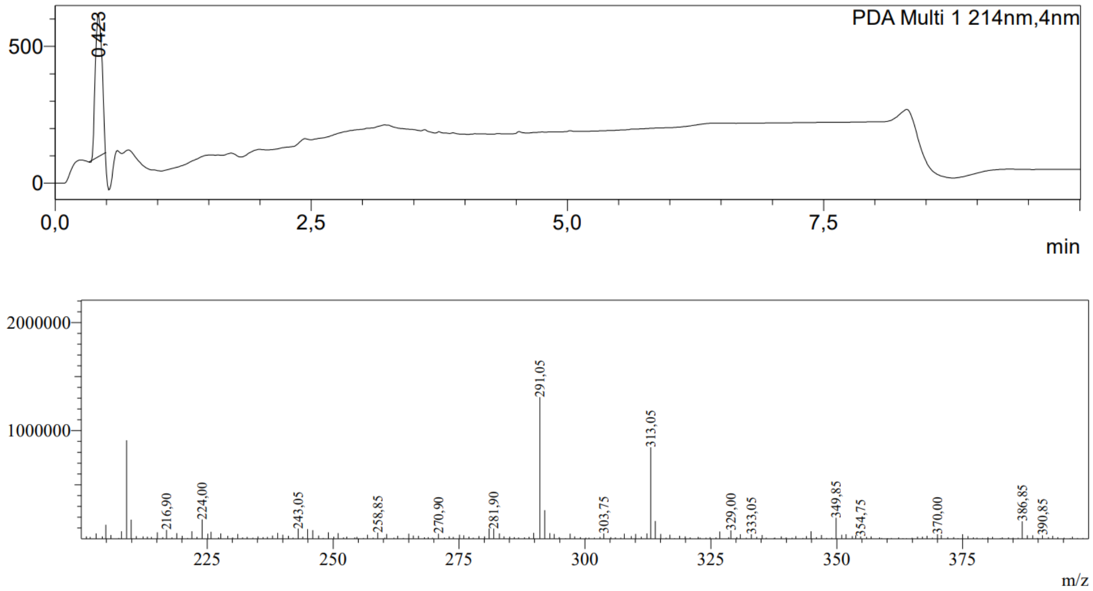

Figure S3: LC/MS analysis: HPLC chromatogram (top) and ESI-MS spectrum (bottom).

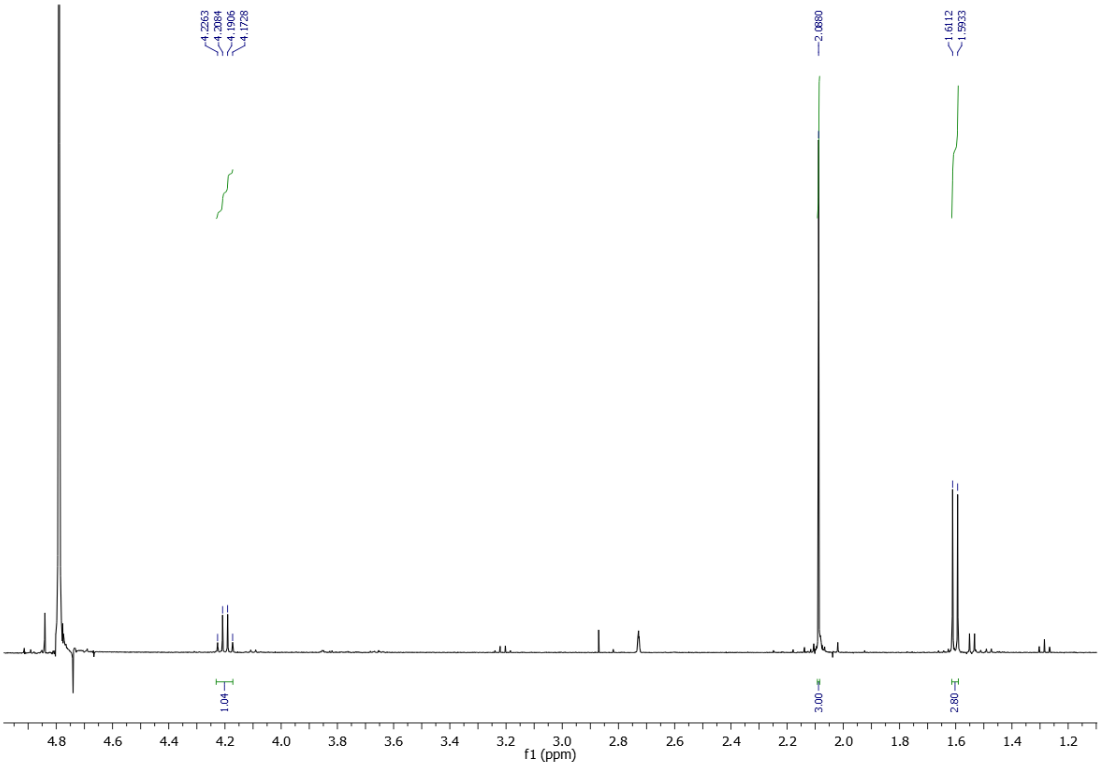

Figure S4: ^{1}H NMR (400 MHz, D_2O) spectrum of **A_1-Hyd**.

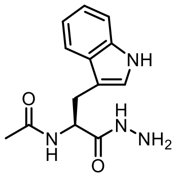

W_1-Hyd. The modified amino acid was synthesized using Fmoc-*L*-Trp(Boc)-OH. The crude product was obtained after precipitation in Et_2O and freeze drying. The compound **W_1-Hyd** was isolated by preparative HPLC purification (0% B for 5 min, then → 50% B in 50 min). Yield: 25 %. ^{1}H NMR (400 MHz, CD_3CN/D_2O 1/1): 8.15 – 8.13 (m, 1H), 7.99 – 7.94 (m, 1H), 7.79 – 7.71 (m, 3H), 4.73 (s, 1H, H_α), 3.89 – 3.83 (m, 2H, H_β), 2.52 (s, 3H, CH_3); LC/MS: t_R 1.04 min; LC/MS calcd for m/z $[C_{13}H_{16}N_4O_2+H]^+$ 261.13, found 261.00, $[2(C_{13}H_{16}N_4O_2)+H]^+$ 521.26, found 521.05.

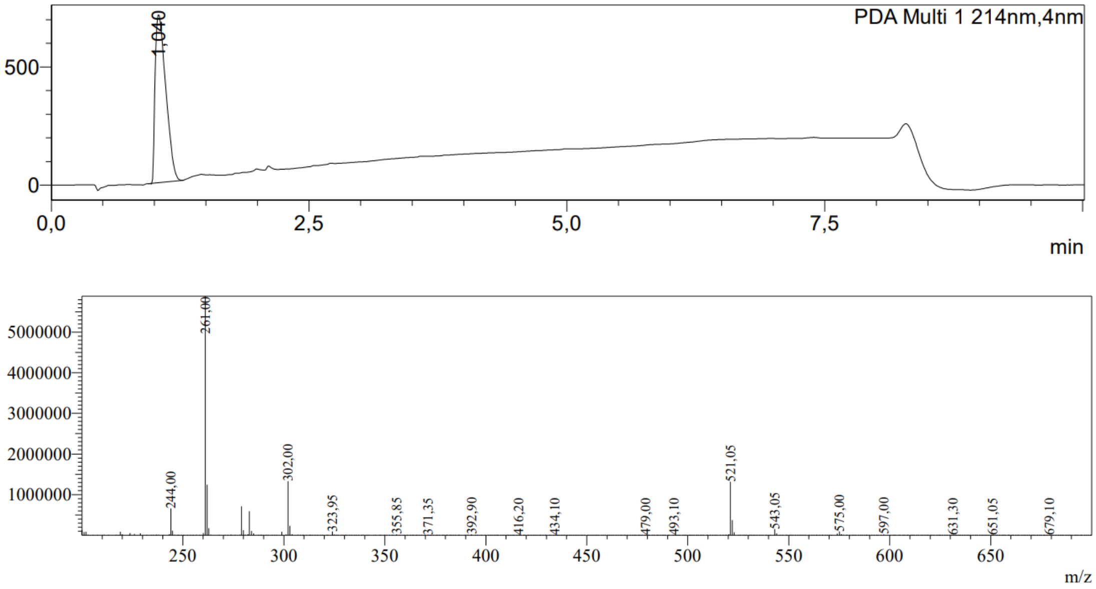

Figure S5: LC/MS analysis: HPLC chromatogram (top) and ESI-MS spectrum (bottom).

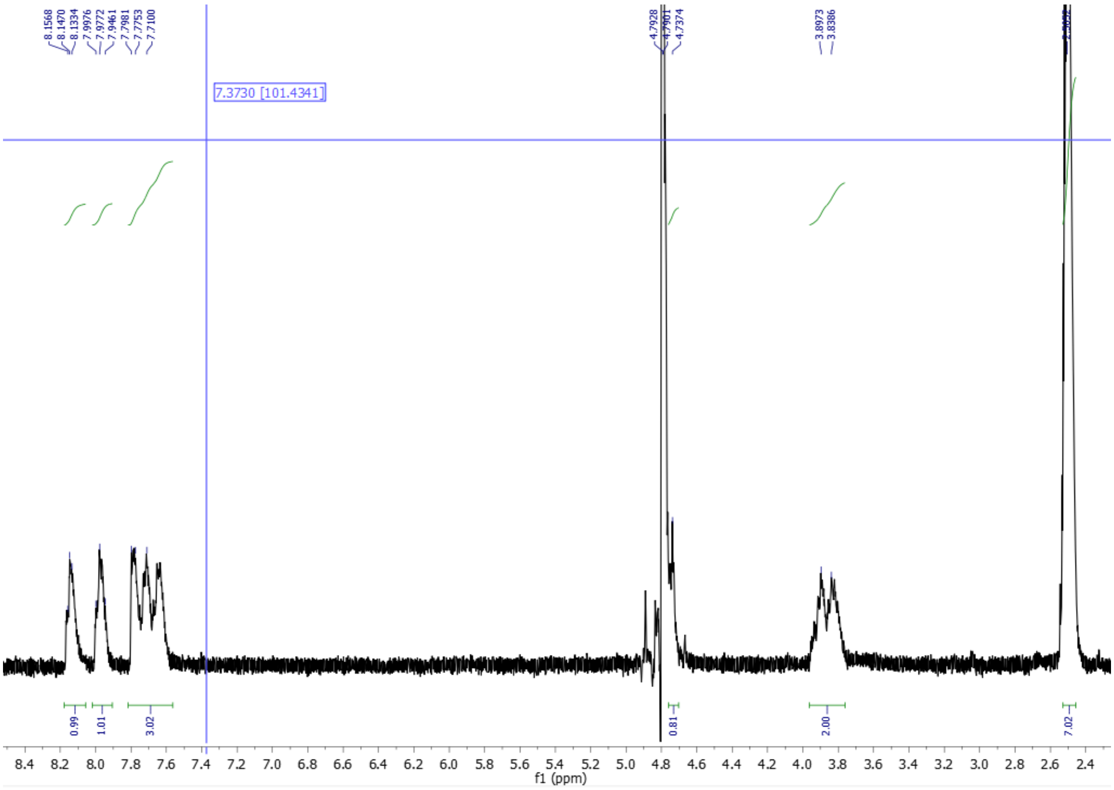

Figure S6: ^{1}H NMR (400 MHz, CD_3CN/D_2O 1/1) spectrum of **W_1-Hyd.**

F_1-Hyd. The modified amino acid was synthesized using Fmoc-*L*-Phe-OH. The crude product was obtained after precipitation in Et_2O and freeze drying. The compound **F_1-Hyd** was isolated by preparative HPLC purification (0% B for 5 min, then → 50% B in 50 min). Yield: 33 %. ^{1}H NMR (400 MHz, MeOD): 7.31 – 7.21 (m, 4H), 4.61 – 4.57 (m, 1H, H_{α}), 3.15 – 2.91 (ddd, 2H, CH_2), 2.02 (s, *t*BuOH) 1.91 (s, 3H, CH_3); LC/MS: t_R 2.18 min; ESI-MS calcd for m/z $[C_{11}H_{15}N_3O_2+H]^+$ 222.12, found 222.00; $[2(C_{11}H_{15}N_3O_2)+H]^+$ 443.24, found 443.15.

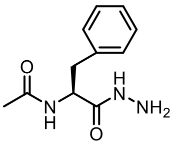

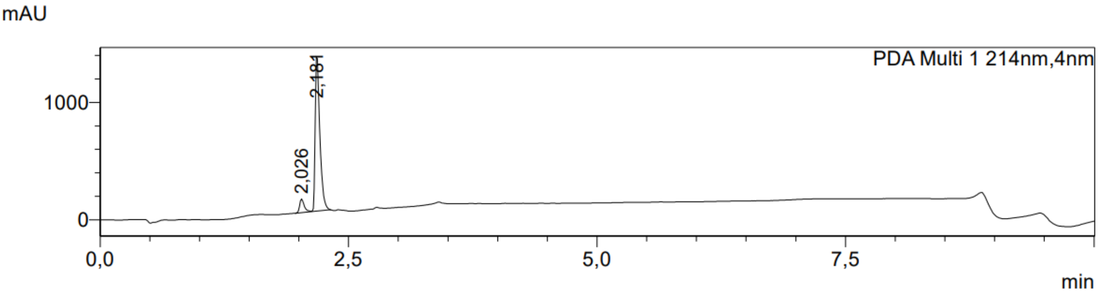

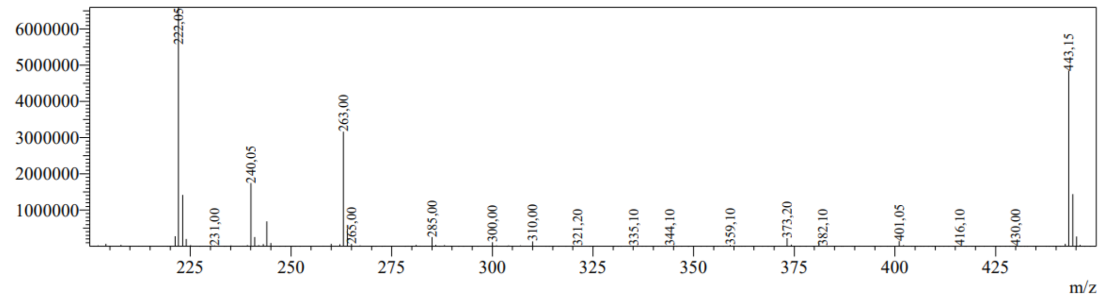

Figure S7: LC/MS analysis: HPLC chromatogram (top) and ESI-MS spectrum (bottom).

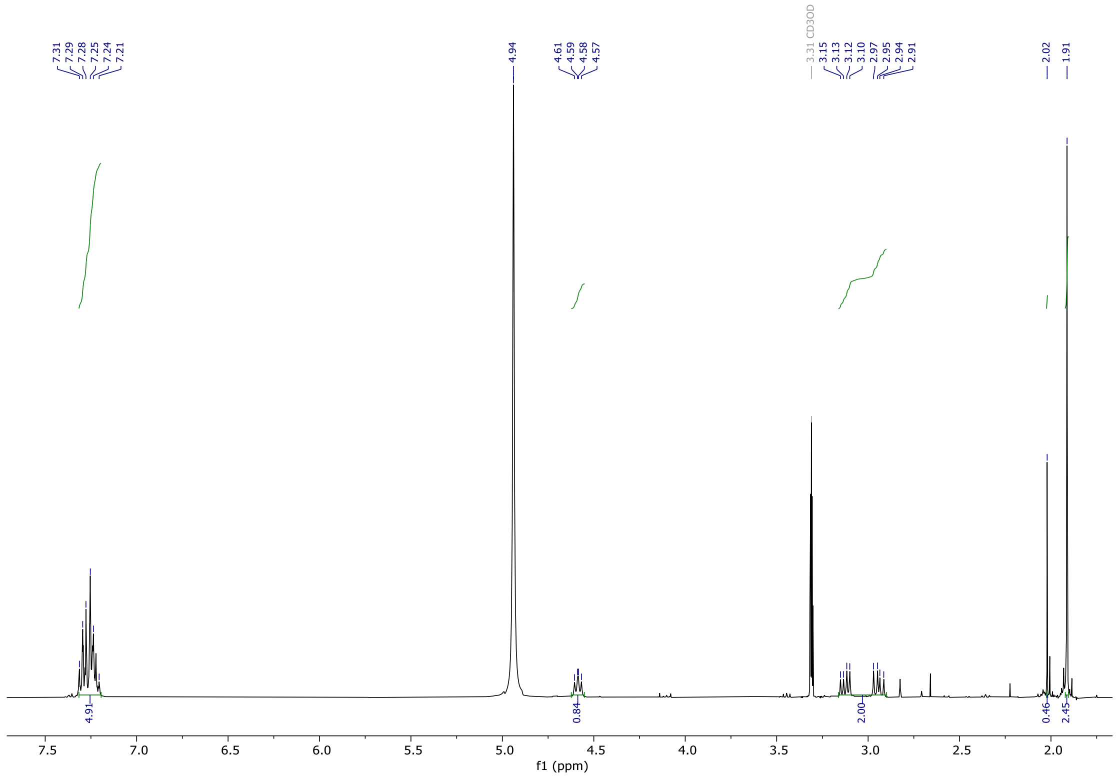

Figure S8: ^{1}H NMR (400 MHz, MeOD) spectrum of **F$_1$-Hyd.**

A$_2$-Hyd. The modified amino acid was synthesized using Fmoc-*L*-Ala-OH. The crude product was obtained after precipitation in Et$_2$O and freeze drying. The compound **A$_2$-Hyd** was isolated by preparative HPLC purification (0% B for 15 min, then → 20% B in 15 min). Yield: 56 %. ^{1}H NMR (400 MHz, D$_2$O): δ = 4.45 – 4.25 (m, 2H, H$_1$, H$_2$), 2.03 (s, 3H, H$_5$), 1.45 – 1.36 (m, 6H, H$_3$, H$_4$); LC/MS: t_R 0.53 min; ESI-MS calcd for m/z [$C_8H_{16}N_4O_3$+H]$^+$ 217.12, found 217.05.

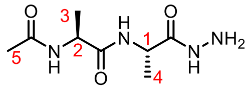

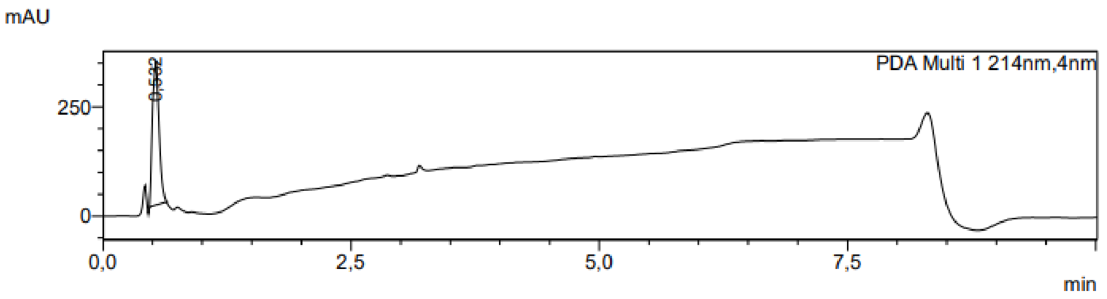

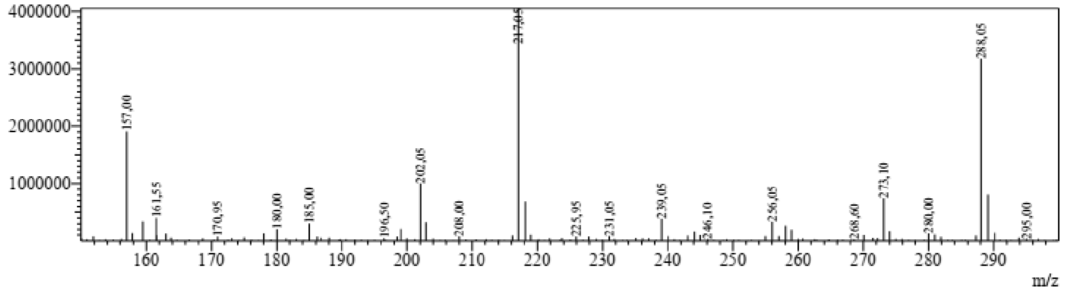

Figure S9: LC/MS analysis: HPLC chromatogram (top) and ESI-MS spectrum (bottom).

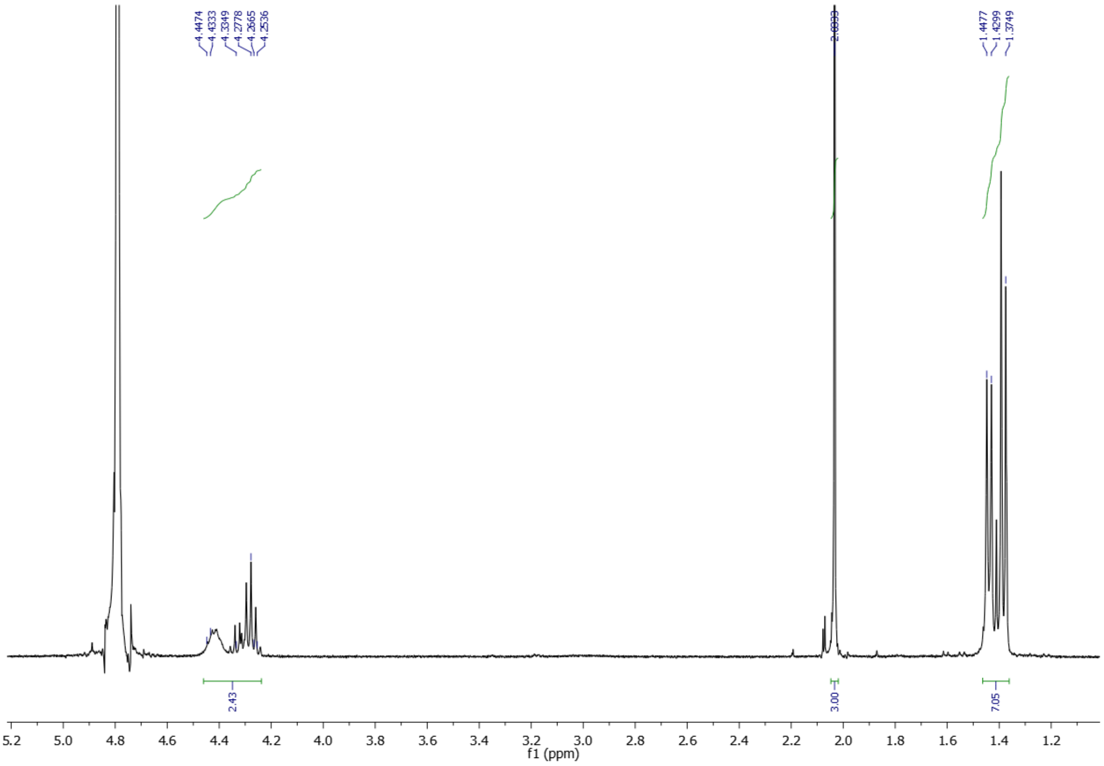

Figure S10: ^1H NMR (400 MHz, D_2O) spectrum of **A_2-Hyd.**

W_2-Hyd. W_2-Hyd was synthesized according the general SPPS procedure using Fmoc-*L*-Trp(Boc)-OH. The desired product was isolated by preparative HPLC purification (0% B for 5 min, then → 60% B in 50 min). Yield: 42 %. ^1H NMR (400 MHz, CD_3CN/D_2O 1/1): 7.99 – 7.93 (m, 3H), 7.74 – 7.31 (m, 7H), 5.10 (br, 1H, $H_2/H_{2'}$), 4.91 (br, 1H, $H_2/H_{2'}$), 3.56 – 3.50 (m, 4H, H_3, $H_{3'}$), 2.50 (s, 3H, H_1); LC/MS: t_R 2.40 min; ESI-MS calcd for m/z $[C_{24}H_{26}N_6O_3+H]^+$ 447.21, found 447.15; $[2(C_{24}H_{26}N_6O_3)+H]^+$ 893.42, found 893.15.

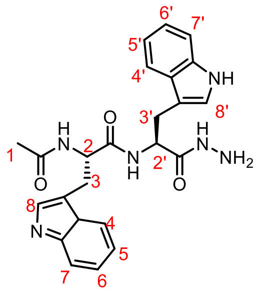

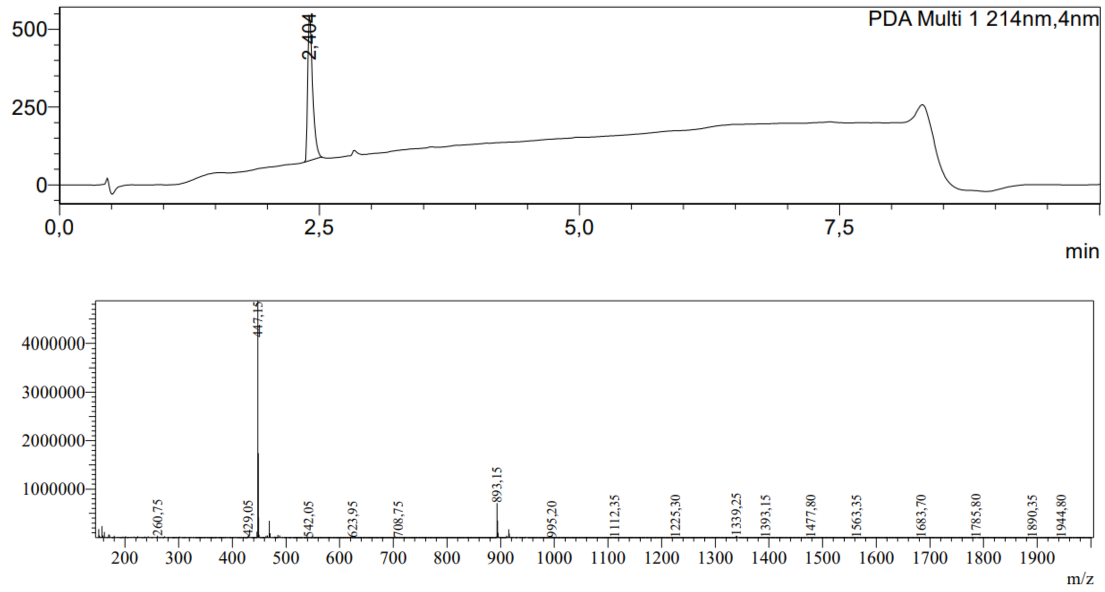

Figure S11: LC/MS analysis: HPLC chromatogram (top) and ESI-MS spectrum (bottom).

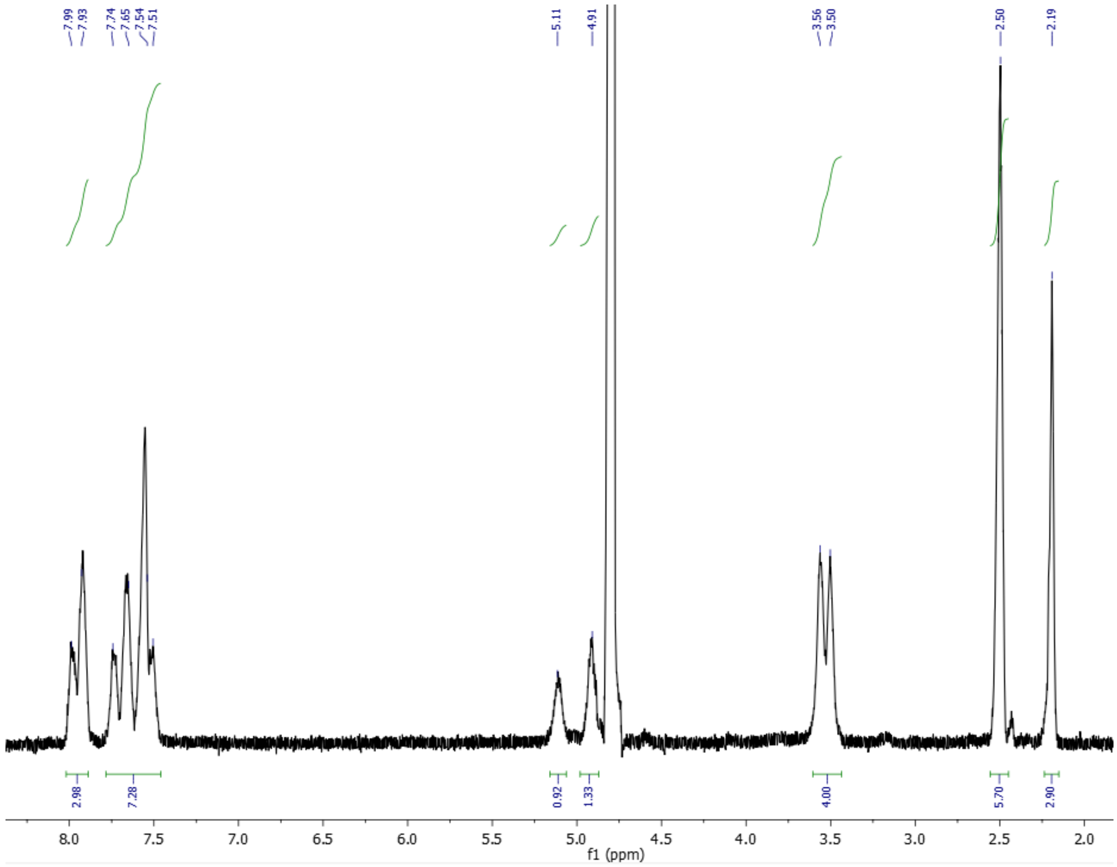

Figure S12: ^{1}H NMR (400 MHz, CD_3CN/D_2O 1/1) spectrum of **W_2-Hyd**.

F_2-Hyd. The modified amino acid was synthesized using Fmoc-*L*-Phe-OH. The crude product was obtained after precipitation in Et_2O and freeze drying. The compound **F_2-Hyd** was isolated by preparative HPLC purification (10% B for 5 min, then → 60% B in 50 min). Yield: 52 %. ^{1}H NMR (400 MHz, CD_3CN/D_2O 1/1): 7.76 – 7.63 (m, 8H), 7.53 (m, 2H), 3.55 – 3.12 (m, 1H, CHα), 3.34 – 3.28 (m, 1H, CHα), 2.42 – 2.40 (m, 4H, CH_2), 2.26 (s, 3H, CH_3); LC/MS: t_R 3.29 min; ESI-MS m/z calcd for $[C_{20}H_{24}N_4O_3+H]^+$ 369.18, found 369.10; $[C_{20}H_{24}N_4O_3+H]^+$ 737.36, found 737.50.

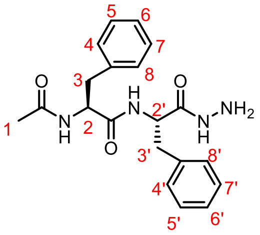

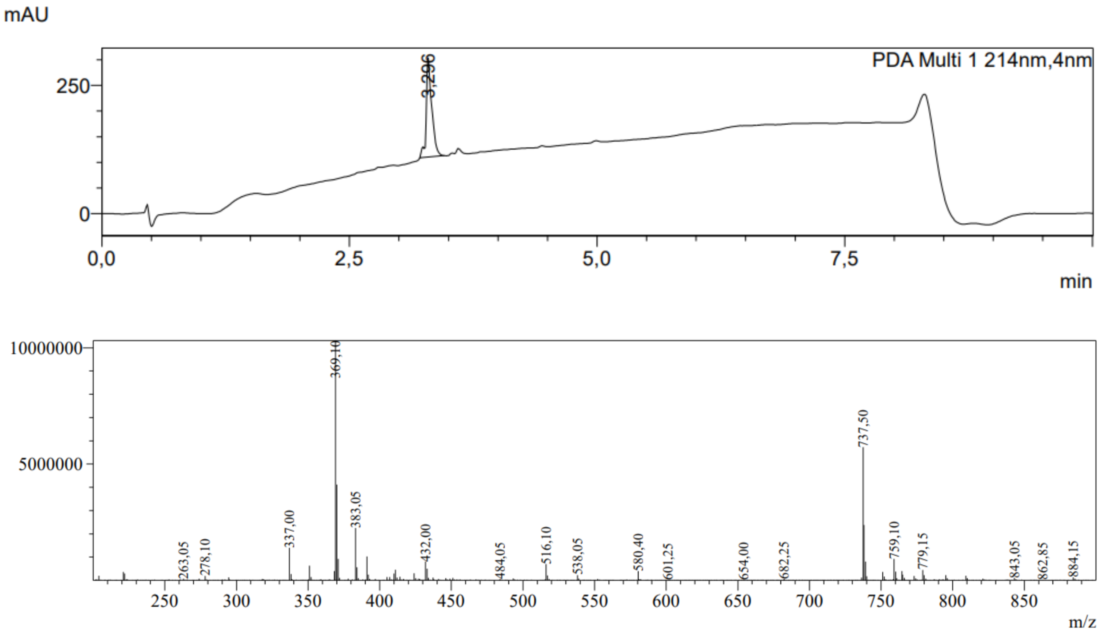

Figure S13: LC/MS analysis: HPLC chromatogram (top) and ESI-MS spectrum (bottom).

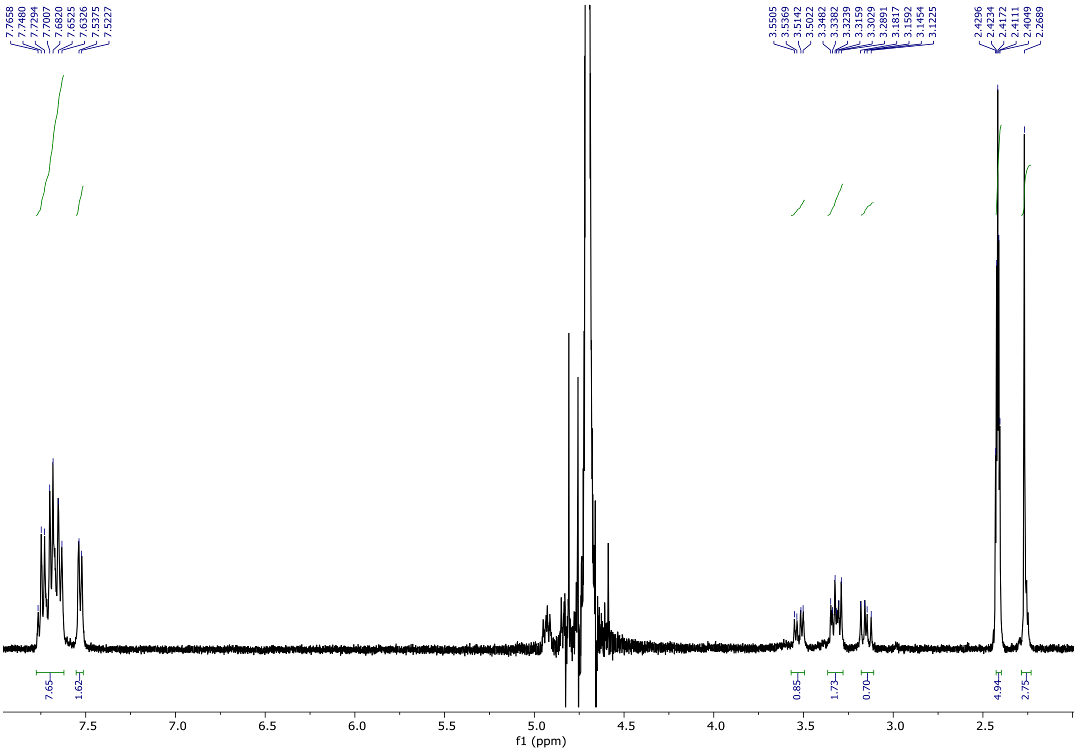

Figure S14: ^{1}H NMR (400 MHz, CD_3CN/D_2O 1/1) spectrum of **F_2-Hyd**.

A_3-Hyd. The modified amino acid was synthesized using Fmoc-*L*-Ala-OH. The crude product was obtained after precipitation in Et_2O and freeze drying. The compound **A_3-Hyd** was isolated

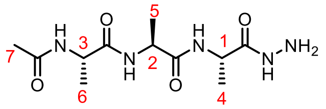

by preparative HPLC purification (0% B for 15 min, then → 20% B in 15 min). Yield: 75 %. ^{1}H NMR (400 MHz, D_2O): δ = 4.39 – 4.23 (m, 3H, H_1 – H_3), 2.02 (s, 3H, H_7), 1.44 – 1.36 (m, 9H, H_4 – H_6); LC/MS: t_R 0.537 min; ESI-MS calcd for m/z $[C_{11}H_{21}N_5O_4+H]^+$ 288.16 found 288.00; $[2(C_{11}H_{21}N_5O_4)+H]^+$ 575.32, found 575.10.

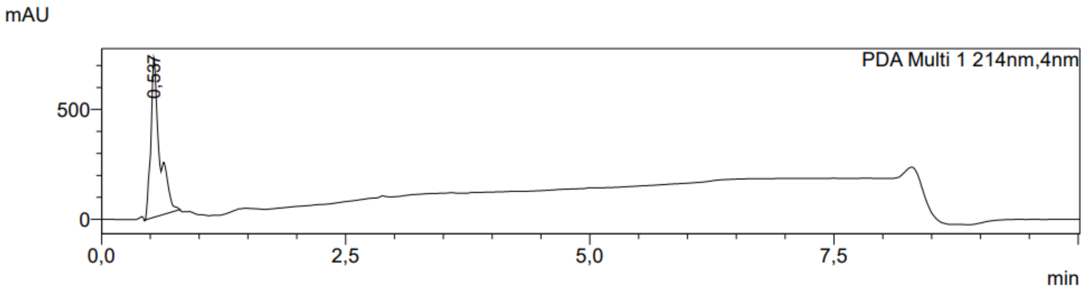

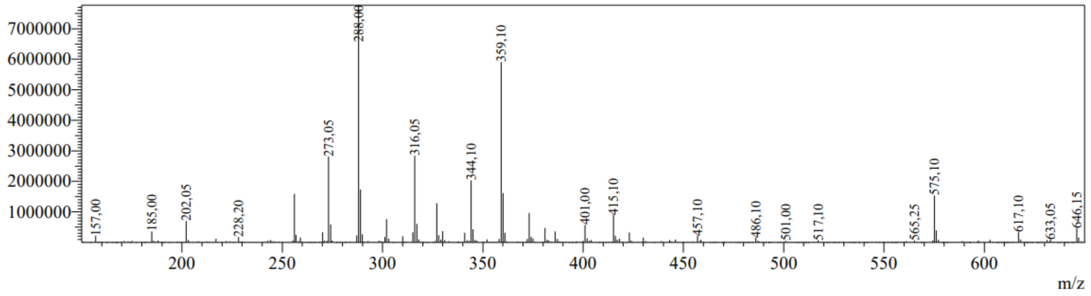

Figure S15: LC/MS analysis: HPLC chromatogram (top) and ESI-MS spectrum (bottom).

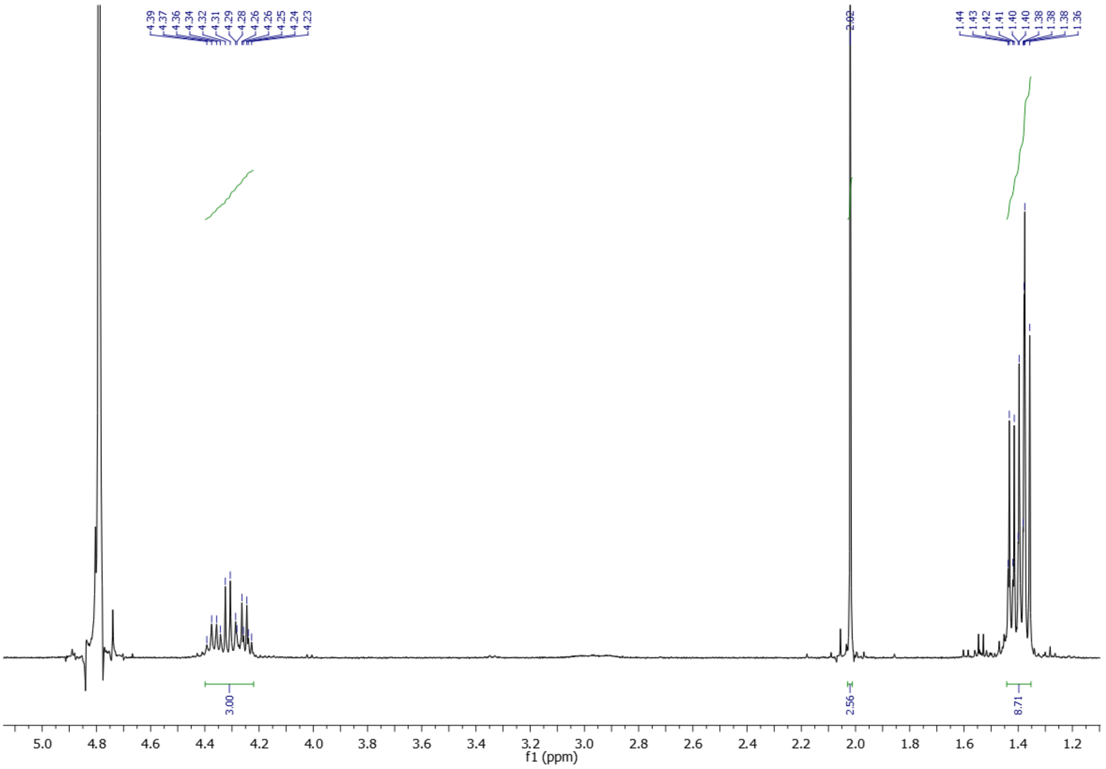

Figure S16: ^{1}H NMR (400 MHz, D_2O) spectrum of **A_3-Hyd.**

W_3-Hyd. W_3-Hyd was synthesized according the general SPPS procedure using Fmoc-*L*-Trp(Boc)-OH. The desired product was isolated by preparative HPLC purification (10% B for 5 min, then → 70% B in 50 min). Yield: 26 %. ^{1}H NMR (400 MHz, CD_3CN/D_2O 1/1): δ = 7.42 – 7.30 (m, 5H, H_{Ar}), 7.13 – 6.99 (m, 6H, H_{Ar}), 6.88 – 6.84 (m, 1H, H_{Ar}), 6.81 – 6.79 (m, 2H, H_{Ar}), 6.54 (s, 1H, H_{Ar}), 4.58 (q, *J* = 5.6, 1H, $H_α$), 4.29 (q, *J* = 6.4, 1H, $H_α$), 3.11 – 3.00 (m, 2H, $H_β$), 2.95 – 2.79 (m, 4H, $H_β$), 1.44 (s, 3H, CH_3); LC/MS: t_R 4.47 min; ESI-MS calcd for m/z $[C_{35}H_{36}N_8O_4+H]^+$ 632.29, found 633.30; $[2(C_{35}H_{36}N_8O_4)+H]^+$ 1265.58, found 1265.40.

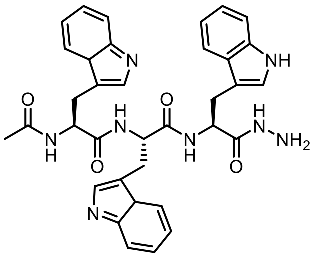

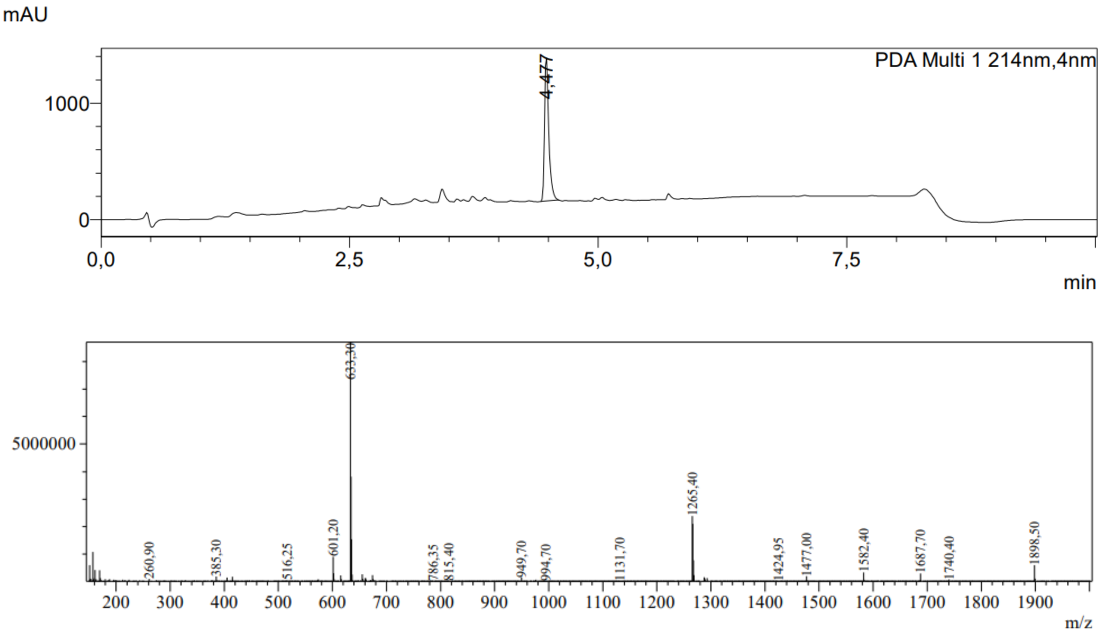

Figure S17: LC/MS analysis: HPLC chromatogram (top) and ESI-MS spectrum (bottom).

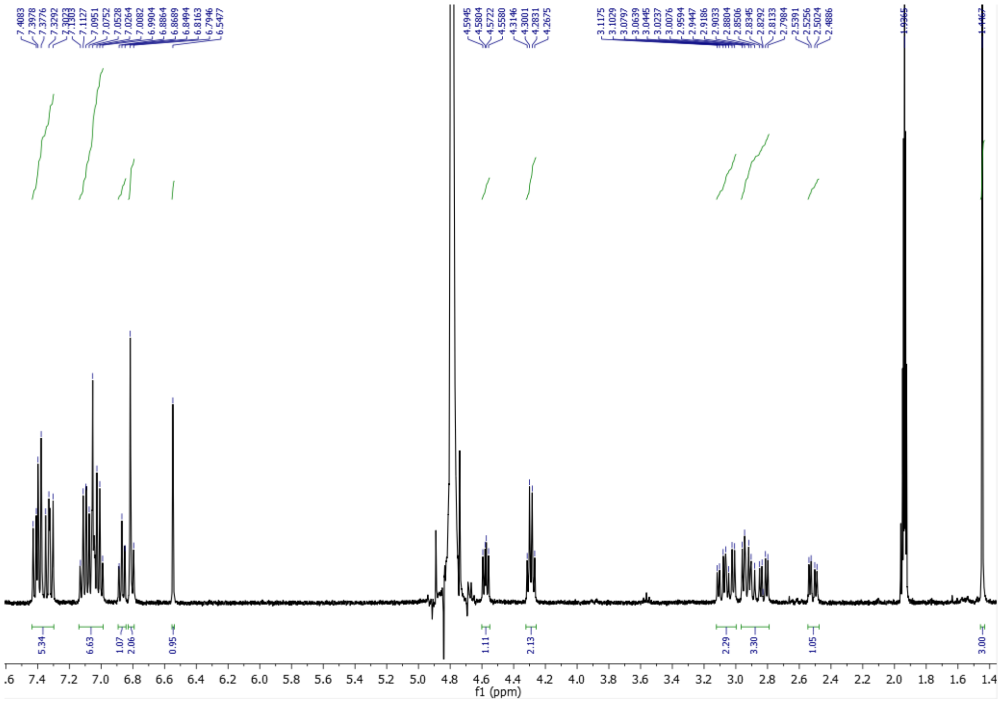

Figure S18: ^{1}H NMR (400 MHz, CD_3CN/D_2O 1/1) spectrum of **W_3-Hyd**.

F_3-Hyd. The modified amino acid was synthesized using Fmoc-*L*-Phe-OH. The crude product was obtained after precipitation in Et_2O and freeze drying. The compound **F_3-Hyd** was isolated by preparative HPLC purification (20% B for 5 min, then → 70% B in 50 min). Yield: 32 %. ^{1}H NMR (400 MHz, MeOD): 7.39 – 7.15 (m, 15H, H_{Ar}), 4.74 – 4.70 (dd, J = 5.8, 8.6, 1H, $H_α$), 4.55 – 4.52 (dd, J = 5.1, 9.4, 1H, $H_α$), 4.13 – 4.09 (dd, J = 6.4, 7.6, 1H, $H_α$), 3.27 – 2.73 (m, 6H, CH_2), 1.86 (s, 3H, CH_3); LC/MS: t_R 4.30 min; ESI-MS calcd for m/z $[C_{29}H_{33}N_5O_4+H]^+$ 516.25, found 516.10; $[C_{29}H_{33}N_5O_4-H]^-$ 514.24, found 514.05.

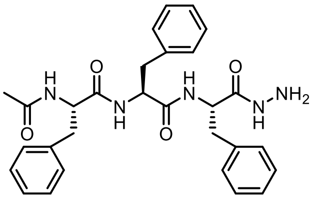

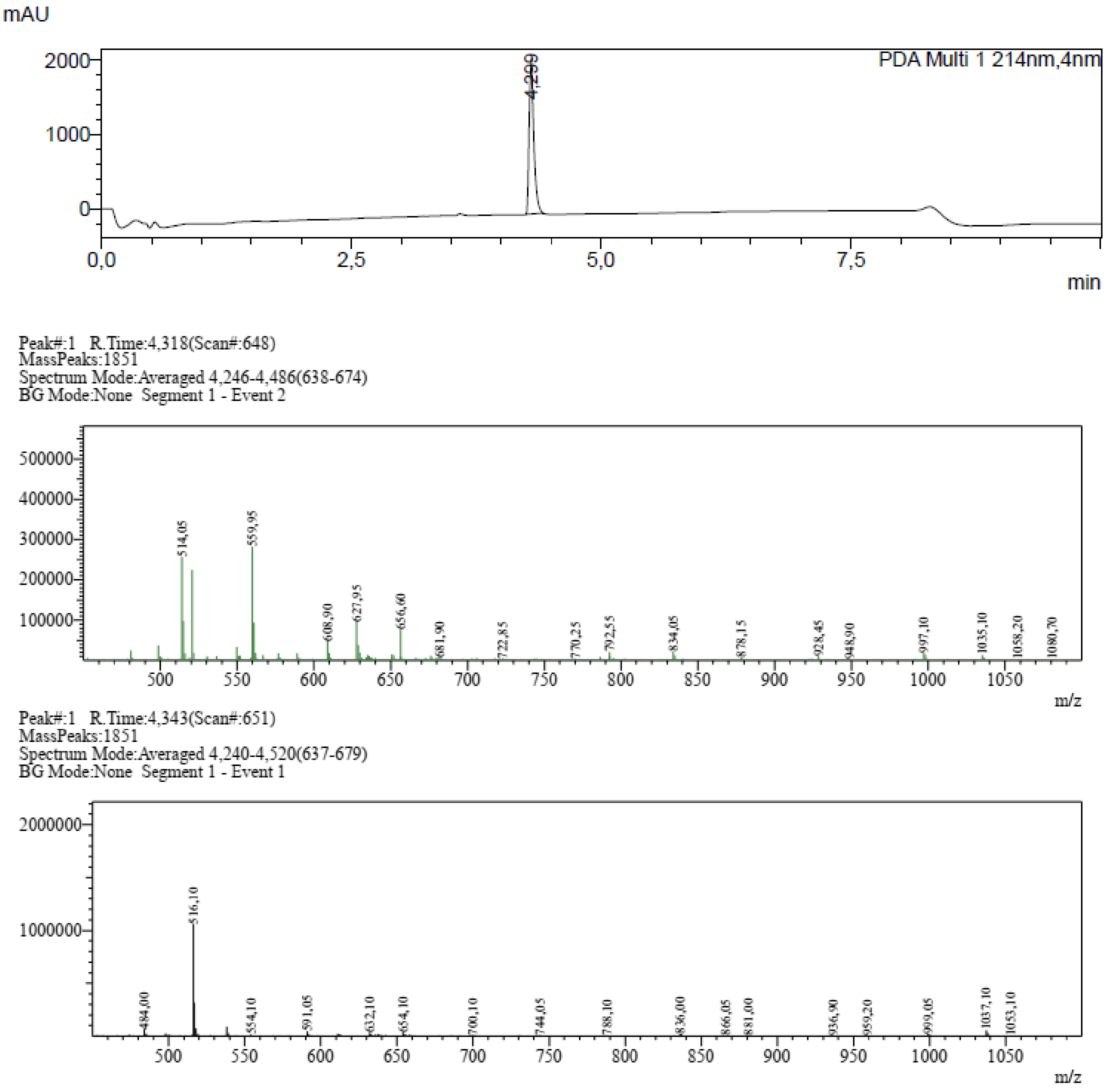

Figure S19: LC/MS analysis: HPLC chromatogram (top) and ESI-MS spectrum (middle: negative mode; bottom: positive mode).

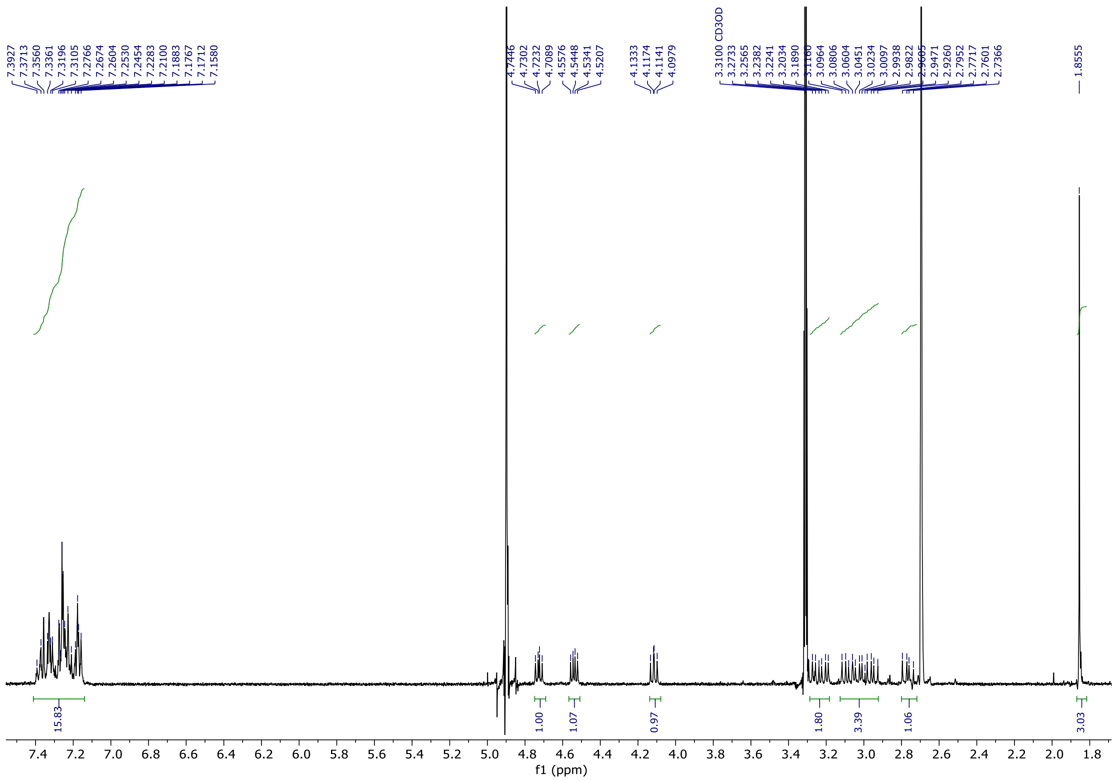

Figure S20: ^1H NMR (400 MHz, CD_3CN/D_2O 1/1) spectrum of **F_3-Hyd**.

VEVE-Hyd. The modified amino acid was synthesized using Fmoc-*L*-Val-OH and Fmoc-*L*-Glu(O*t*Bu)-OH. The crude product was obtained after precipitation in Et_2O and freeze drying.

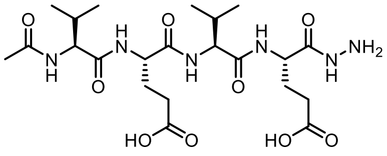

The compound **VEVE-Hyd** was isolated by preparative HPLC purification (0% B for 5 min, then → 40% B in 40 min). Yield: 65 %. ^1H NMR (400 MHz, D_2O): δ = 4.34 (q, *J* = 4.0, 1H, H_α), 4.19 (t, *J* = 7.9, 1H, H_α), 4.11, (d, *J* = 7.2, 2H, H_α), 2.30 – 2.18 (m, 4H, CH_2/CH), 2.06 (s, 3H, C*H*$_3$CO), 2.01 – 1.90 (m, 6H, H_β), 0.95 (d, *J* = 6.8, 12H, C*H*$_3$CH); LC/MS: t_R 1.85 min; ESI-MS calcd for m/z $[C_{22}H_{38}N_6O_9+H]^+$ 531.25, found 531.15; $[2(C_{22}H_{38}N_6O_9)+H]^+$ 1061.20, found 1061.40.

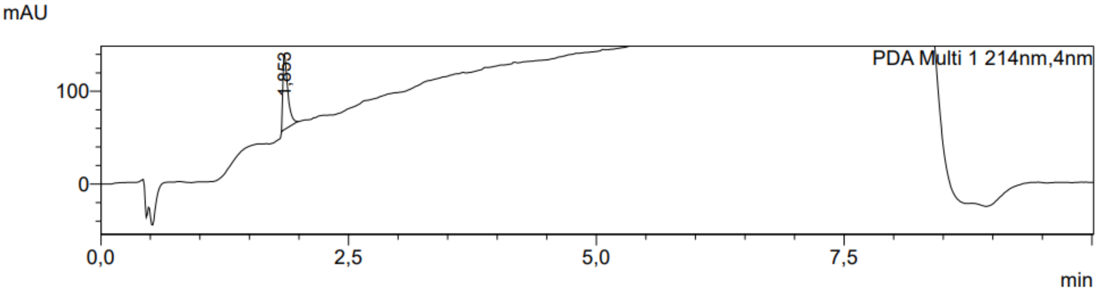

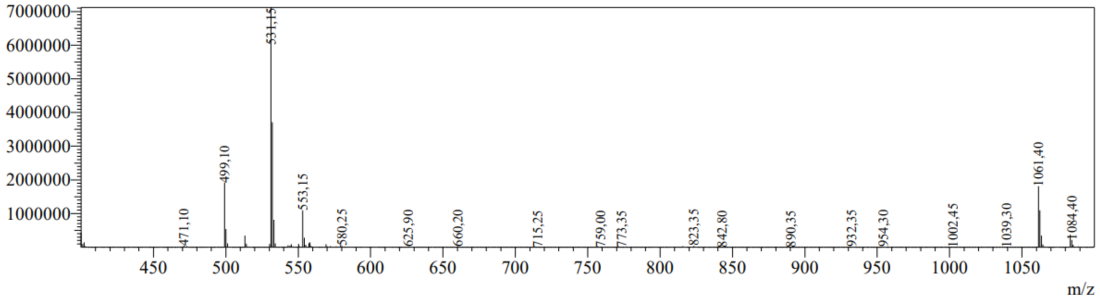

Figure S21: LC/MS analysis: HPLC chromatogram (top) and HR-ESI-MS spectrum (bottom).

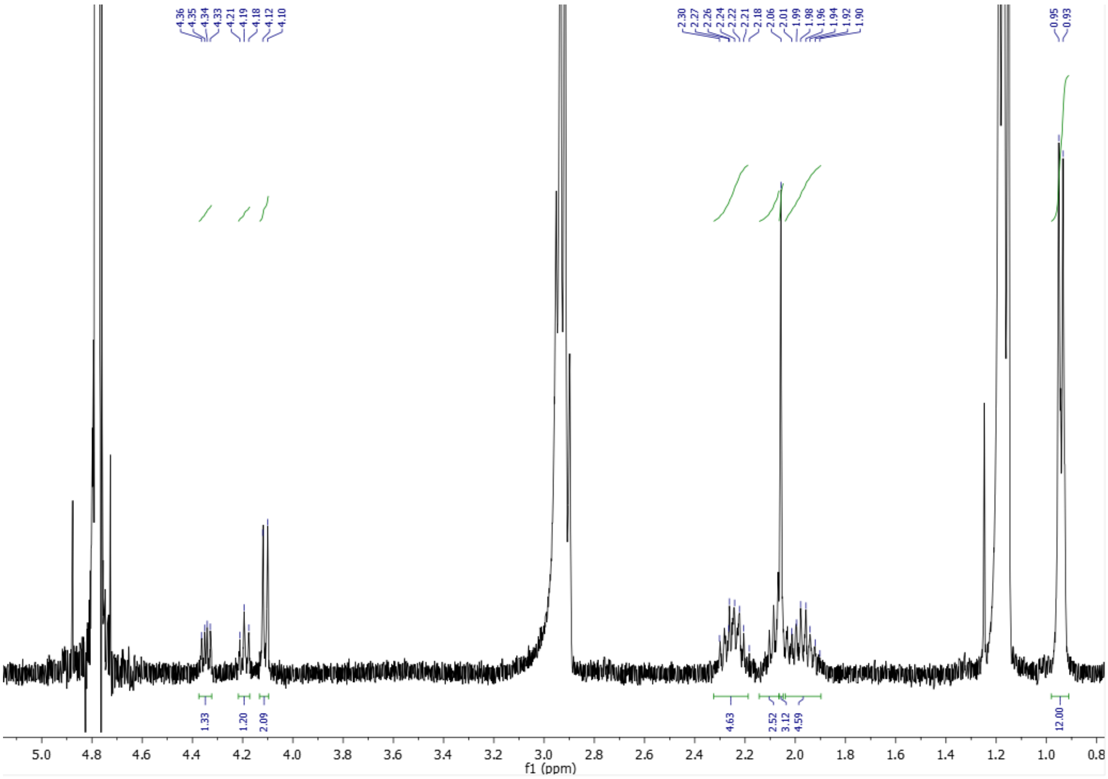

Figure S22: ^{1}H NMR (400 MHz, D_2O) spectrum of **VEVE-Hyd**.

EVEV-Hyd. The modified amino acid was synthesized using Fmoc-*L*-Val-OH and Fmoc-*L*-Glu(O*t*Bu)-OH. The crude product was obtained after precipitation in Et_2O and freeze drying.

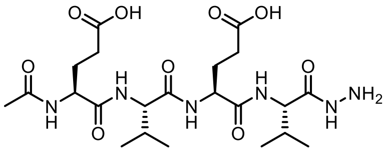

The compound **EVEV-Hyd** was isolated by preparative HPLC purification (0% B for 5 min, then → 40% B in 40 min). Yield: 63 %. ^{1}H NMR (400 MHz, D_2O): δ = 4.46 (q, *J* = 4.0, 1H, H_α), 4.36 (q, *J* = 7.9, 1H, H_α), 4.15, (m, 2H, H_α), 2.49 – 2.46 (m, 4H, CH_α), 2.13 – 2.07 (m, 4H, H_β), 2.03 (s, 3H, CH_3CO), 2.03 – 1.96 (m, 2H, CH), 1.01 - 0.91 (m, 12H, CH_3CH); LC/MS: t_R 1.88 min; ESI-MS calcd for m/z $[C_{22}H_{38}N_6O_9+H]^+$ 531.25, found 531.15; $[2(C_{22}H_{38}N_6O_9)+H]^+$ 1061.20, found 1061.45.

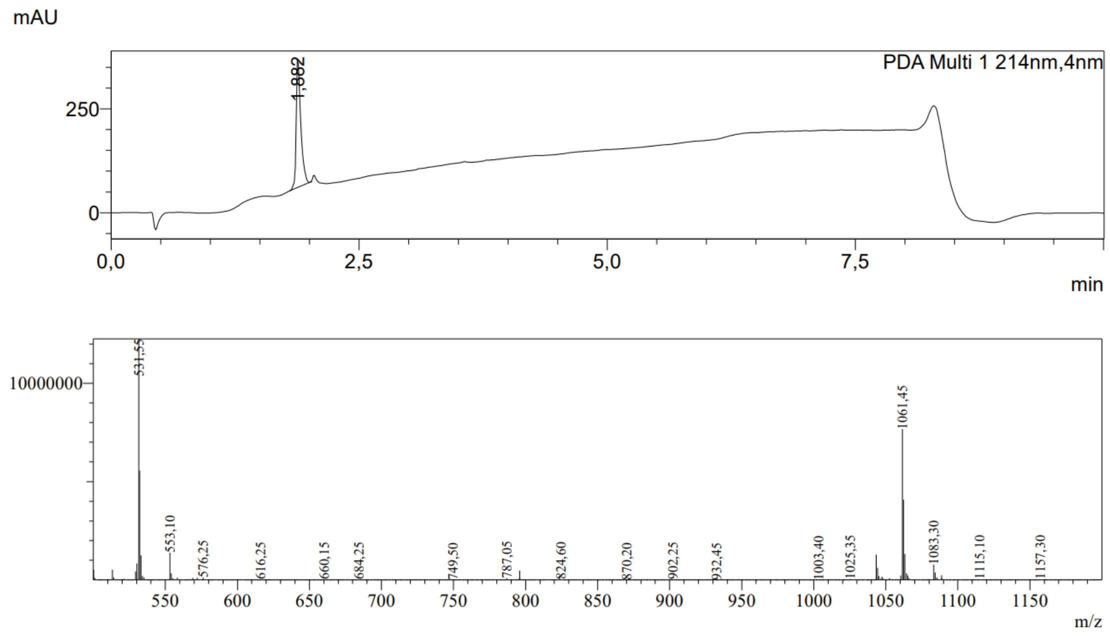

Figure S23: LC/MS analysis: HPLC chromatogram (top) and ESI-MS spectrum (bottom).

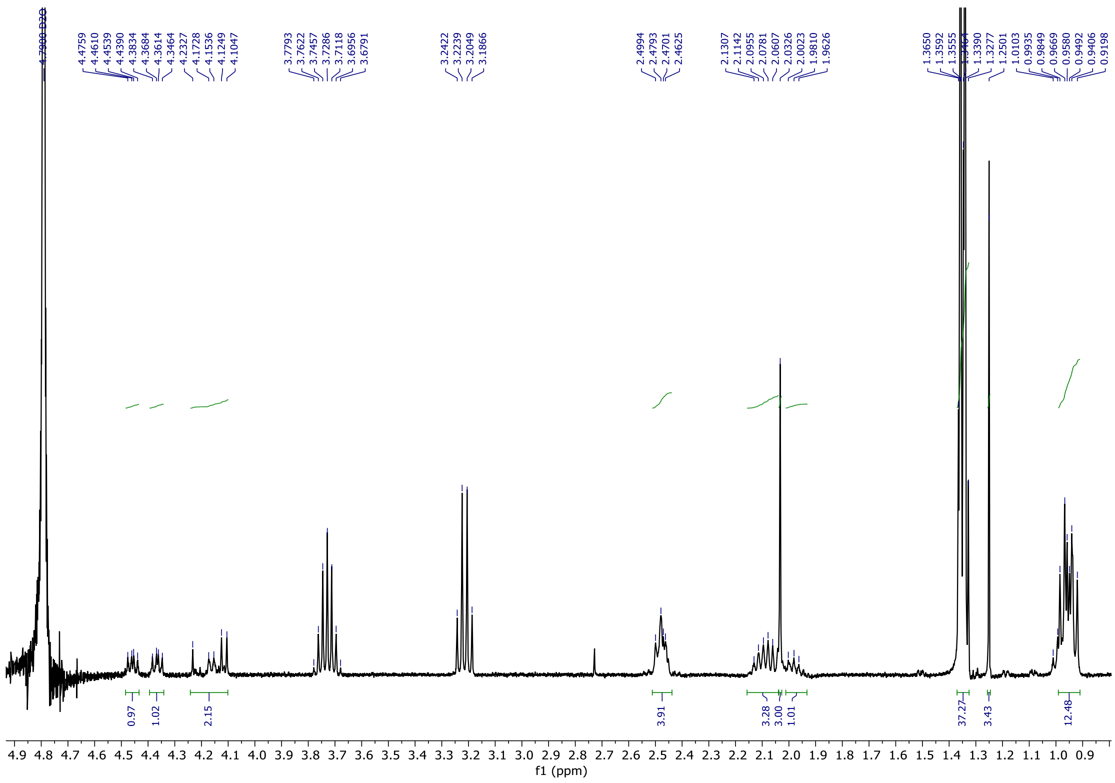

Figure S24: ^{1}H NMR (400 MHz, D_2O) spectrum of **EVEV-Hyd**.

LLKK-Hyd. The modified amino acid was synthesized using Fmoc-*L*-Leu-OH and Fmoc-*L*-Lys(Boc)-OH. The crude product was obtained after precipitation in Et_2O and freeze drying. The

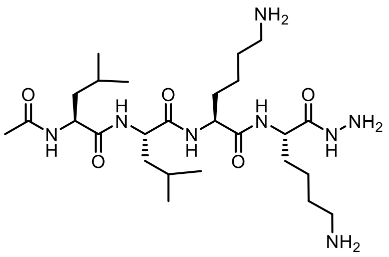

compound **LLKK-Hyd** was isolated by preparative HPLC purification (0% B for 5 min, then → 40% B in 40 min). Yield: 57 %. ^{1}H NMR (400 MHz, D_2O): δ = 4.38 – 4.32 (m, 3H, H_α), 4.25 – 4.22 (m, 1H, H_α), 3.00 (t, J = 7.2, 4H, CH_2NH_2), 2.04 (s, 3H, CH_3CO), 1.82 – 1.38 (m, 18H, CH, CH_2), 0.94 (t, J = 6.2, 6H, CH_3CH), 0.88 (d, J = 4.3, 6H, CH_3CH); LC/MS: t_R 0.63 min; ESI-MS calcd for m/z $[C_{24}H_{48}N_8O_5+H]^+$ 529.37, found 529.40; $[2(C_{24}H_{48}N_8O_5)+H]^+$ 1057.74, found 1057.60; $[C_{24}H_{48}N_8O_5+2H]^{2+}$ 265.19, found 265.25; $[C_{24}H_{48}N_8O_5+ACN+2H]^{2+}$ 285.70, found 285.80.

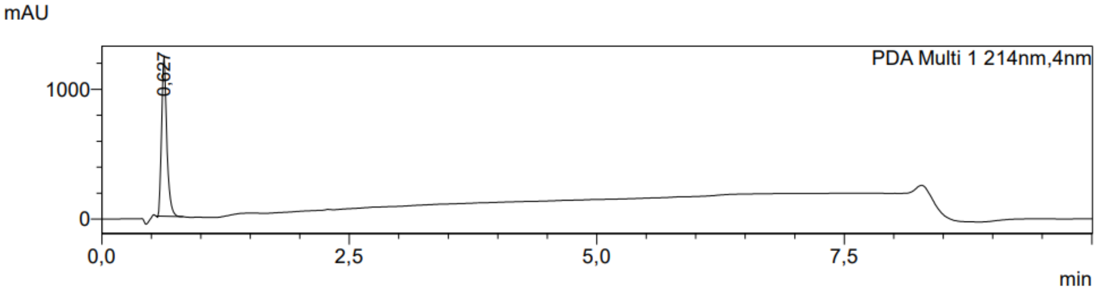

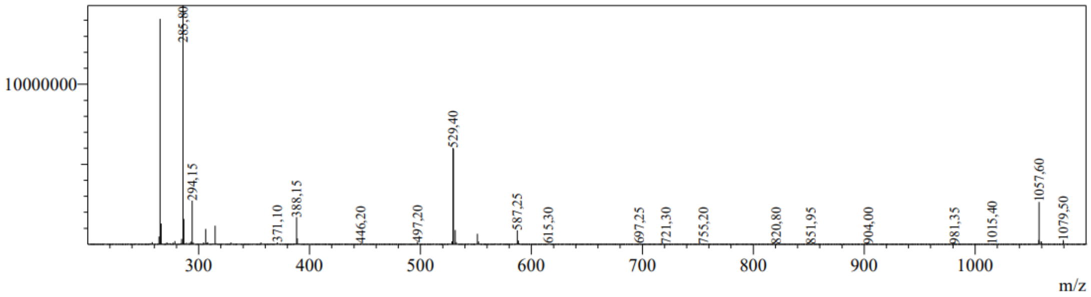

Figure S25: LC/MS analysis: HPLC chromatogram (top) and ESI-MS spectrum (bottom).

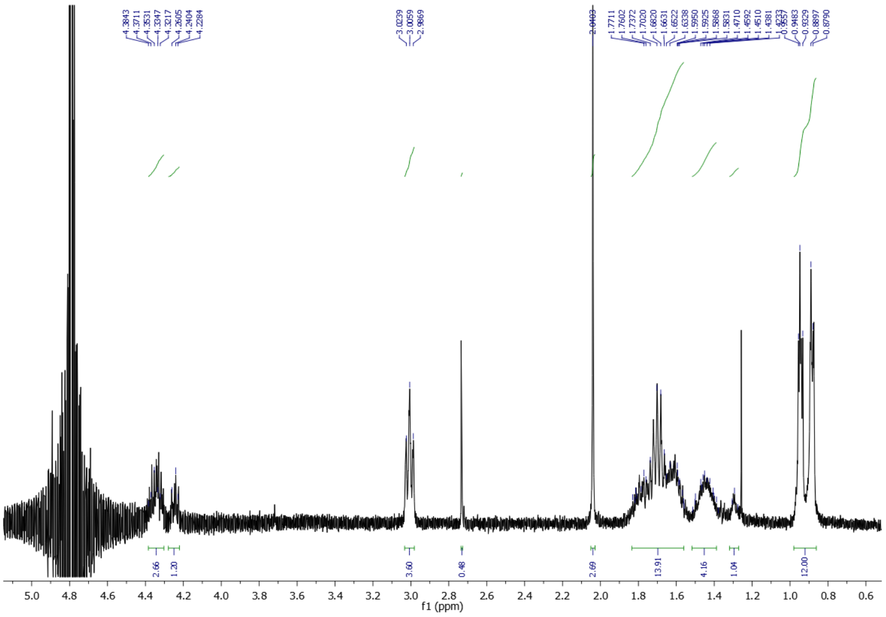

Figure S26: ^{1}H NMR (400 MHz, D_2O) spectrum of **LLKK-Hyd**.

KKLL-Hyd. The modified amino acid was synthesized using Fmoc-*L*-Val-OH and Fmoc-*L*-Lys(Boc)-OH. The crude product was obtained after precipitation in Et_2O and freeze drying. The compound **KKLL-Hyd** was isolated by preparative HPLC purification (0% B for 5 min, then → 40% B in 40 min). Yield: 45 %. ^1H NMR (400 MHz, D_2O): δ = 4.40 – 4.31 (m, 3H, H_α), 4.25 – 4.21 (m, 1H, H_α), 3.00 (t, 4H, CH_2NH_2), 2.03 (s, 3H, CH_3CO), 1.80 – 1.60 (m, 14H, CH_2), 1.44 – 1.43 (m, 4H, CH_2), 1.25 (s, *t*BuOH), 0.94 (t, *J* = 6.2, 6H, CH_3CH), 0.88 (t, *J* = 4.3, 6H, CH_3CH); LC/MS: t_R 1.64 min; ESI-MS calcd for m/z $[C_{26}H_{53}N_8O_5+H]^+$ 557.41, found 557.30.

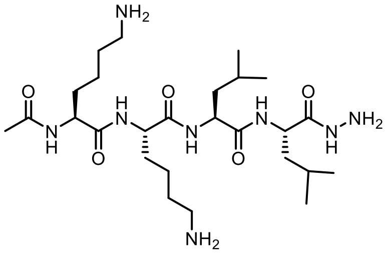

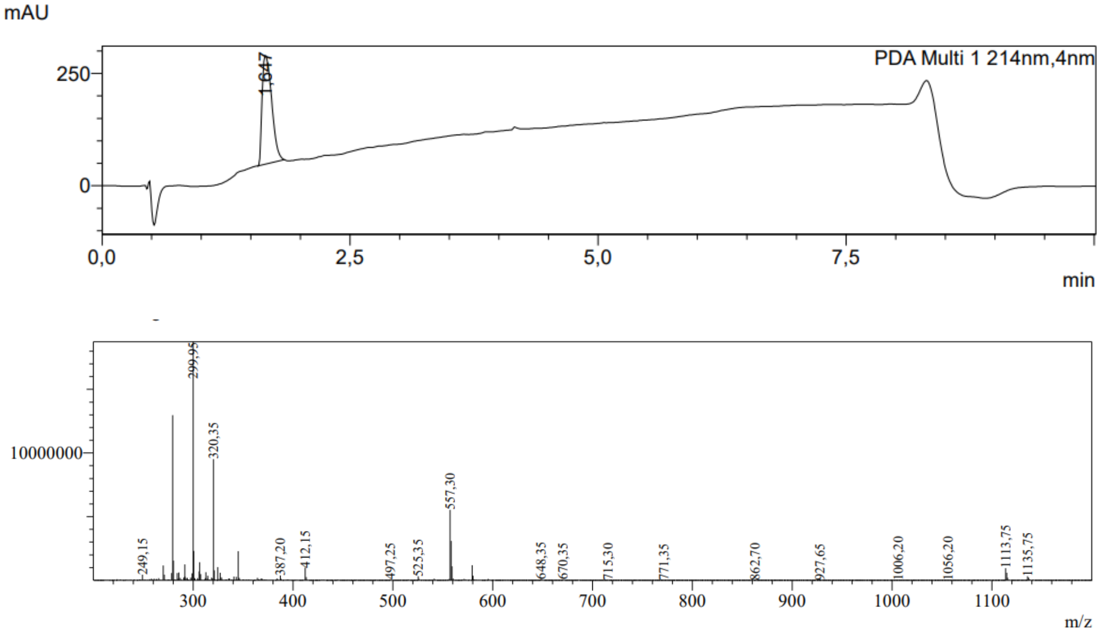

Figure S27: LC/MS analysis: HPLC chromatogram (top) and ESI-MS spectrum (bottom).

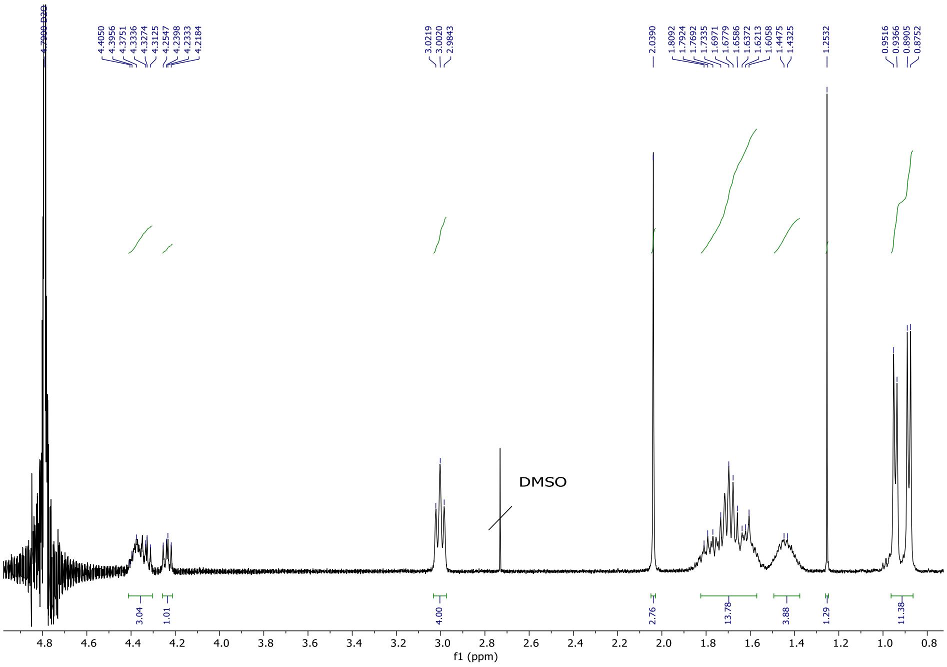

Figure S28: ^1H NMR (400 MHz, D_2O) spectrum of **KKLL-Hyd.**

GLKAK-Hyd. The modified amino acid was synthesized using Fmoc-*L*-Leu-OH, Fmoc-*L*-Ala-OH, Fmoc-Gly-OH and Fmoc-*L*-Lys(Boc)-OH. The crude product was obtained after precipitation in Et_2O and freeze drying. The compound **GLKAK-Hyd** was isolated by preparative HPLC purification (0% B for 5 min, then → 40% B in 40 min). Yield: 52 %. ^{1}H NMR (400 MHz, D_2O): δ = 4.27 – 4.19 (m, 4H, $H_α$), 3.85 (d, *J* = 3.4, 2H, $H_α$Gly), 2.94 (t, *J* = 7.0, 4H, CH_2NH_2), 1.98 (s, 3H, CH_3CO), 1.77 – 1.38 (m, 15H, CH_2, CH), 1.31 (d, *J* = 7.2, 3H, CH_3Ala), 0.85 (d, *J* = 6.0, 3H, CH_3CH), 0.82 (d, *J* = 6.0, 3H, CH_3CH); LC/MS: t_R 0.56 min; ESI-MS calcd for m/z $[C_{25}H_{49}N_9O_6+H]^+$ 572.38, found 572.30; $[2(C_{25}H_{49}N_9O_6)+H]^+$ 1143.76, found 1143.40; $[C_{25}H_{49}N_9O_6+2H]^{2+}$ 286.69, found 286.80; $[C_{25}H_{49}N_9O_6+ACN+2H]^{2+}$ 307.21, found 307.30.

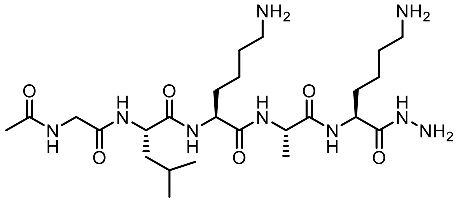

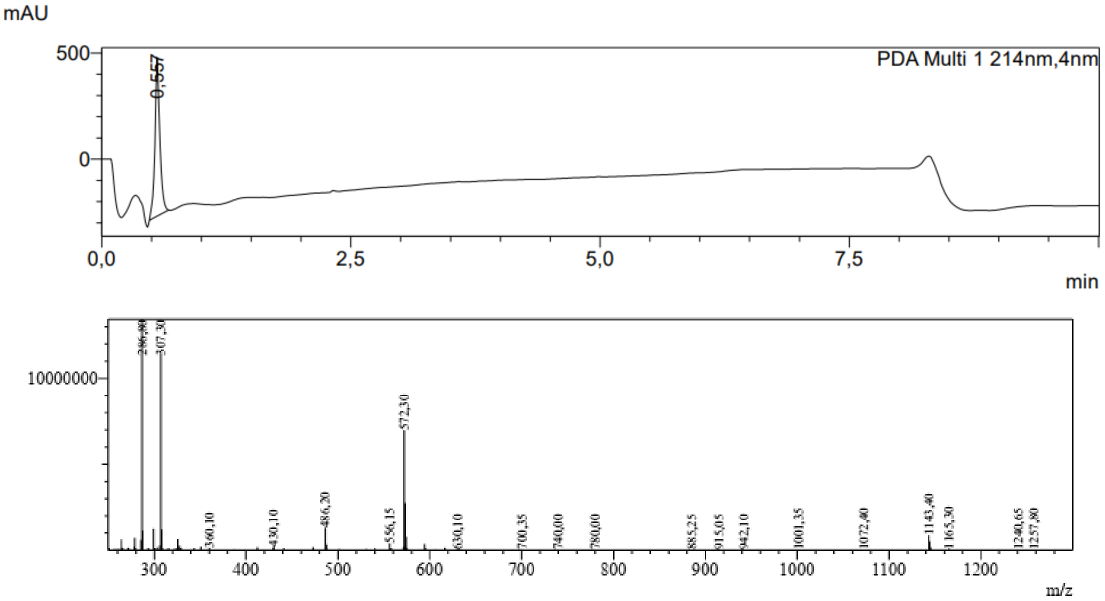

Figure S29: LC/MS analysis: HPLC chromatogram (top) and ESI-MS spectrum (bottom).

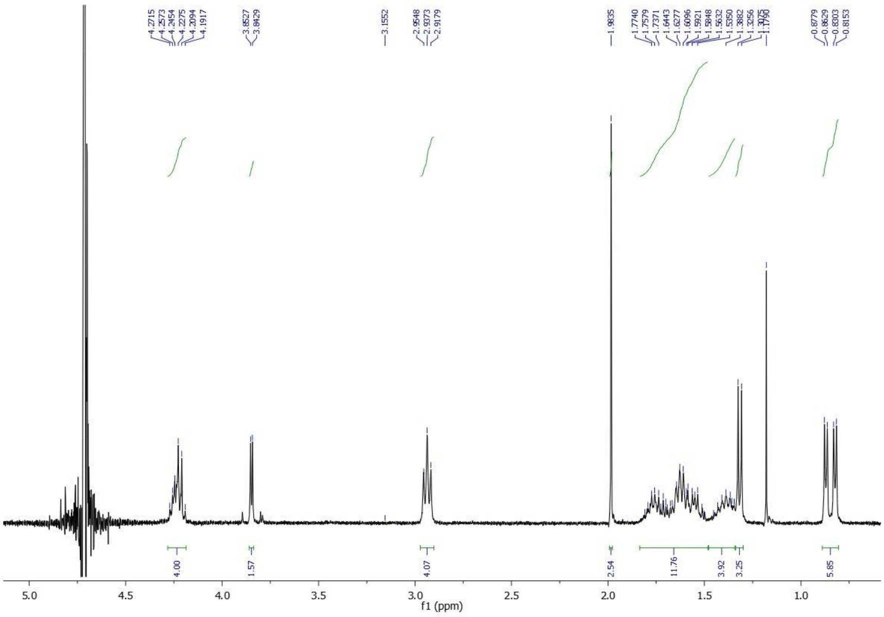

Figure S30: ^{1}H NMR (400 MHz, D_2O) spectrum of **GLKAK-Hyd.**

GLKLK-Hyd. The modified amino acid was synthesized using Fmoc-*L*-Leu-OH, Fmoc-Gly-OH and Fmoc-*L*-Lys(Boc)-OH. The crude product was obtained after precipitation in Et_2O and freeze drying. The compound **GLKLK-Hyd** was isolated by preparative HPLC purification (0% B for 5 min, then → 40% B in 40 min). Yield: 41 %. ^{1}H NMR (400 MHz, D_2O): δ = 4.38 – 4.31 (m, 4H, $H_α$), 3.93 (d, *J* = 4.7, 2H, $H_α$Gly), 3.02, (t, *J* = 6.8, 4H, C*H*$_2$NH$_2$), 2.07 (s, 3H, C*H*$_3$), 1.86 – 1.41 (m, 18H, CH_2, CH), 0.96 – 0.90 (m, 12H, C*H*$_3$CH); LC/MS: t_R 1.70 min; ESI-MS calcd for m/z $[C_{28}H_{55}N_9O_6+H]^+$ 614.43, found 614.25; $[C_{28}H_{55}N_9O_6+2H]^{2+}$ 307.72, found 307.85; $[C_{28}H_{55}N_9O_6+ACN+2H]^{2+}$ 328.23, found 328.35.

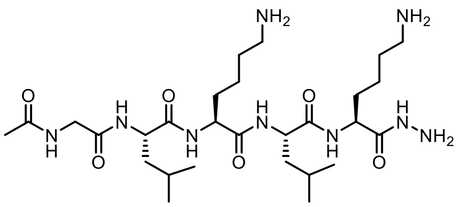

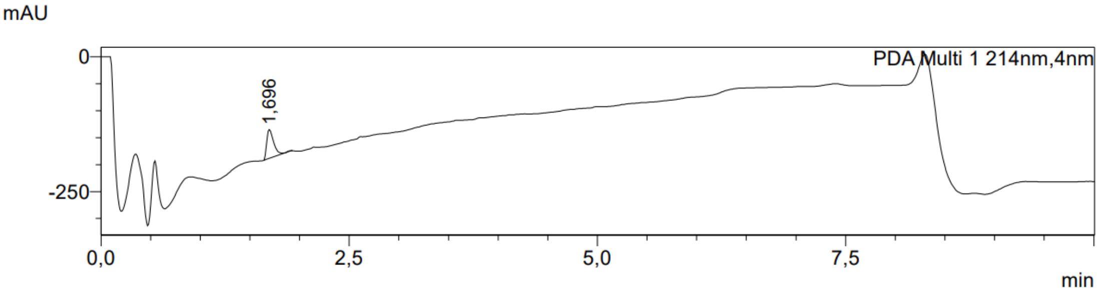

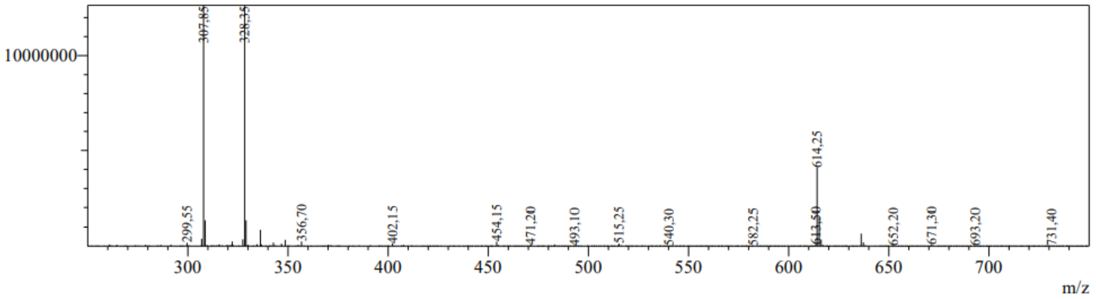

Figure S31: LC/MS analysis: HPLC chromatogram (top) and ESI-MS spectrum (bottom).

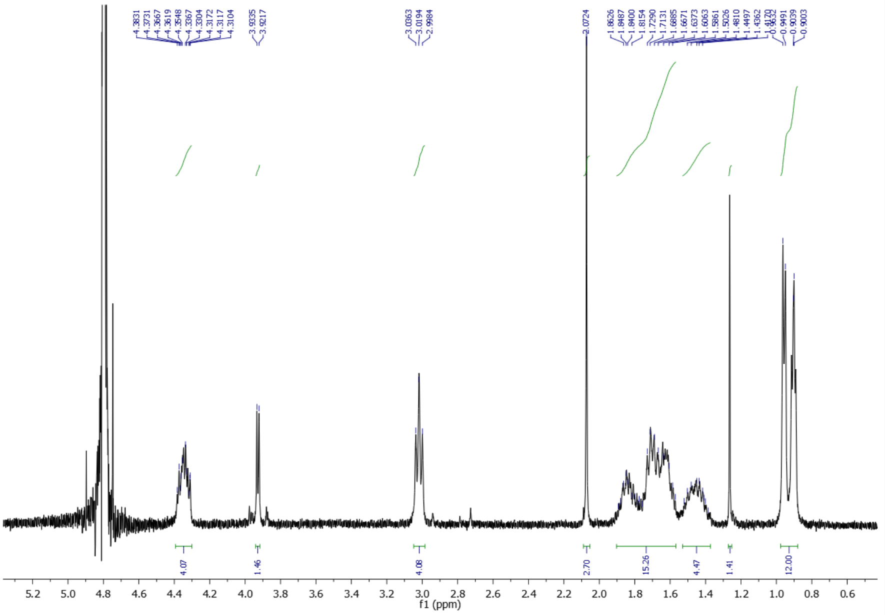

Figure S32: ^{1}H NMR (400 MHz, D_2O) spectrum of **GLKLK-Hyd.**

GLKFK-Hyd. The modified amino acid was synthesized using Fmoc-*L*-Leu-OH, Fmoc-*L*-Phe-OH, Fmoc-Gly-OH and Fmoc-*L*-Lys(Boc)-OH. The crude product was obtained after precipitation in Et_2O and freeze drying. The compound **GLKFK-Hyd** was isolated by preparative HPLC purification (0% B for 5 min, then → 40% B in 40 min). Yield: 25 %. ^{1}H NMR (400 MHz, D_2O): δ = 7.40 – 7.35 (m, 3H, H_{ar}), 7.27 – 7.24 (m, 2H, H_{ar}), 4.60 (t, *J* = 8.0, 1H, H_α), 4.31 – 4.25 (m, 3H, H_α), 3.92 (d, *J* = 6.8, 2H, H_αGly), 2.04 (s, 3H, CH_3), 1.71 – 1.52 (m, 17H, CH_2, CH), 0.95 (d, *J* = 6.0, 3H, CH_3CH), 0.89 (d, *J* = 6.1, 3H, CH_3CH); LC-MS: t_R 1.64 min; ESI-MS calcd for mz $[C_{31}H_{53}N_9O_6+H]^+$ 648.41, found 648.60; $[2(C_{31}H_{53}N_9O_6)+H]^+$ 1295.83, found 1295.35; $[C_{31}H_{53}N_9O_6+2H]^{2+}$ 324.71, found 324.85.

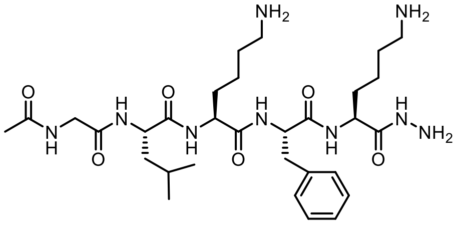

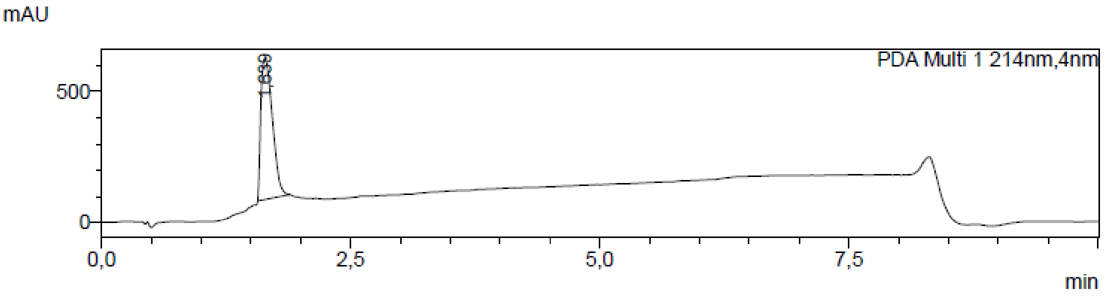

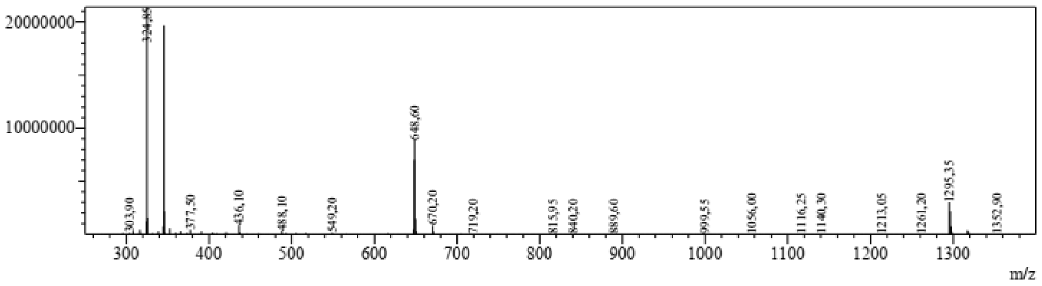

Figure S33: LC/MS analysis: HPLC chromatogram (top) and ESI-MS spectrum (bottom).

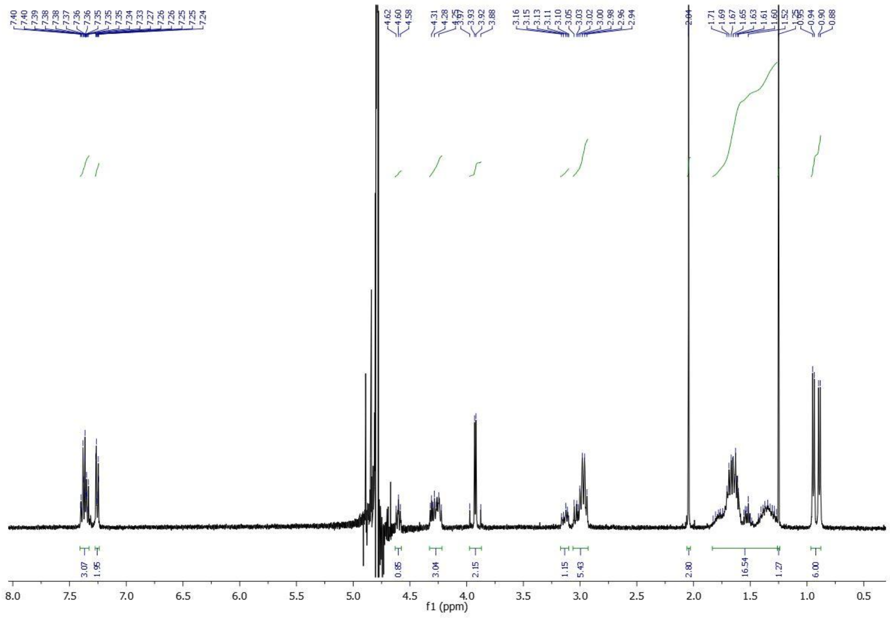

Figure S34: ^{1}H NMR (400 MHz, D_2O) spectrum of **GLKFK-Hyd.**

GLKSK-Hyd. The modified amino acid was synthesized using Fmoc-*L*-Leu-OH, Fmoc-*L*-Ser(O*t*Bu)-OH, Fmoc-Gly-OH and Fmoc-*L*-Lys(Boc)-OH. The crude product was obtained after

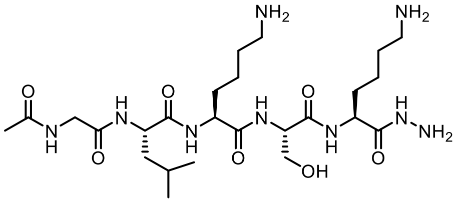

precipitation in Et_2O and freeze drying. The compound **GLKSK-Hyd** was isolated by preparative HPLC purification (0% B for 5 min, then → 40% B in 40 min). Yield: 33 %. ^1H NMR (400 MHz, D_2O): δ = 4.42 (t, *J* = 6.0, 1H, $H_α$), 4.37 – 4.32 (m, 3H, $H_α$), 3.92 (s, 2H, $H_α$Gly), 3.90 – 3.82 (m, 2H, CH_2OH), 3.01 (t, *J* = 5.8 , 4H, CH_2NH_2), 2.06 (s, 3H, CH_3), 1.89 – 1.43 (m, 15H, CH_2, CH), 0.94 (d, *J* = 5.7, 3H, CH_3CH), 0.90 (d, *J* = 5.9, 3H, CH_3CH); LC-MS: t_R 0.56 min; ESI-MS calcd for m/z $[C_{25}H_{49}N_9O_7+H]^+$ 588.37, found 588.45; $[C_{25}H_{49}N_9O_7+2H]^{2+}$ 294.68, found 294.80.

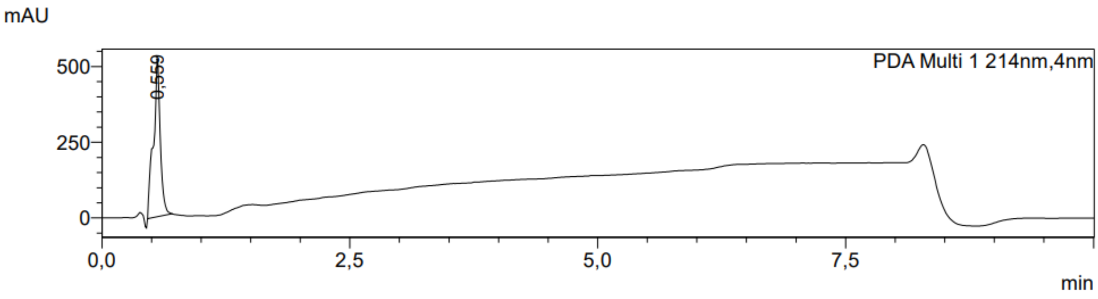

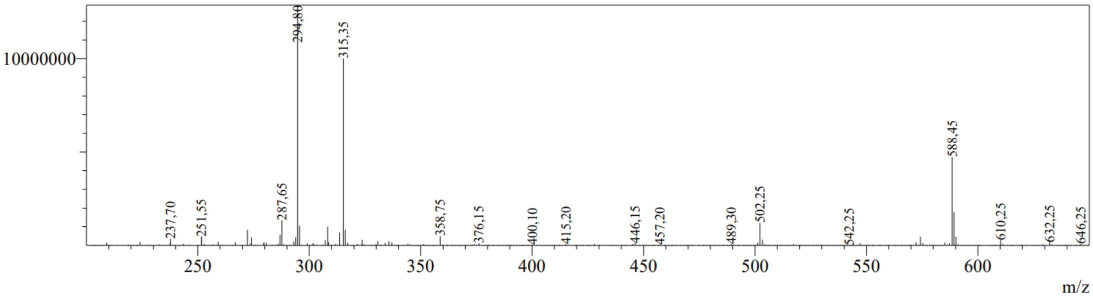

Figure S35: LC/MS analysis: HPLC chromatogram (top) and ESI-MS spectrum (bottom).

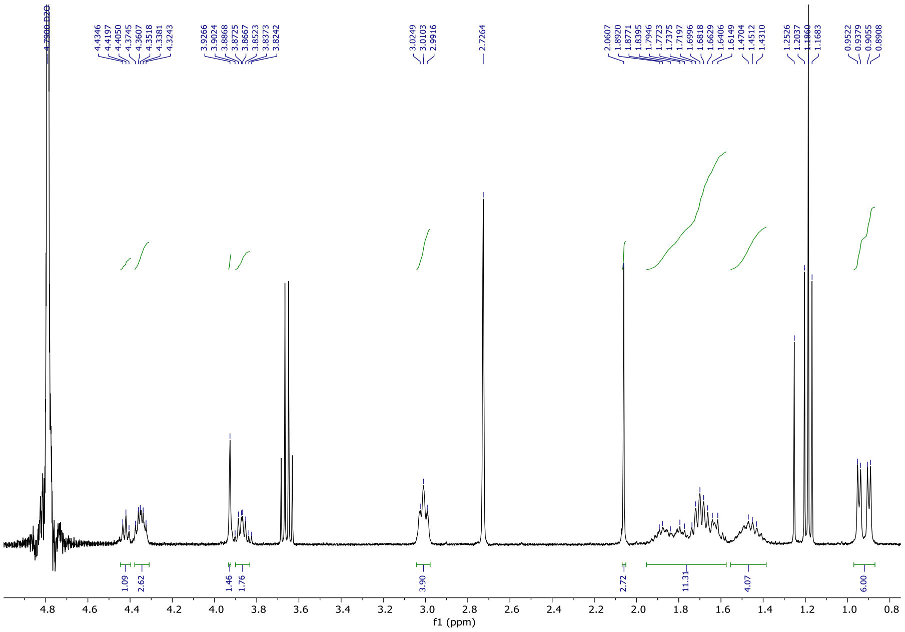

Figure S36: ^1H NMR (400 MHz, D_2O) spectrum of **GLKSK-Hyd.**

KLKLG-Hyd. The modified amino acid was synthesized using Fmoc-*L*-Leu-OH, Fmoc-Gly-OH and Fmoc-*L*-Lys(Boc)-OH. The crude product was obtained after precipitation in Et_2O and freeze drying. The compound **KLKLG-Hyd** was isolated by preparative HPLC purification (0% B for 5 min, then → 40% B in 40 min). Yield: 39%. ^1H NMR (400 MHz, D_2O): δ = 4.43 – 4.31 (m, 3H, $H_α$), 4.26 – 4.22 (m, 1H, $H_α$), 3.98 – 3.94 (m, 2H, $H_α$), 3.00 (t, J = 7.7, 4H, CH_2NH_2), 2.04 (s, 3H, CH_3), 1.83 – 1.42 (m, 18H, CH_2, CH), 0.94 (d, J = 5.8, 6H, CH_3CH), 0.89 (d, J = 6.0, 6H, CH_3CH); LC/MS: t_R 1.62 min, ESI-MS calcd for m/z $[C_{28}H_{55}N_9O_6+H]^+$ 614.43, found 614.50; $[2(C_{28}H_{55}N_9O_6)+H]^+$ 1227.86, found 1227.60; $[C_{28}H_{55}N_9O_6+2H]^{2+}$ 307.72, found 307.95; $[C_{28}H_{55}N_9O_6+ACN+2H]^{2+}$ 328.23, found 328.45.

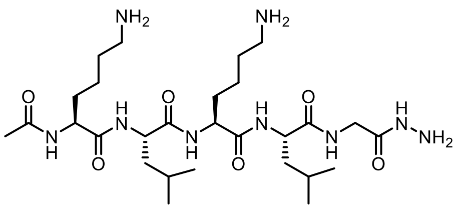

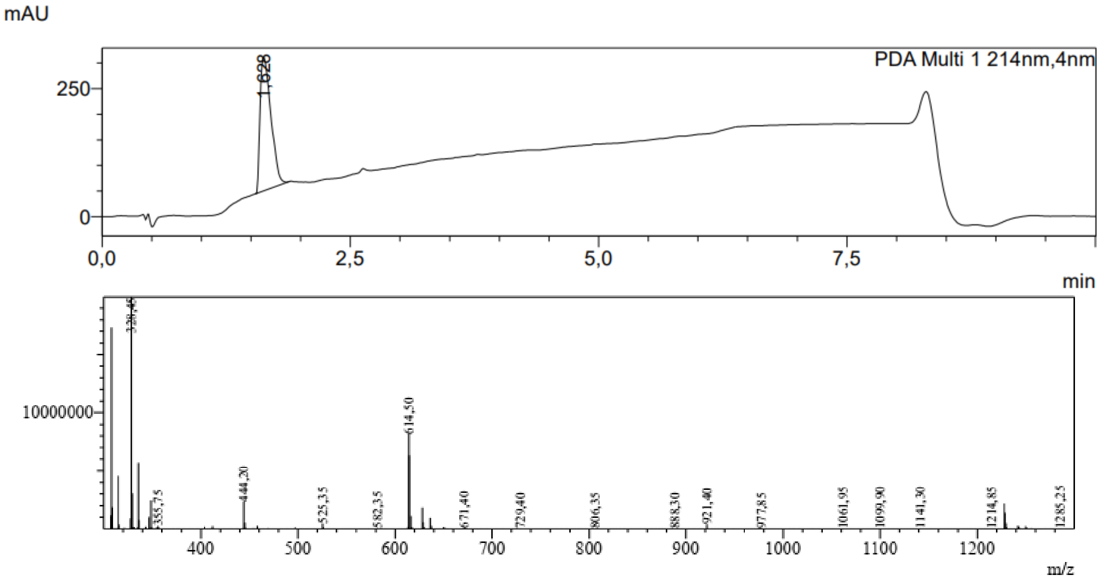

Figure S37: LC/MS analysis: HPLC chromatogram (top) and ESI-MS spectrum (bottom).

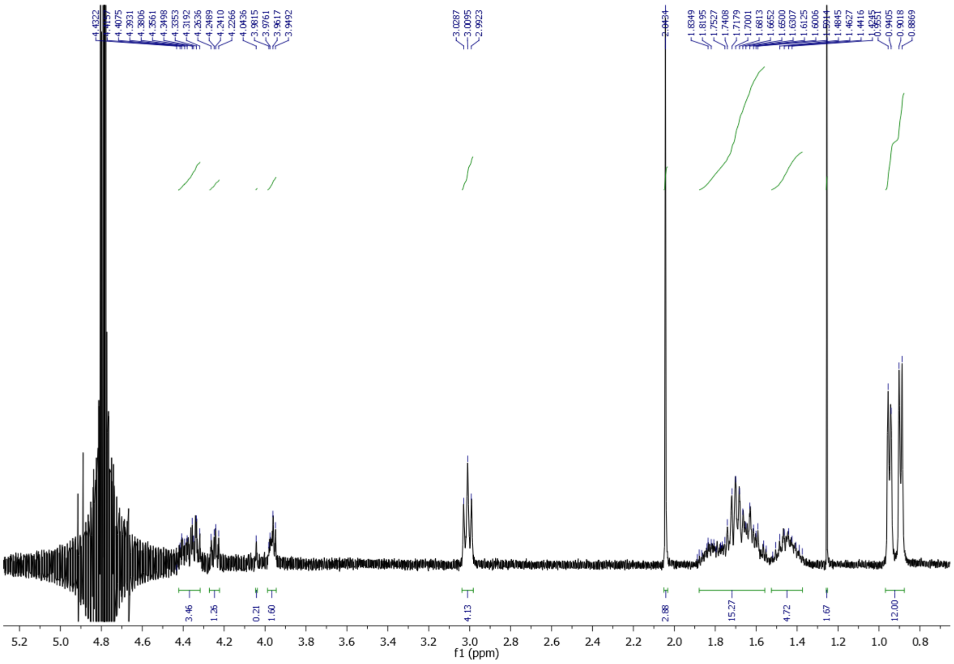

Figure S38: ^1H NMR (400 MHz, D_2O) spectrum of **KLKLG-Hyd.**

KAKLG-Hyd. The modified amino acid was synthesized using Fmoc-*L*-Leu-OH, Fmoc-*L*-Ala-OH, Fmoc-Gly-OH and Fmoc-*L*-Lys(Boc)-OH. The crude product was obtained after precipitation in Et_2O and freeze drying. The compound **KAKLG-Hyd** was isolated by preparative HPLC purification (0% B for 5 min, then → 40% B in 40 min). Yield: 45 %. ^{1}H NMR (400 MHz, D_2O): δ = 4.36 – 4.29 (m, 1H, $H_α$), 4.28 – 4.15 (m, 3H, $H_α$), 3.92 (m, 2H, $H_α$), 2.94 (t, J = 8.0, 4H, CH_2NH_2), 1.97 (s, 3H, CH_3), 1.77 – 1.34 (m, 15H, CH_2, CH), 1.32 (d, J = 5.6, 3H, CH_3Ala), 0.87 (d, J = 6.0, 3H, CH_3CH), 0.81 (d, J = 6.0, 3H, CH_3CH); LC/MS: t_R 0.65 min; ESI-MS calcd for m/z $[C_{25}H_{49}N_9O_6+H]^+$ 572.38, found 572.30; $[C_{25}H_{49}N_9O_6+2H]^{2+}$ 286.69, found 286.85; $[C_{25}H_{49}N_9O_6+ACN+2H]^{2+}$ 307.21, found 307.30.

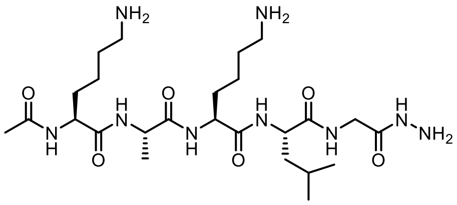

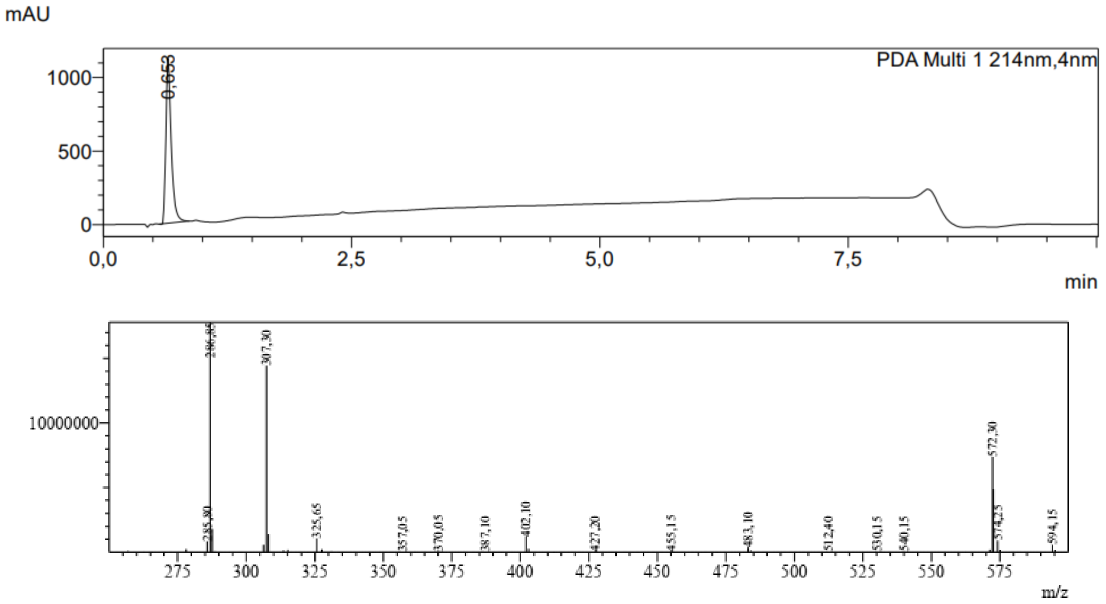

Figure S39: LC/MS analysis: HPLC chromatogram (top) and HR-ESI-MS spectrum (bottom).

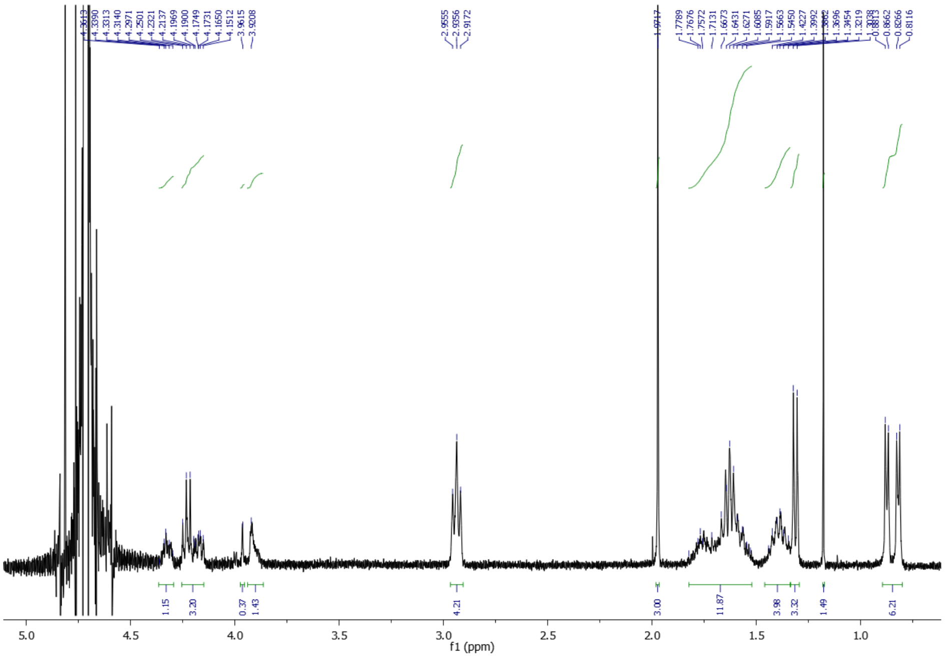

Figure S40: ^{1}H NMR (400 MHz, D_2O) spectrum of **KAKLG-Hyd.**

KFKLG-Hyd. The modified amino acid was synthesized using Fmoc-*L*-Leu-OH, Fmoc-*L*-Phe-OH, Fmoc-Gly-OH and Fmoc-*L*-Lys(Boc)-OH. The crude product was obtained after precipitation in Et_2O and freeze drying. The compound **KFKLG-Hyd** was isolated by preparative HPLC purification (0% B for 5 min, then → 40% B in 40 min). Yield: 29 %. ^1H NMR (400 MHz, D_2O): δ = 7.41 – 7.31 (m, 3H, H_{ar}), 7.27 – 7.29 (m, 2H, H_{ar}), 4.66 (dd, *J* = 6.2, 8.4, 1H, H_α), 4.36 (dd, *J* = 4.6, 9.5, 1H, H_α), 4.30 (dd, *J* = 6.4, 8.3, 1H, H_α), 4.14 (dd, *J* = 5.8, 8.5, 1H, H_α), 3.99, (d, *J* = 2.2, 2H, H_αGly), 3.17 - 2.93 (m, 6H, *C*H_2Phe and *C*$H_2$$NH_2$), 2.03 (s, 3H, CH_3), 1.71 – 1.58 (m, 12H, CH_2, CH), 1.38 – 1.30 (m, 3H, CH_2, CH), 0.97 (d, *J* = 6.0, 3H, *C*H_3CH), 0.92 (d, *J* = 5.9, 3H, *C*H_3CH); LC/MS: t_R 1.7 min; ESI-MS calcd for m/z $[C_{31}H_{53}N_9O_6+H]^+$ 648.41, found 648.55; $[C_{31}H_{53}N_9O_6+2H]^{2+}$ 324.71, found 324.85; $[C_{31}H_{53}N_9O_6+ACN+2H]^{2+}$ 345.22, found 345.35; $[C_{31}H_{53}N_9O_6+ACN-H]^-$ 646.69, found 646.15.

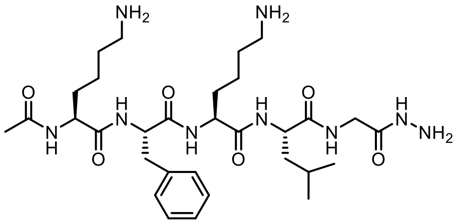

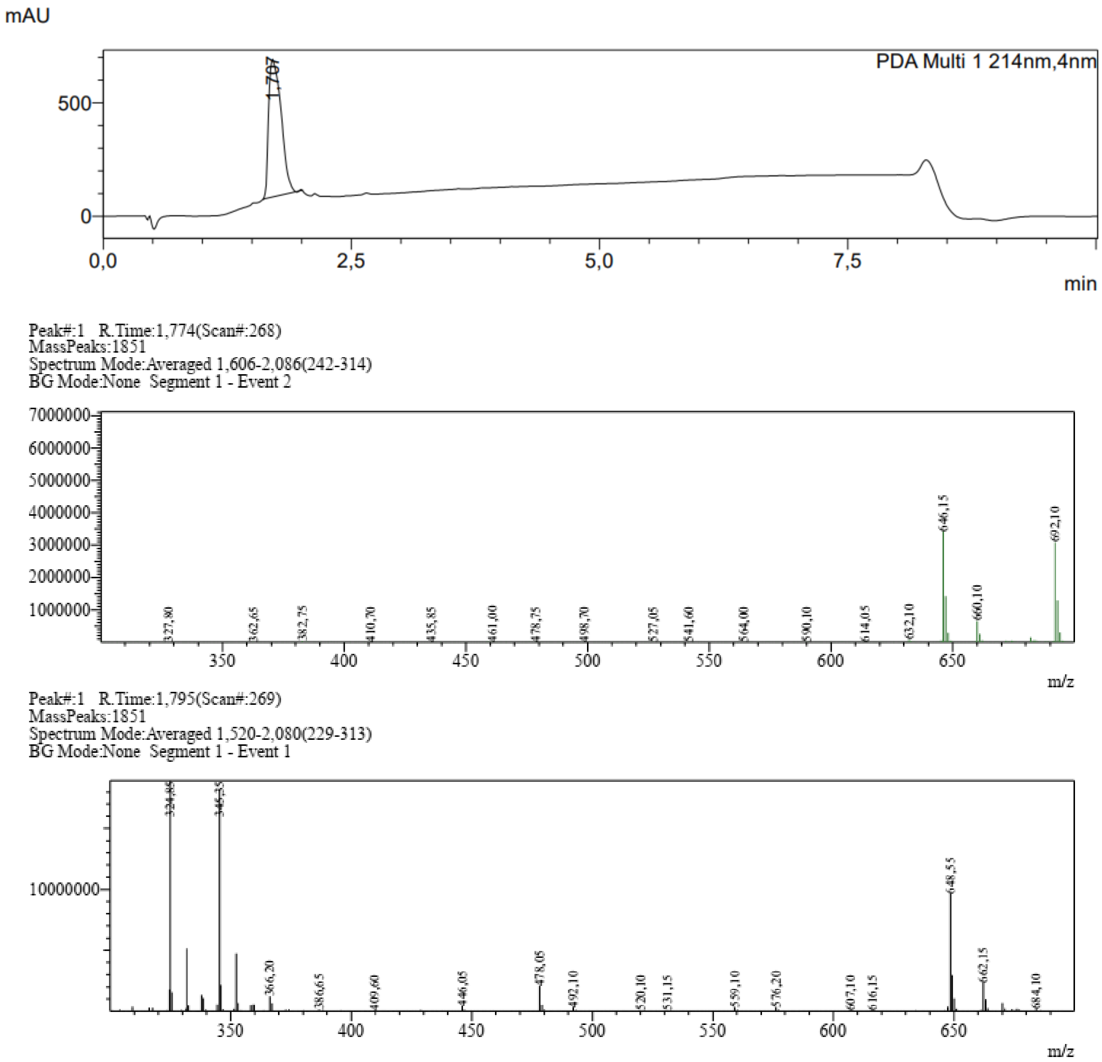

Figure S41: LC/MS analysis: HPLC chromatogram (top) and HR-ESI-MS spectrum (middle: negative mode; bottom: positive mode).

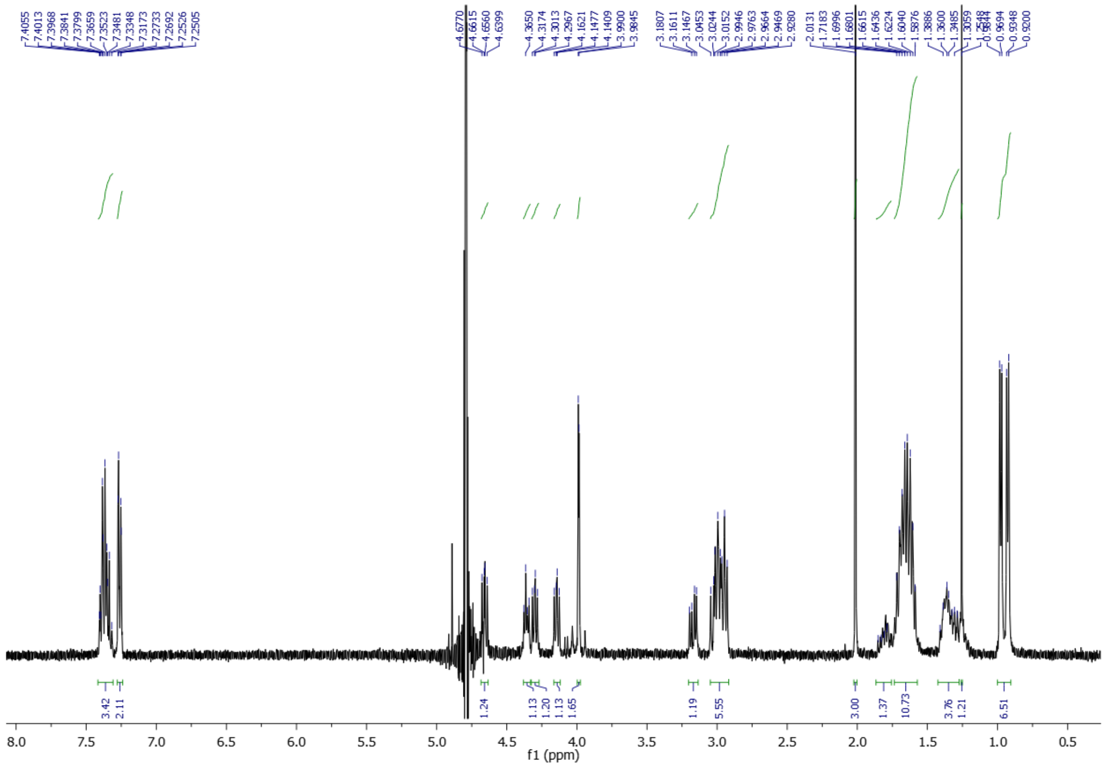

Figure S42: ^{1}H NMR (400 MHz, D_2O) spectrum of **KFKLG-Hyd.**

KSKLG-Hyd. The modified amino acid was synthesized using Fmoc-*L*-Leu-OH, Fmoc-*L*-Ser(O*t*Bu)-OH, Fmoc-Gly-OH and Fmoc-*L*-Lys(Boc)-OH. The crude product was obtained after precipitation in Et_2O and freeze drying. The compound **KSKLG-Hyd** was isolated by preparative HPLC purification (0% B for 5 min, then → 40% B in 40 min). Yield: 43 %. ^{1}H NMR (400 MHz, D_2O): δ = 4.44 (t, *J* = 5.6, 1H, Hα), 4.40 – 4.34 (m, 2H, Hα), 4.29 (dd, *J* = 5.7, 8.9, 1H, Hα), 3.99 (s, 2H, Hα), 3.93 – 3.83, (ddd, *J* = 4.8, 10.8, 16.3, 2H, C*H*$_2$OH), 3.09 (t, *J* = 8.7, 4H, C*H*$_2$NH$_2$), 2.09 (s, 3H, CH$_3$), 1.83 – 1.43 (m, 15H, CH$_2$, CH), 0.95 (d, *J* = 6.0, 3H, C*H*$_3$CH), 0.89 (d, *J* = 6.2, 3H, C*H*$_3$CH); LC/MS: t_R 0.61 min; ESI-MS calcd for m/z $[C_{25}H_{49}N_9O_7+H]^+$ 588.37, found 588.50; $[2(C_{25}H_{49}N_9O_7)+H]^+$ 1175.80, found 1175.30; $[C_{25}H_{49}N_9O_7+2H]^{2+}$ 294.68, found 294.85; $[C_{25}H_{49}N_9O_7+CAN+2H]^{2+}$ 315.22, found 315.25.

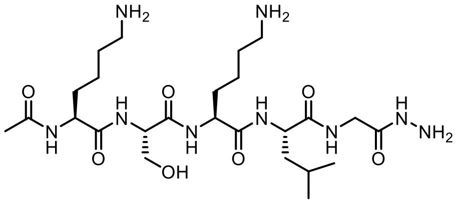

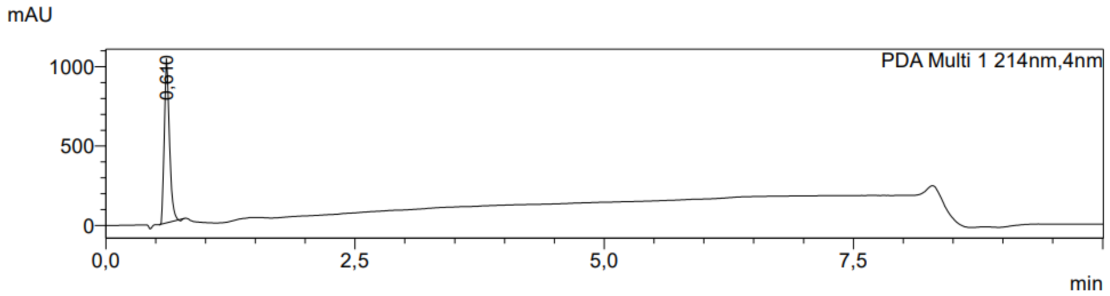

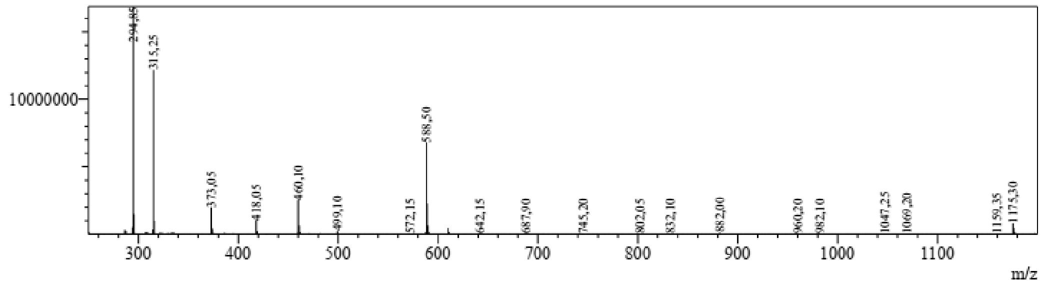

Figure S43: LC/MS analysis: HPLC chromatogram (top) and HR-ESI-MS spectrum (bottom).

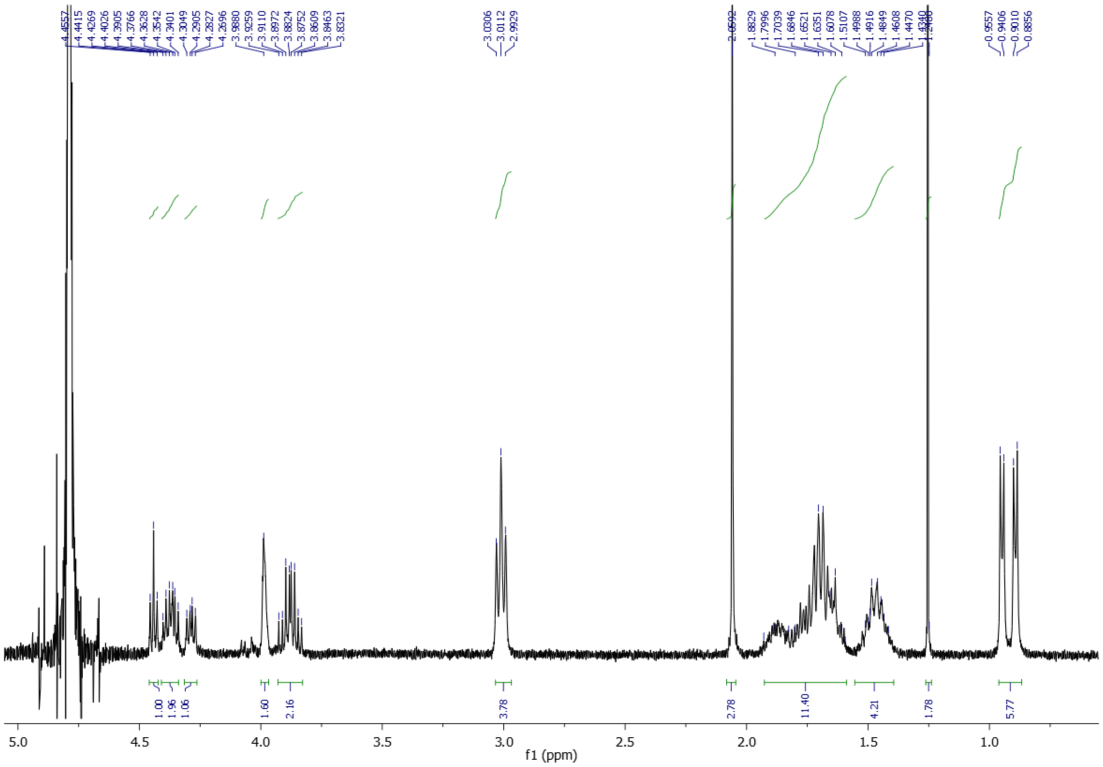

Figure S44: ^{1}H NMR (400 MHz, D_2O) spectrum of **KSKLG-Hyd.**

5. Conjugation reaction with C1-Hyd

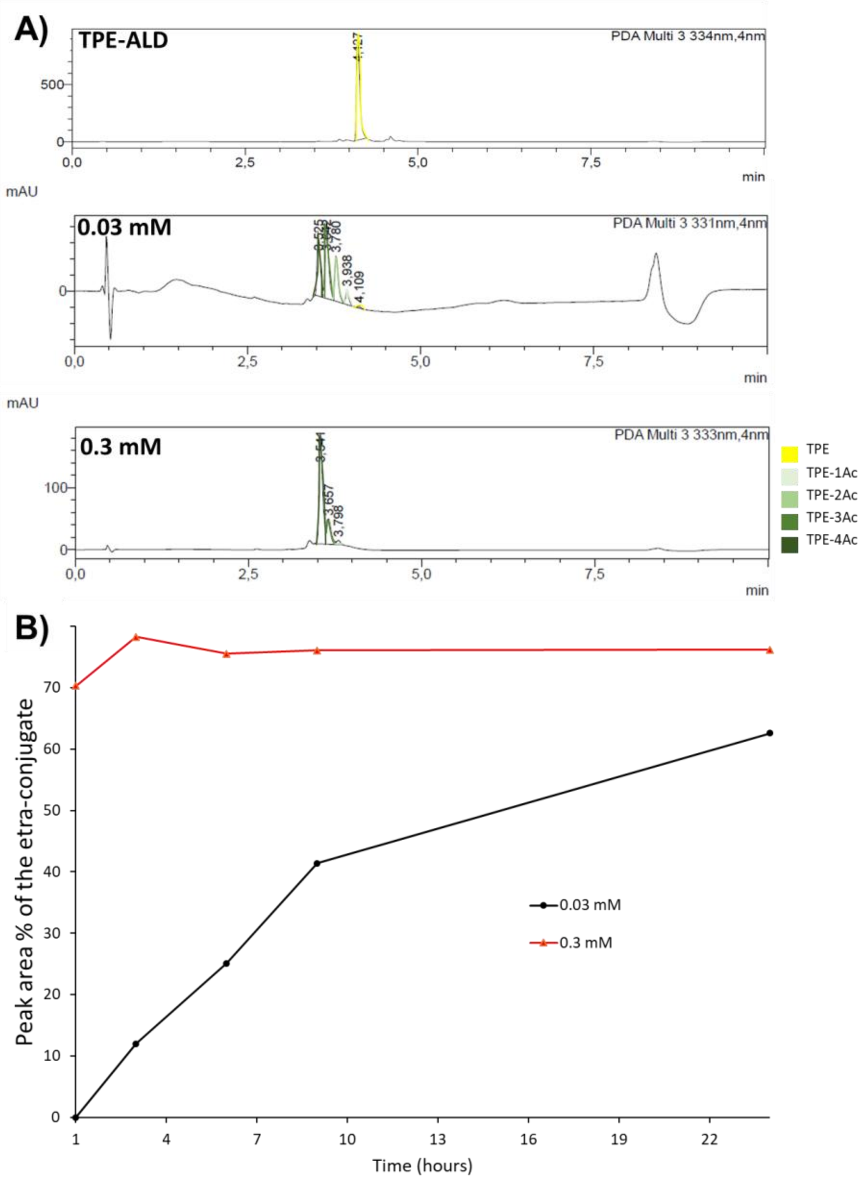

Figure S45: A) HPLC chromatograms after 9 hours of reaction between **TPE-Ald** and **C1-Hyd** (4 equivalents) in sodium acetate buffer (100 mM, pH 5) at 0.03 mM and 0.3 mM of **TPE-Ald**; B) plot of the time evolution of the peak area percentage of the tetra-conjugate **TPE(C1)$_4$**.

6. Screening assays of aliphatic and gallic hydrazide derivatives

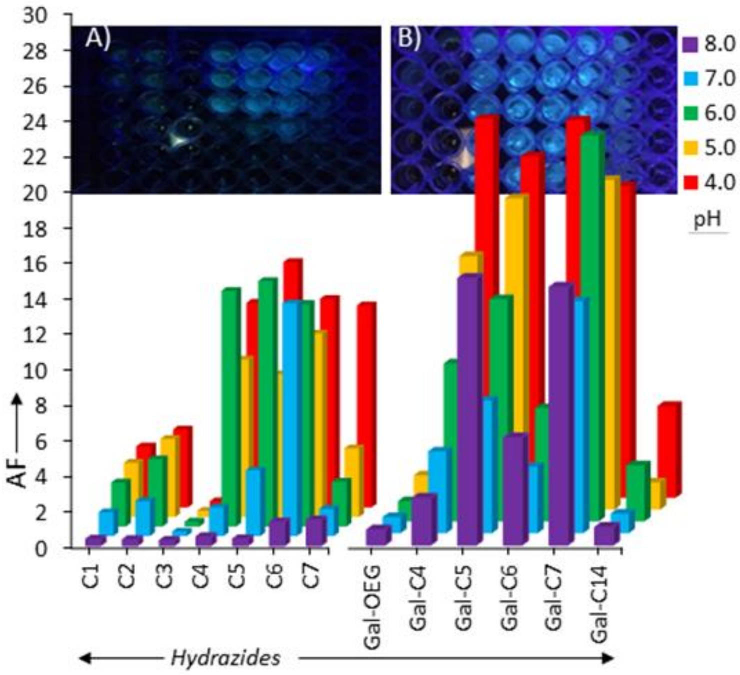

Figure S46: Combinatorial screening in 96-well plates detecting fluorescence enhancement (amplification factor AF) when combining **TPE-Ald** (0.3 mM) with different aliphatic (A) and gallic hydrazide (B) derivatives at different pH. λ_{exc} = 330 nm, λ_{em} = 510 nm. Incubation time: 24 hours. The photographs of the plates were taken under UV-light irradiation.

7. Self-assembly of Ben-Hyd and TPE-Ald

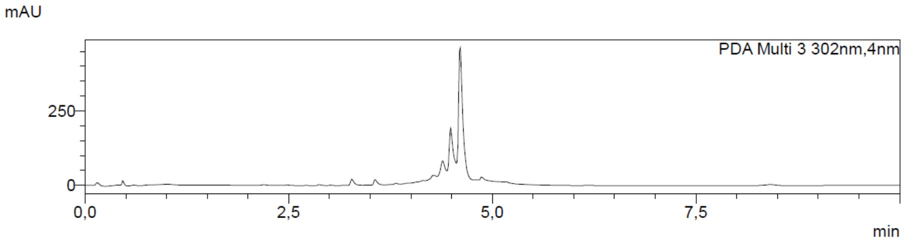

Figure S47: Representative HPLC chromatogram of the conjugation between **TPE-Ald** and **BenHyd** (4 equivalents) in sodium acetate buffer (100 mM, pH 5).

8. Time evolution of the self-assembly of Ben-Hyd and TPE-Ald

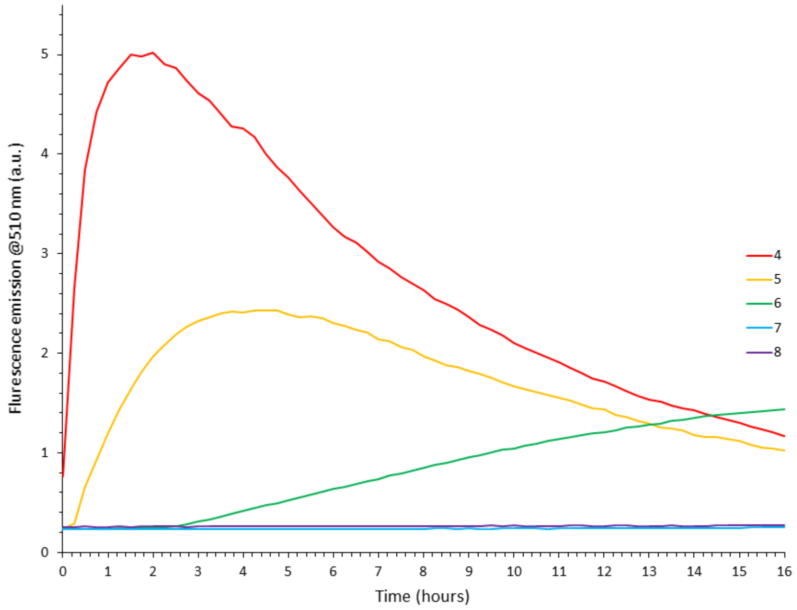

Figure S48: Time evolution of the fluorescence emission at different pH during the self-assembly of **TPE-Ald** (0.03 mM) with **BenHyd**.

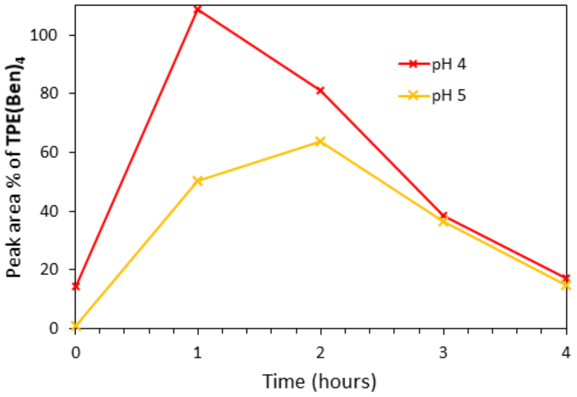

Figure S49: Time evolution of the HPLC peak area % of **TPE(Ben)$_4$**, during the self-assembly of **TPE-Ald** (0.03 mM) with **BenHyd** at pH 4 and 5.

9. Time evolution of the self-assembly of F_2-Hyd and TPE-Ald

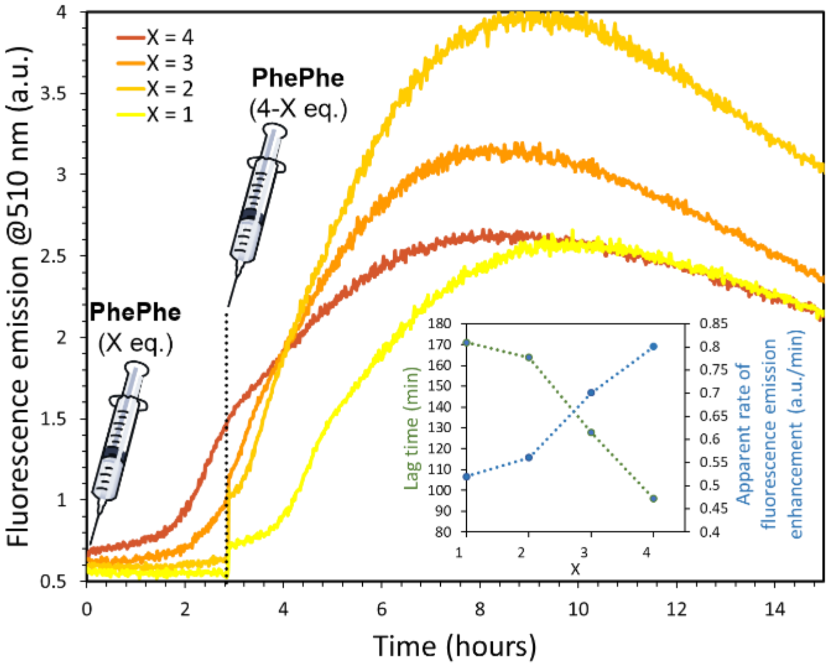

Figure S50: Evolution of fluorescence emission during sub-stoichiometric self-assembly of **TPE-Ald** (0.03 mM) with **F_2Hyd** at pH 5. The stoichiometry was completed to four by a second addition of **F_2Hyd** after 3 hours of reaction (dotted line). The inset represents the lag time and apparent rate of fluorescence emission enhancement in the initial phase for various X=1-4 with a linear fit to guide the eye.

10. Competition experiment between C1-Hyd and BenHyd

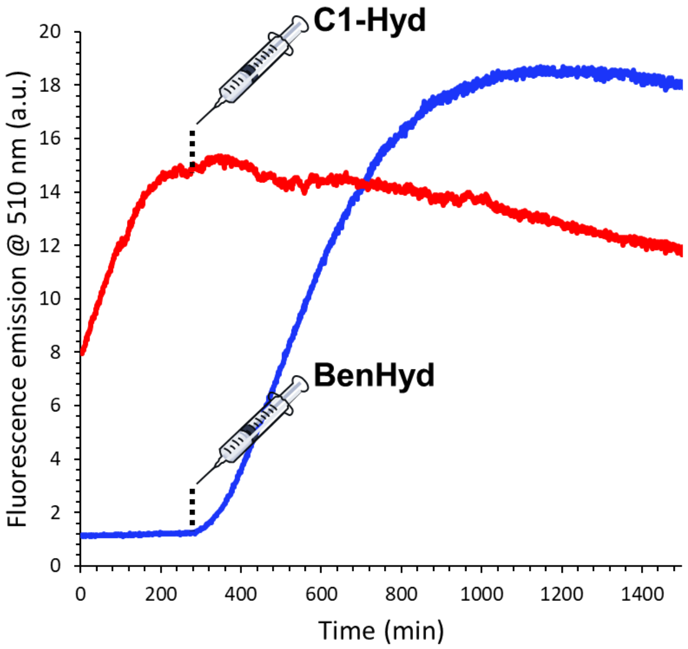

Figure S51: Monitoring by fluorescence spectroscopy of the sequential addition at 300 min (5 hours) of **C1-Hyd** and **BenHyd** onto, respectively, the reaction between **TPE-Ald** (0.03 mM) and **C1-Hyd** (blue curve) or **BenHyd** (red curve), at pH 5.

11. Spectral evolution in the self-assembly of Ben-Hyd and TPE-Ald

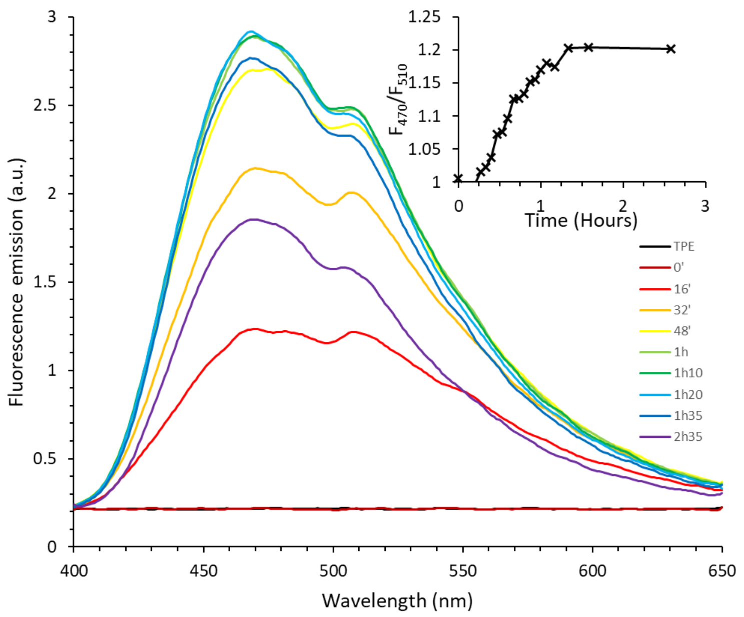

Figure S52: Time evolution of the fluorescence emission upon mixing **TPE-Ald** (0.3 mM) with **BenHyd** at pH 5.0. λ_{exc} = 330 nm. The inset plots the ratio of intensities of fluorescence emission at 470 and 510 nm.

12. Spectral evolution in the self-assembly of F_2-Hyd and TPE-Ald

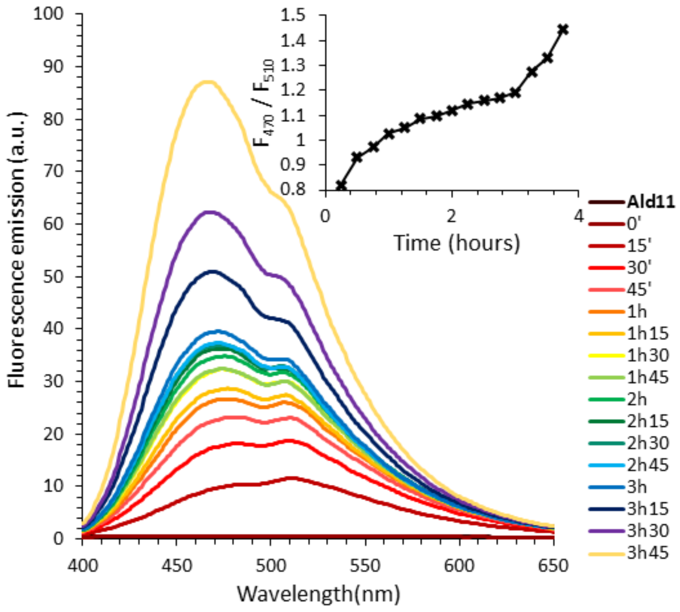

Figure S53: Time evolution of the fluorescence emission upon mixing **TPE-Ald** (0.3 mM) with **F_2-Hyd** at pH 5.0. λ_{exc} = 330 nm. The inset plots the ratio of intensities of fluorescence emission at 470 and 510 nm.

13. Self-assembly of GLKFK-Hyd and TPE-Ald

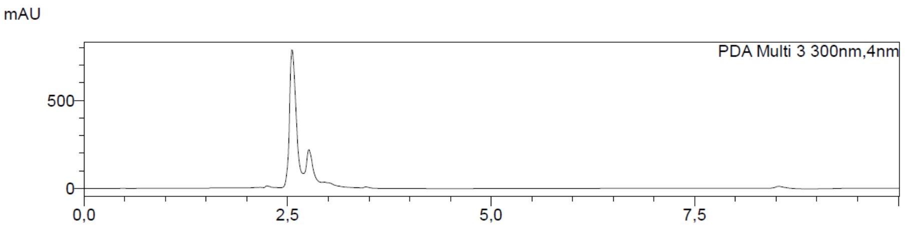

Figure S54: Representative HPLC chromatogram of the conjugation between **TPE-Ald** and **GLKFK-Hyd** (4 equivalents) in sodium acetate buffer (100 mM, pH 5) at 0.3 mM of **TPE-Ald**.

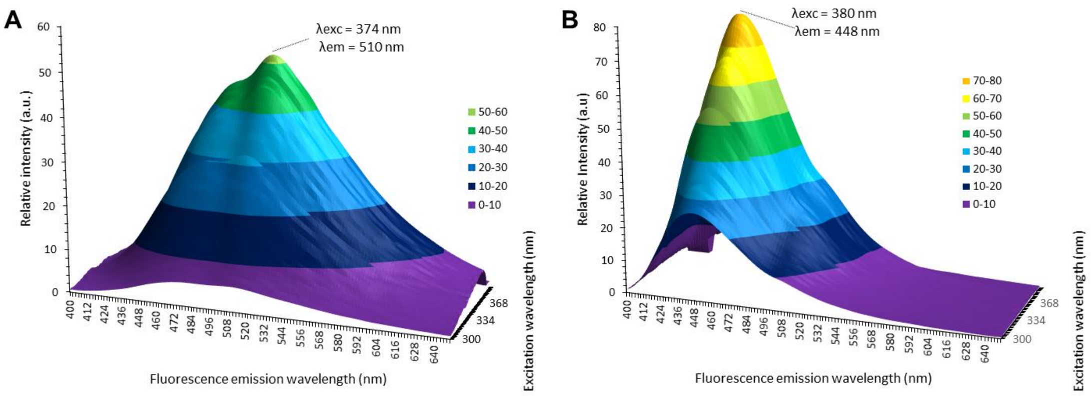

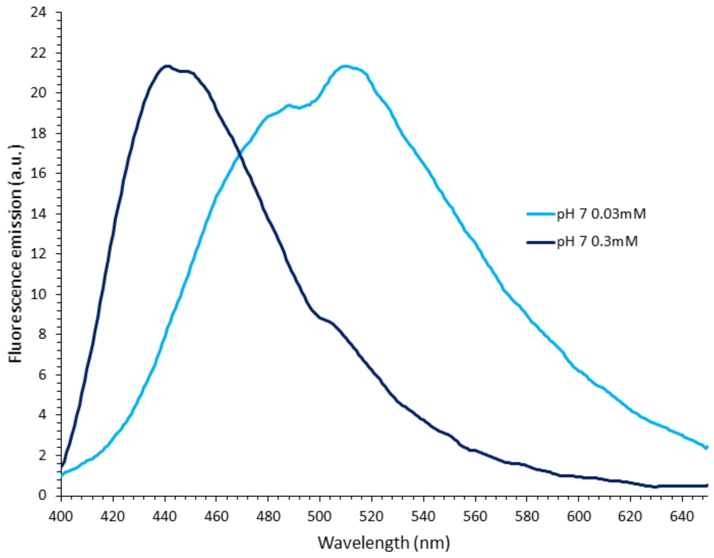

Figure S55: Top: 3D absorption-emission spectra of the assembly, at pH 7, of **TPE-Ald** (A: 0.03 mM; B: 0.3 mM) and **GLKFK-Hyd** after 24 hours; bottom: corresponding fluorescence emission spectra.

14. CD spectroscopy

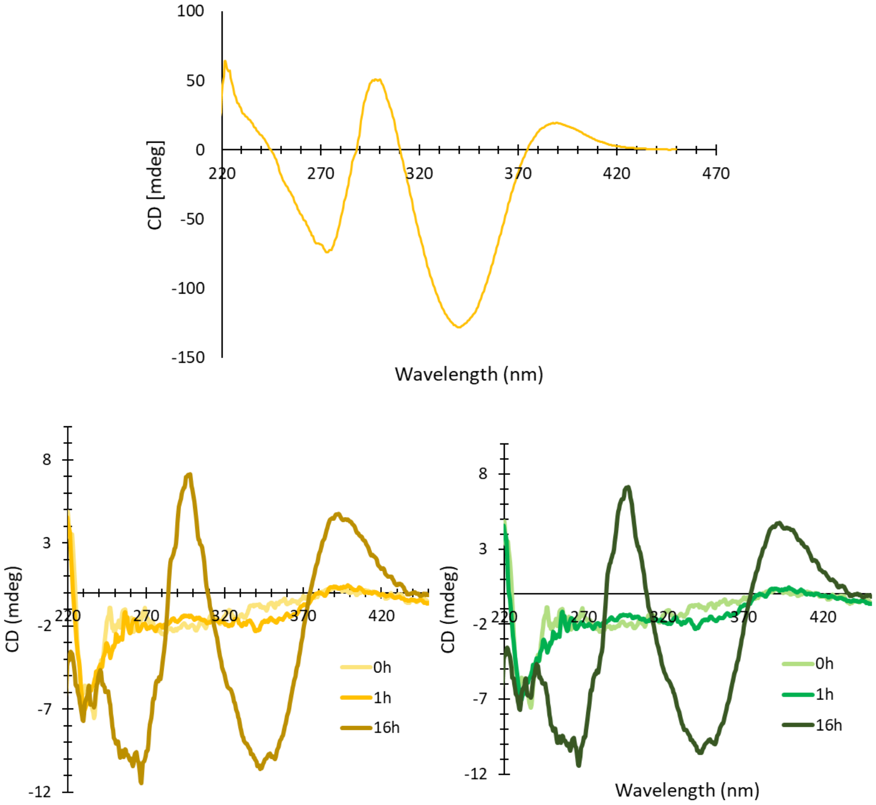

Figure S56: CD spectra of the self-assembly of **TPE-Ald** (0.3 mM) with i) **GLKFK-Hyd** at pH 5 (top), and ii) **KFKLG-Hyd** at pH 5 (bottom left) and pH 7 (bottom right), measured initially, after 1 hour, and 16 hours.

15. Self-assembly of GLKFK-Hyd and Benzaldehyde

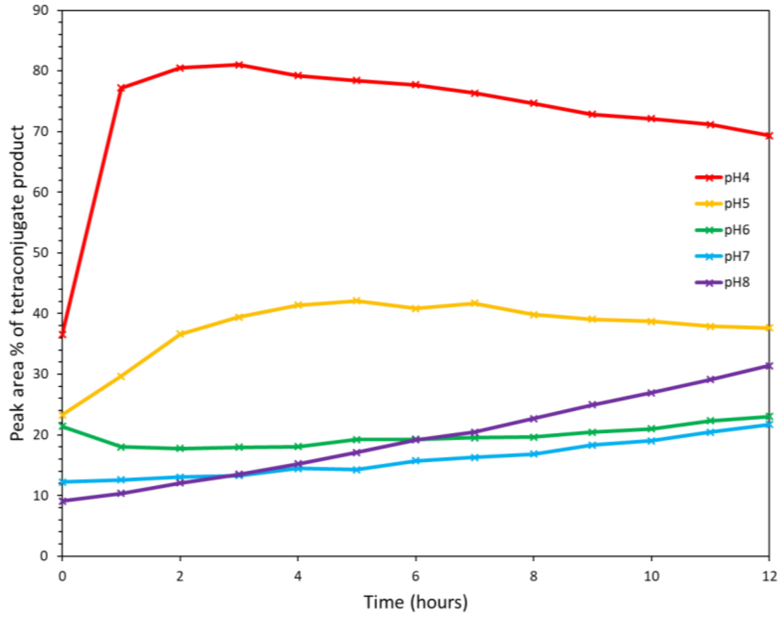

Figure S57: Relative proportion of the tetraconjugate product formed during the self-assembly of benzaldehyde with **GLKFK-Hyd**, at different pH.

16. Self-assembly of KFKLG-Hyd and TPE-Ald

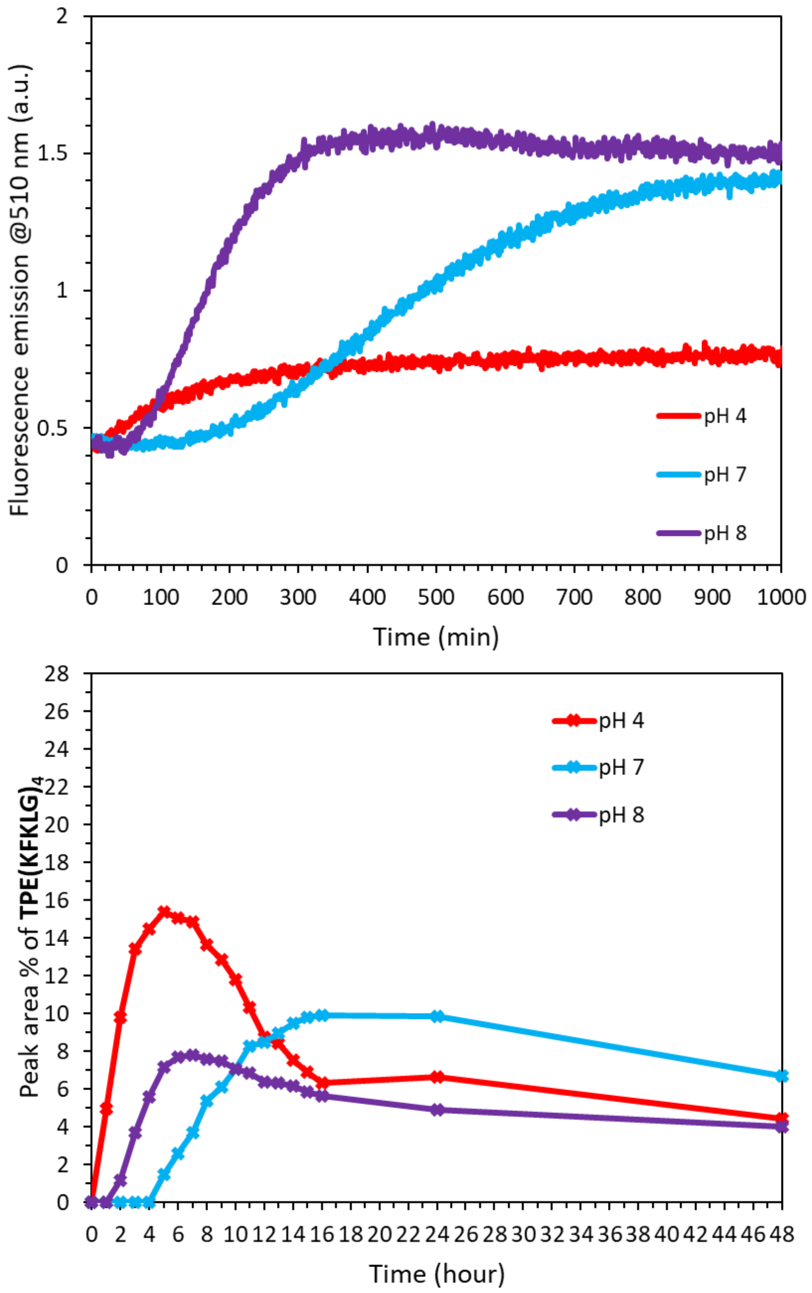

Figure S58: Time evolution of the coupling of **TPE-Ald** (0.03 mM) with **KFKLG-Hyd** at different pH: A) fluorescence emission, B) relative proportion of **TPE(KFKLG)$_4$**.

17. References

1 S. W. Kelemu, C. M. Fitchett and P. J. Steel, *Tet. Lett.*, 2014, **55**, 2019-2021.

2 J. B. Xiong, H. T. Feng, J. P. Sun, W. Z. Xie, D. Yang, M. H. Liu and Y. S. Zheng, *J. Am. Chem. Soc.*, 2016, **138**, 11469-11472.

3 P. Ahlers, H. Frisch and P. Besenius, *Polym Chem-Uk*, 2015, **6**, 7245-7250.

4 D. V. Nguyen, L. Hugoni, M. Filippi, F. Perton, D. Shi, E. Voirin, L. Power, G. Cotin, M. P. Krafft, A. Scherberich, P. Lavalle, S. Begin-Colin and D. Felder-Flesch, *New J. Chem.*, 2020, **44**, 3206-3214.

5 X. F. Zhang, B. Koz, H. K. Bisoyi, H. Wang, K. G. Gutierrez-Cuevas, M. E. McConney, T. J. Bunning and Q. Li, *Acs Appl Mater Inter*, 2020, **12**, 55215-55222.

6 R. L. Zhang, H. F. Gao, J. Yu, C. Liu, Y. Tao and X. H. Cheng, *J. Mol. Liq.*, 2020, **298**, 112079.

7 V. S. Sharma, A. P. Shah and A. S. Sharma, *New J. Chem.*, 2019, **43**, 3556-3564.

8 X. W. Peng, H. F. Gao, Y. L. Xiao, H. F. Cheng, F. R. Huang and X. H. Cheng, *New J. Chem.*, 2017, **41**, 2004-2012.

9 M. Albrecht, I. Latorre, G. Mehmeti, K. Hengst and I. M. Oppel, *Dalton Trans.*, 2011, **40**, 12067-12074.

10 P. Iqbal, M. Mayanditheuar, L. J. Childs, M. J. Hannon, N. Spencer, P. R. Ashton and J. A. Preece, *Materials*, 2009, **2**, 146-168.

11 E. Suárez-Picado, M. Coste, J.-Y. Runser, M. Fossépré, A. Carvalho, M. Surin, L. Jierry and S. Ulrich, *Biomacromolecules*, 2022, **23**, 431-442.

12 E. Bartolami, Y. Bessin, V. Gervais, P. Dumy and S. Ulrich, *Angew. Chem. Int. Ed.*, 2015, **54**, 10183-10187.

13 E. Bartolami, Y. Bessin, N. Bettache, M. Gary-Bobo, M. Garcia, P. Dumy and S. Ulrich, *Org. Biomol. Chem.*, 2015, **13**, 9427-9438.
